# Synthesis of Pandaroside D from *Pandaros acanthifolium* via Construction of a Rare Enone System in the Steroid D Ring

**DOI:** 10.1021/acs.orglett.5c01375

**Published:** 2025-08-26

**Authors:** Karol Michalak, Robert Bujok, Piotr Cmoch, Jacek Mlynarski

**Affiliations:** Institute of Organic Chemistry, 154690 Polish Academy of Sciences, Kasprzaka 44/52, 01-224 Warsaw, Poland

## Abstract

All marine saponins isolated from *Pandaros acanthifolium* feature a unique and distinctive enone system located in the D ring and an unusual *cis*-C/D ring junction, presenting an intriguing yet unattained synthetic challenge. The first total synthesis of pandaroside D (**1**) and its methyl ester (**2**) was achieved from commercially available dehydroisoandrosterone acetate (DHEA). A notable feature of this synthesis is direct oxidation of the C-15 position promoted by the Davis reagent. This concept was applied despite the negative results of such an attempt described in the literature and confirmed the possibility of using the reagent in the direct construction of the enone system in ring D. For the final step, the reaction with sugar trichloroacetimidate proved unpromising; however, glycosylation of synthesized aglycone was successfully achieved using the classical Koenigs–Knorr variant with methyl 2,3,4-tri-*O*-acetyl-β-d-glucuronate bromide.

## Graphical abstract

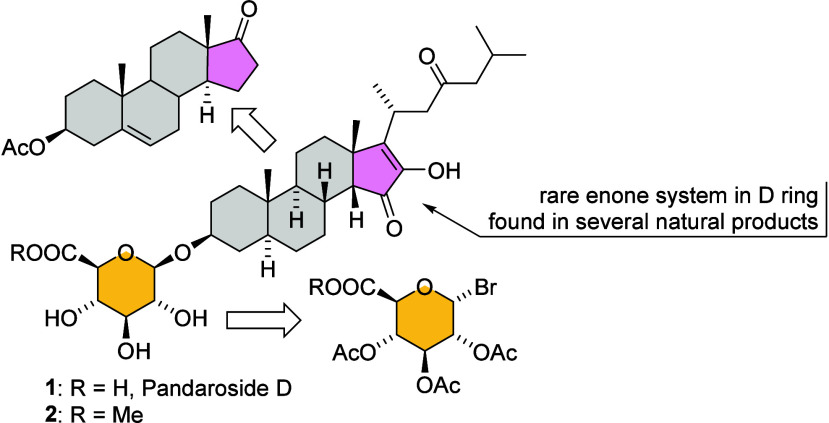

Although steroids, including saponins, have been recognized for their significance for quite some time,^1–3^ their synthesis continues to attract considerable interest and ongoing development.^4^ This is driven both by the structural complexity of these compounds and the increasing demand for samples of biologically active compounds.^5–7^ Marine sponges,^8^ particularly those from the order Poecilosclerida and genus *Pandaros*, are rich sources of steroidal compounds. In 2009, a detailed chemical analysis of the Caribbean sponge *Pandaros acanthifolium* led to the identification of seven novel steroidal glycosides, designated as pandarosides A–D, alongside the methyl esters of pandarosides A, C, and D.^9^ Later, a careful chemical reinvestigation of the *P. acanthifolium* extract led to the isolation of six new steroidal saponins, pandarosides K–M, and their methyl esters as minor components.^10^ Their structures were determined through spectroscopic analyses and by comparing the data to those of previously identified metabolites in this family. Interestingly, pandarosides A–D and their methyl esters are all characterized by a rare 2-hydroxycyclopentenone D ring with a 14β configuration, a *cis*-C/D ring junction, and a C-23 ketone (Scheme 1). Given the observed bioactivity^10^ of this family of compounds and particularly their unique structure, we decided to carry out the synthesis of a representative example, pandaroside D (Scheme 1). To the best of our knowledge, there are no reports in the literature regarding the synthesis of steroids containing an enone system in the D ring, as found in pandarosides.^11^

**Scheme 1:**
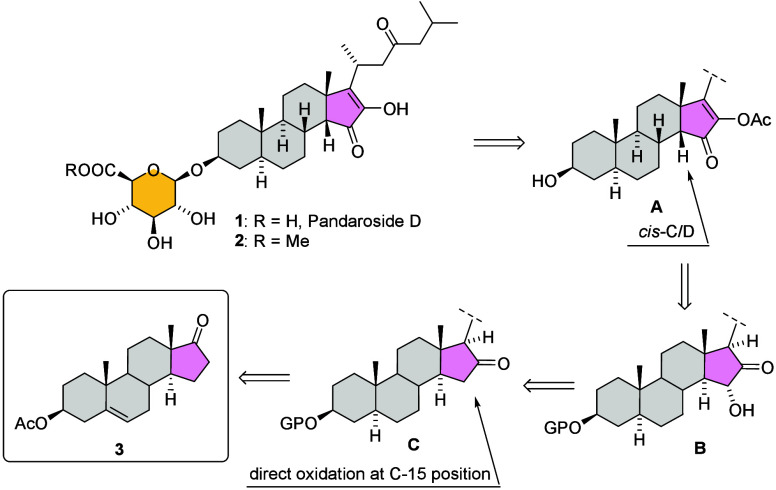
Retrosynthetic Analysis

We planned our synthesis starting from commercially available dehydroisoandrosterone acetate (DHEA, **3**), with the key step involving direct oxidation of the C-15 position in the D ring. This reaction had been previously reported as unsuccessful, likely due to the large size of the reagent, which prevented it from effectively approaching the substrate.^12^ In this work, we demonstrate that it is indeed possible, and such a transformation can be successfully applied to the synthesis of steroids and saponins.

Retrosynthetic analysis of pandaroside D (**1**) suggested that alcohol (**4**) would be a suitable substrate for the steroid core, as its synthesis from commercially available dehydroisoandrosterone acetate (**3**) is well-documented in the literature (Scheme 2).^13^ Our synthesis of pandaroside D comprises then three consecutive key steps: modification of the side chain in **4**, alteration of the D ring, and final glycosylation of the resulting steroid using a protected glucuronic acid derivative. We then began with the synthesis of an alcohol (**4**). Compound **4** was prepared from DHEA acetate following a literature protocol, and after six steps, the desired product was isolated with an overall yield of 56%. With this compound in hand, we were able to proceed with the construction of the side chain, as depicted in Scheme 2.

**Scheme 2:**
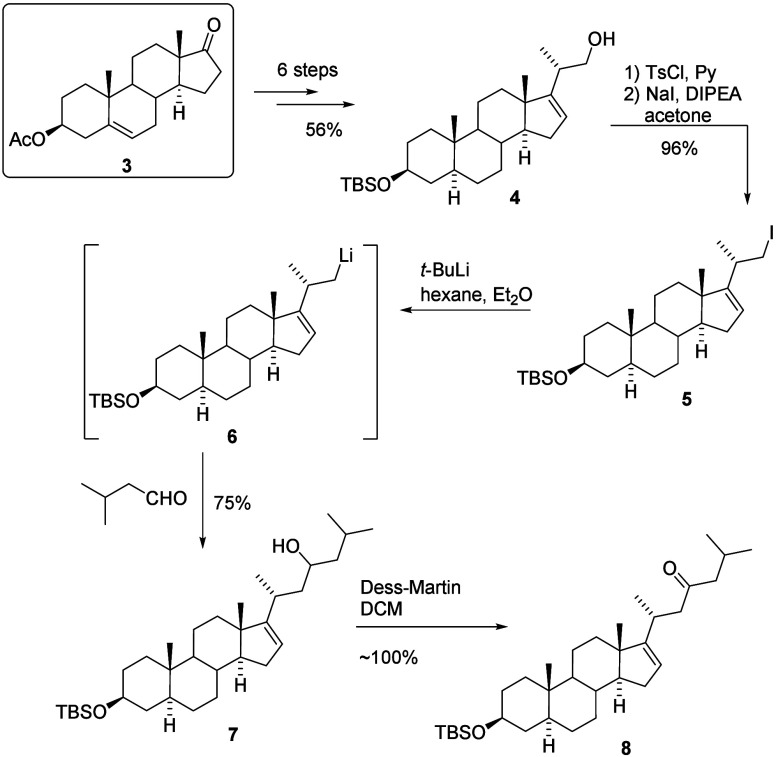
Construction of the Side Chain

The alcohol (**4**) was transformed into the corresponding tosylate and subsequently into iodide (**5**) in an excellent yield of 96%. To prevent the hydrolysis of the TBDMS ether, the final substitution reaction was carried out in the presence of a small amount of DIPEA (Scheme 2). The organolithium intermediate (**6**) was then generated using *tert*-butyllithium at −78 °C. This species upon the addition of isovaleryl aldehyde was transformed into a mixture of two epimeric alcohols (**7**) in a 4:5 ratio, which was obtained with a 75% yield. Obtaining a mixture of stereoisomers was not an issue, as the next planned step involved converting the alcohol into a ketone, thereby eliminating the stereogenic center. Thus, a mixture of isomers was smoothly oxidized to the desired key intermediate (**8**) with a quantitative yield. With this step, the first part of the synthesis, modification of the side chain, was successfully completed.

The next steps in the synthesis of pandaroside D involved modification of the D ring. Our plan was to oxidize position 16, followed by position 15, along with subsequent equilibrium-driven epimerization of the system, to achieve the correct stereochemistry at the ring junction. We envisioned that a hydroxyl group could be introduced at position 15 of compound **11**, which should be readily accessible from the prepared compound **8** through alcohol (**10**; Scheme 3). To realize this idea and introduce a hydroxyl group at position 16, we selected the hydroboration–oxidation method. To prevent the reduction of ketone during the reaction with BH_3_·Me_2_S, the ketone moiety was protected as an acetal by using ethylene glycol. Unfortunately, under the reaction conditions, we observed partial cleavage of the silyl ether. Therefore, the crude product after the formation of the acetal moiety was treated again with TBDMSCl, and the desired compound **9** was isolated in excellent yield (98%). The essential hydroboration of this intermediate with BH_3_·Me_2_S, followed by oxidation with H_2_O_2_ under basic conditions, proceeded smoothly. Isolated alcohol (**10**, 77% yield) was subsequently converted into ketone (**11**) in a quantitative yield (Scheme 3).

**Scheme 3:**
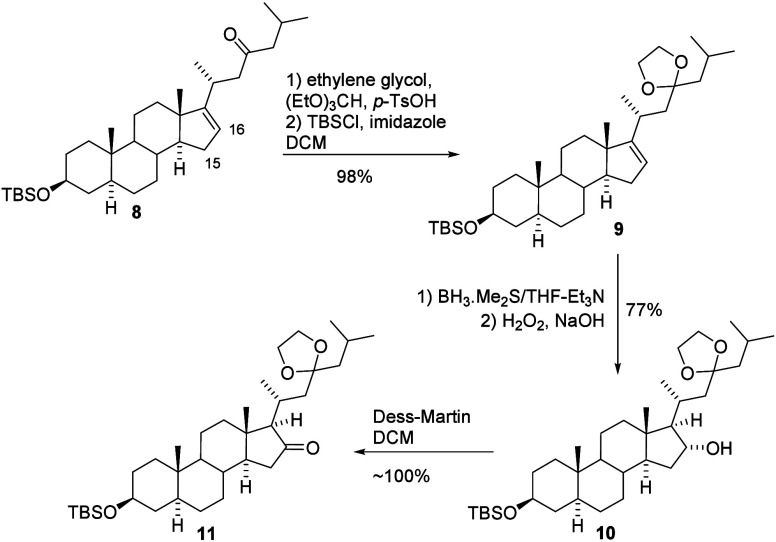
Oxidizing of Position C-16

We have now reached the most challenging transformation, which does not have a clear background in the literature. To introduce a hydroxyl group at position 15, we initially planned to use the Davis reagent [3-phenyl-2-(phenylsulfonyl)-1,2-oxaziridine]. However, Williams and co-workers reported unsuccessful attempts to direct hydroxylation at this position in a similar ring system with the Davis reagent during the synthesis of the aglycone of 26-*O*-deacetyl pavoninin-5.^12^ The authors suggested that the disappointment was due to steric hindrance of the Davis reagent, which is why they employed a non-direct two-step synthesis to introduce the hydroxyl group at position 15. They first transformed the ketone into a silyl enol ether, which was then oxidized with dimethyldioxirane to yield the 15-hydroxysteroid in an overall 49% yield.

Our initial attempts to reproduce the reaction using more manageable oxone instead of pure dimethyldioxirane were unsuccessful. Only 27% of the desired product was obtained when a TBDMS group rather than a trimethylsilyl group was used in the silyl enol ether. Other attempts were also disappointing; therefore, we ultimately decided to revisit the previously used conditions and test direct hydroxylation with the Davis reagent. Contrary to the results described by Williams and co-workers, using NaHMDS as the base and Davis reagent, we successfully obtained the expected hydroxyketone (**12**). Thus, it turned out that our plan could be realized through a simple transformation, and this observation is extremely valuable in steroid synthesis. Dess–Martin oxidation of alcohol (**12**) delivered kinetic ketoenol (**13**) with an overall yield of 60% (Scheme 4). The configuration of the C-15 stereocenter was not determined however. Our earlier works suggested that the 15*R* isomer should be formed predominantly (as marked in Scheme 4), but it was not confirmed. It should be stressed that, in next step, the hydroxyl group was oxidized to ketone (or rather corresponding enol); therefore, the configuration of the C-15 stereocenter in compound **12** does not matter from the view of the synthesis of pandaroside D.

**Scheme 4:**
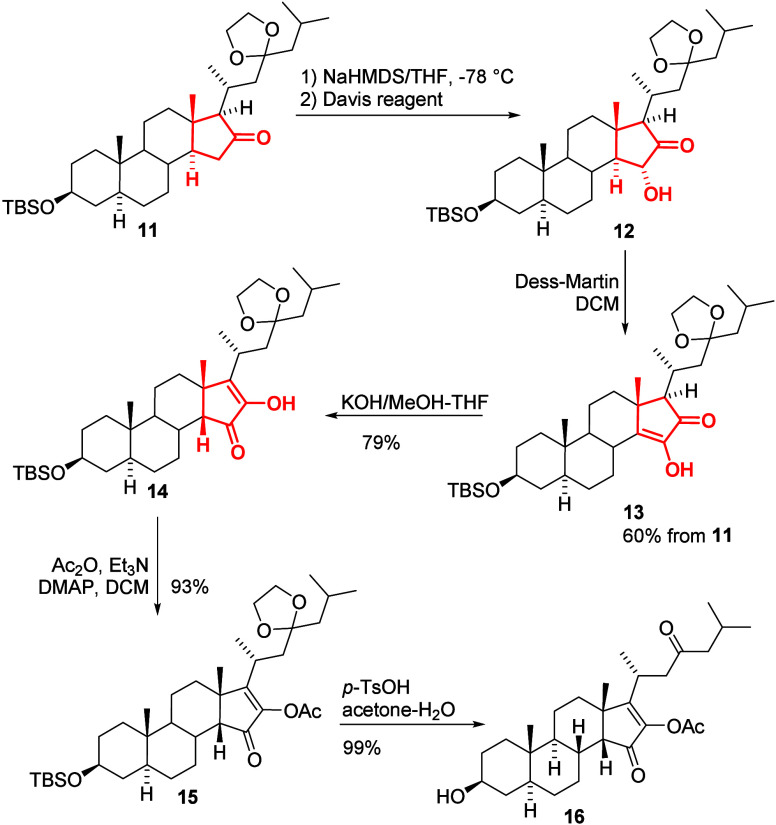
Construction of the Desired Enone System in the D Ring

We assumed that the kinetic isomer (**13**) under basic conditions would transform into the more stable isomer (**14**). Indeed, by using KOH in a mixture of methanol and THF, the expected isomer was formed. The synthesis of an unusual enone in the D ring was then successfully completed. After protection of the free hydroxyl group as an acetate and deprotection of both the silyl ether and acetal moiety, compound (**16**) was obtained, ready for glycosidation.

Considering that pandarosides have never been synthesized before and that the literature only contains an analysis of material isolated from sponges,^9^ we decided to perform a full structural analysis of the obtained compound (**16**) at this stage. A complete set of two-dimensional NMR techniques unambiguously confirming the structure are provided in the Supporting Information.

The next and final step in the synthesis of pandaroside D was the attachment of glucuronic acid to aglycone (**16**). We initially decided to test the glycosylation reaction with trichloroacetimidate (**18**), which was easily obtained from a commercial methyl 1,2,3,4-tetra-*O*-acetyl-β-d-glucuronate (**17**; Scheme 5). This approach had previously been broadly used in the glycosylation of steroids, hence our choice of method.^14^ However, it is known from the literature that this method is not always effective and often results in low yields of the desired glycoside or fails to produce the expected product altogether.^15^

**Scheme 5:**
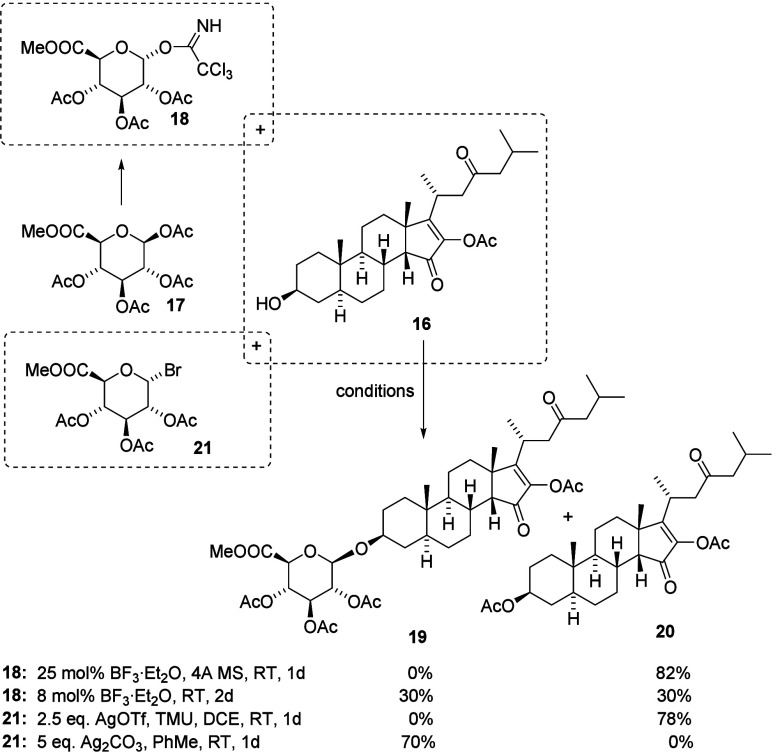
Glycosidation of Aglycone (**16**)

Given this information from the literature, we began by testing a model reaction between trichloroacetimidate (**18**) and cholesterol. This test confirmed the possibility of obtaining the desired glycoside with a 56% yield using BF_3_ etherate as an activator. In the case of TMSOTf, the yields ranged from 20 to 30%, depending on the amount of activator and the temperature. It is worth noting that the reaction proceeded very cleanly, and apart from the product, the unreacted steroid was observed in the reaction mixture, which could be isolated and reused. Encouraged by these results, we carried out the reaction between aglycone (**16**) and trichloroacetimidate (**18**) under the previously established conditions. Unfortunately, in the case of the substrate for pandaroside D (**16**), only the acetate of the steroid (**20**) was formed, and no traces of the glycosylation product (**19**) were observed. The result is similar to that published by Stachulski and co-workers^15^ and serves as further evidence that the structure of the steroid is crucial for the yield of the glycosylation product.

A very thorough optimization of the reaction led to the formation of the desired product with only 30% yield after a prolonged reaction time. The key step was the removal of molecular sieves, which promoted the acetate transfer. Then, we decided to try the reaction of glucuronic acid bromide (**21**). Under weakly basic or nearly neutral conditions (AgOTf and tetramethylurea), only the steroid acetate (**20**) was formed, with a yield of 78% (Scheme 5). In contrast, using the significantly more basic silver carbonate and 2.5 equiv of bromide (**21**) under standard conditions (5.0 equiv of Ag_2_CO_3_, PhMe, room temperature, and 1 day) resulted in the isolation of the desired β-glycoside (**20**) in a good yield of 70%. The reaction results in exclusive formation of the β-anomer, which results from the influence of the acetyl group at the C-2 position of glucose and the steric bulk of the aglycone adopting an equatorial orientation; this outcome is consistent with the literature.^6^ Notably, the steroid acetate was not detected, even on thin-layer chromatography (TLC). These experiments confirm that acidic conditions favor the protecting group migration and formation of steroid acetate, whereas more basic conditions enhance the yield of the glycosidation product.

The preparation of the methyl ester of pandaroside D (**2**), which was also isolated from the *P. acanthifolium* sponge, required the global deprotection of four acetyl groups. This was easily accomplished by using the Zemplén method (Scheme 6). Hydrolysis of the methyl ester under more basic conditions resulted in the isolation of pandaroside D.

**Scheme 6:**
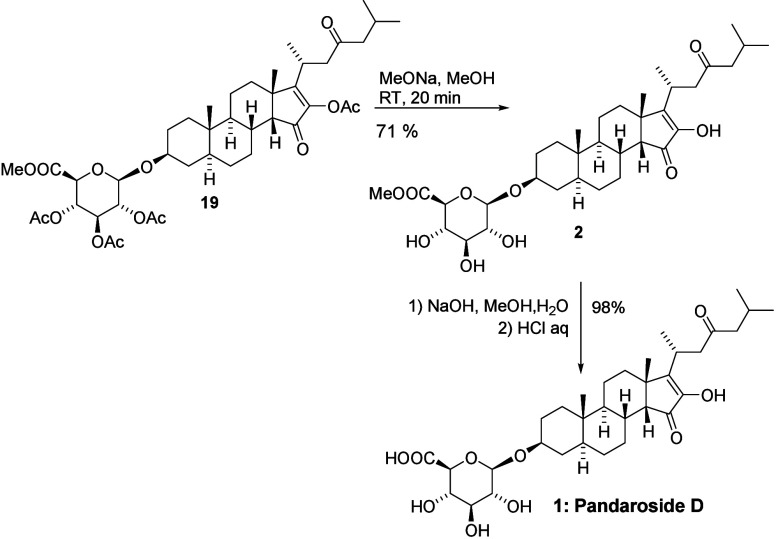
Final Deprotection of Product **19**

All analytical data: optical rotation and ^1^H and ^13^C NMR of the obtained methyl ester (**2**) are consistent with the literature data.^9^ For details, see the Supporting Information. A comparison of the spectra published in the literature to those of our samples revealed that the structure of pandaroside D methyl ester (**2**) isolated from *P. acanthifolium* is identical to that of the synthetically obtained sample. Unlike compound **2**, the spectrum of pandaroside (**1**) isolated from the marine sponge contains some additional signals, most likely corresponding to impurities. Taking into account that our compound **1** was obtained by hydrolysis from the unquestionable structure of methyl ester **2** and all spectra, ^1^H and ^13^C NMR and HR-MS, confirm this structure, we have no doubt that we have confirmed pandaroside D.

In summary, we presented that the first synthesis of pandaroside D (**1**) and its methyl ester (**2**) both isolated from *P. acanthifolium* was successfully achieved starting from commercially available DHEA. The synthesis fully confirmed the structure of the isolated compounds. A key step in the synthesis was the Davis-reagent-mediated direct oxidation at the C-15 position. This innovative application was successfully implemented despite previously reported negative outcomes in the literature, thereby demonstrating the viability of using the Davis reagent for constructing the specific enone system in ring D. This straightforward synthesis of a characteristic fragment found in many steroids and saponins could potentially be applied in practice in the future.

## Supplementary Material



## Data Availability

The data underlying this study are available in the published article and its online Supporting Information.

## Notes

Experimental details, spectroscopic data, and copies of ^1^H and ^13^C NMR spectra (PDF)
