# Supplementary material for: Synthesis of Pandaroside D from Pandaros acanthifolium via Construction of a Rare Enone System in the Steroid D Ring
Source: Org Lett. 2025 Aug 26;27(35):9571–5. doi: 10.1021/acs.orglett.5c01375 (PMC12418495; doi:10.1021/acs.orglett.5c01375)
Supplement: Supplementary file 1 [file ol5c01375_si_001.pdf]

Supporting Material

Synthesis of Pandaroside D from *Pandaros acanthifolium* via Construction of a Rare Enone System in the Steroid D Ring

K. Michalak, R. Bujok, P. Cmoch and J. Mlynarski

Institute of Organic Chemistry, Polish Academy of Sciences, Warsaw, Poland

A. General Information 1

B. Experimental Procedures 1

Glycosylation of steroid (16) with trichloroacetimidate (18).. 8

Glycosylation of steroid 16 with bromide 21 10

7. References 12

C. NMR Spectra 13

A. General Information

All starting materials and reagents were purchased from commercial sources and used as received. Tetrahydrofuran was freshly distilled from Na/K alloy and dichloromethane was freshly distilled from $LiAlH_4$ prior to use. Analytical TLC was performed with Silica gel 60 F254 aluminum plates (Merck) with visualization by UV light and by treatment with aqueous cerium(IV) sulphate solution with molybdic and sulfuric acid followed by heating. Column chromatography was performed by using silica gel from Merck (Silica gel 60, 40-63 μm). Optical rotations were measured at RT with a digital polarimeter. $CDCl_3$ and CD_3OD were used as NMR solvents. ^{1}H NMR spectra were recorded with 600, 500 and 400 MHz and referenced relative to tetramethylsilane or the solvent residual signal: CDCl3 (δ = 7.26 ppm) and CD_3OD (δ = 3.31 ppm). Data are reported as follows: chemical shift in parts per million (ppm), multiplicity (bs = broad singlet, s = singlet, d = doublet, t = triplet, dd = doublet of doublets, m = multiplet), coupling constants in hertz (Hz). ^{13}C NMR spectra were measured at 150, 125 and 120 MHz with complete proton decoupling. Chemical shifts were reported in ppm from the residual solvent as an internal standard: CDCl3 (δ = 77.2 ppm) and CD_3OD (δ = 49.0 ppm). Structural assignments were made with additional information from gCOSY, gHSQC, and gHMBC experiments. High-resolution mass spectra were recorded using SYNAPT G2-S HDMS (Waters Inc.). Mass spectrometer equipped with an atmospheric-pressure chemical ionization (APCI) ion source and electrospray ionization (ESI) ion source and quadrupole-Time-of-Flight (q-TOF) mass analyzer. Specific rotations were recorded on a JASCO P-2000-Na polarymeter at 20–25 °C.

B. Experimental Procedures

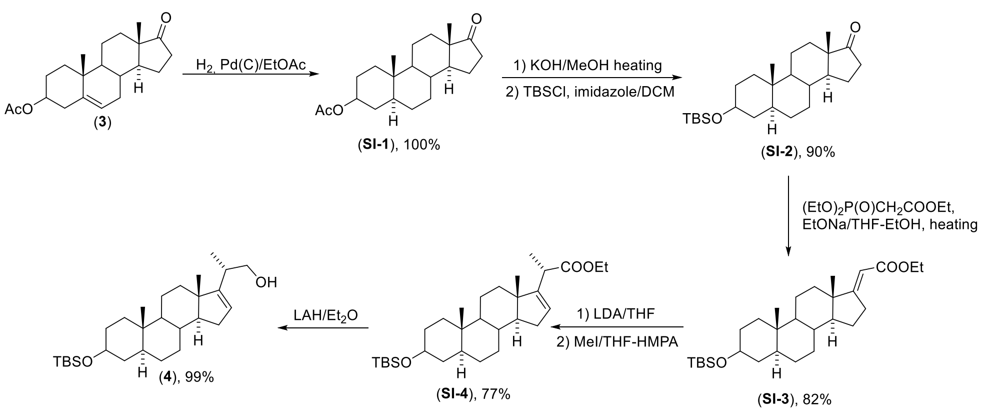

Compound (**SI-1**) was prepared under standard conditions.[1] Ethyl acetate was used as solvent instead of ethanol. Alcohol (**4**) was synthesised from (**SI-1**) according to Ibuka's procedure.[2]

***tert*-Butyl(((*3S*,*5S*,*10S*,*13S*,*14S*)-17-((*S*)-1-iodopropan-2-yl)-10,13-dimethyl-2,3,4,5,6,7,8,9,10,11,12,13,14,15-tetradecahydro-1*H*-cyclopenta[a]phenanthren-3-yl)oxy)-dimethylsilane (5)**

A solution of alcohol (**4**) (4.24 g, 9.5 mmol) in pyridine (20 mL) was cooled to 0 °C, TsCl (4.0 g, 21 mmol) was added and the mixture was stirred at the temperature for 3 h. Cooling bath was removed and reaction mixture was stirred at room temperature for 16 h. Reaction mixture was cooled to 0 °C, water (2 mL) was added and stirring was continued for 10 min. Water and mixture hexanes/ethyl acetate (6:4, 200 mL) were added, organic layer was washed with 1M HCl (100 mL), water (100 mL), brine, dried and evaporated.

To the crude product (5.78 g) acetone (42 mL), NaI (5.6 g, 37 mmol) and DIPEA (0.08 mL) were added and mixture was heated at 65 °C (oil bath) for 23 h. Mixture was evaporated to dryness and partitioned between hexanes (100 mL) and water (100 mL). Organic layer was dried and evaporated. Crude product was purified by column chromatography on silica gel (3% EtOAc in hexanes) to give (**5**) (5.09 g, 96%) as white solid.

m.p. 108–110 °C (EtOH)

$[\alpha]_D^{22}$ = –0.62 (c = 1.0, $CHCl_3$)

^{1}H NMR (400 MHz, $CDCl_3$) δ 5.42 – 5.37 (m, 1H), 3.60 – 3.49 (m, 1H), 3.34 (dd, *J* = 9.6, 5.3 Hz, 1H), 3.17 (dd, *J* = 9.5, 8.6 Hz, 1H), 2.43 – 2.38 (m, 1H), 2.06 – 2.03 (m, 1H), 1.88 – 1.77 (m, 1H), 1.74 – 1.23 (m, 14H), 1.16 (d, *J* = 6.8 Hz, 3H), 1.12 – 1.02 (m, 1H), 1.02 – 0.86 (m, 2H), 0.88 (s, 9H), 0.83 (s, 3H), 0.79 (s, 3H), 0.75 – 0.63 (m, 1H), 0.05 (s, 6H).

^{13}C NMR (100 MHz, $CDCl_3$) δ 158.0, 122.9, 72.1, 57.1, 55.1, 47.2, 45.3, 38.7, 37.1, 35.8, 35.5, 35.0, 34.2, 32.0, 32.0, 31.1, 28.7, 26.0, 21.9, 21.1, 18.2, 16.5, 14.9, 12.4, –4.5.

HRMS(APCI) *m/z*: $[M - {}^tBuMe_2SiO]^+$ Calculated for $C_{22}H_{34}I$: 425.1705, Found: 425.1708

(*2R*)-2-((*3S*,*5S*,*10S*,*13S*,*14S*)-3-((*tert*-butyldimethylsilyl)oxy)-10,13-dimethyl-2,3,4,5,6,7,8,9,10,11,12,13,14,15-tetradecahydro-1*H*-cyclopenta[a]phenanthren-17-yl)-6-methylheptan-4-ol (7)

A solution of iodide (**5**) (4.72 g, 8.4 mmol) in hexanes (53 mL) and dry Et_2O (27 mL) was cooled to –78 °C and 1.7 M *t*-BuLi in pentane (11.0 mL, 2.2 eq.) was added dropwise under Ar. When the addition was completed, the mixture was stirred for 15 min. Bath was removed and the solution was stirred 1 h at room temperature. Then the mixture was cooled again to –78 °C, isovalerylaldehyde (1.82 mL, 16.9 mmol) was added and the solution was stirred at this temperature for 1 h. Aqueous $NaHCO_3$ (15 mL) was added, bath was removed, the mixture was poured into water and extracted with hexanes (100 mL). Organic layer was

washed with brine, dried and evaporated. Crude product was purified by column chromatography on silica gel (1.5% EtOAc in hexanes) to give (**7**) (3.3 g, 75%) as a white semi crystalline mass (mixture of diastereoisomers). This unseparable mixture of stereoisomers was submitted directly to the oxidation step to form compound **8**.

^{1}H NMR (400 MHz, $CDCl_3$, mixture of diastereoisomers) δ 5.41 – 5.34 (m, 0.4H), 5.26 (brs, 0.6H), 3.75 – 3.63(m, 1H), 3.58 – 3.49 (m, 1H), 2.42 – 2.27 (m, 1H), 2.06 – 1.98 (m, 1H), 1.84 – 1.17 (m, 30H), 1.14 –0.66(m, 19H), 0.04 (s, 6H).

^{13}C NMR (100 MHz, $CDCl_3$, mixture of diastereoisomers) δ 161.3, 160.3, 121.6, 121.0, 72.1, 68.7, 68.1, 57.5, 57.3, 55.2, 55.1, 47.5, 47.4, 47.4, 47.3, 45.3, 45.1, 44.7, 38.7, 37.1, 35.8, 35.8, 35.3, 34.3, 34.2, 32.0, 32.0, 32.0, 31.0, 31.0, 29.8, 29.1, 28.8, 26.0, 24.6, 24.6, 23.6, 23.4, 23.0, 22.3, 22.1, 21.8, 21.2, 21.1, 18.2, 16.7, 16.3, 12.4, 12.4, –4.6.

HRMS(ESI) *m/z*: $[M + Na]^+$ Calculated for $C_{33}H_{60}O_2SiNa$: 539.4260, Found: 539.4254.

(*2R*)-2-((*3S*,*5S*,*10S*,*13S*,*14S*)-3-((*tert*-butyldimethylsilyl)oxy)-10,13-dimethyl-2,3,4,5,6,7,8,9,10,11,12,13,14,15-tetradecahydro-1*H*-cyclopenta[a]phenanthren-17-yl)-6-methylheptan-4-one (8)

Alcohol (**7**) (3.11 g, 6.0 mmol) was dissolved in DCM (50 mL). Dess-Martin periodinate (3.83 g, 9.0 mmol) was added and the mixture was stirred at room temperature for 1 h. Then mixture was poured to aqueous Na_2SO_3 (20 mL) and extracted with hexanes (100 mL). The organic layer was washed with aqueous Na_2SO_3 (20 mL), brine, dried and evaporated to give (**8**) (3.08 g, 100%) as a white solid.

m.p. 100–101 °C (MeOH)

$[\alpha]_D^{22}$ = +7.73 (c = 1.1, $CHCl_3$)

^{1}H NMR (500 MHz, $CDCl_3$) δ 5.28 (s, 1H), 3.58 – 3.51 (m, 1H), 2.74 – 2.67 (m, 1H), 2.60 (dd, *J* = 16.2, 6.2 Hz, 1H), 2.39 (dd, *J* = 16.2, 8.1 Hz, 1H), 2.25 (d, *J* = 6.9 Hz, 2H), 2.16 – 2.08 (m, 1H), 2.03 – 1.97 (m, 1H), 1.78 – 1.21 (m, 15H), 1.11 – 1.03 (m, 1H), 0.99 (d, *J* = 6.8 Hz, 3H), 0.95 – 0.85 (m, 17H), 0.82 (s, 3H), 0.75 (s, 3H), 0.72 – 0.66 (m, 1H), 0.04 (s, 6H).

^{13}C NMR (125 MHz, $CDCl_3$) δ 210.1, 160.2, 121.2, 72.1, 57.3, 55.1, 52.5, 50.2, 47.4, 45.3, 38.7, 37.0, 35.7, 35.3, 34.2, 32.0, 32.0., 31.0, 28.7, 28.0, 26.0, 24.4, 22.6, 22.6, 21.7, 21.2, 18.3, 16.3, 12.4, –4.6.

HRMS(ESI) *m/z*: $[M + Na]^+$ Calculated for $C_{33}H_{58}O_2SiNa$: 537.4101, Found: 537.4104.

***tert*-butyl(((*3S*,*5S*,*10S*,*13S*,*14S*)-17-((*R*)-1-(2-isobutyl-1,3-dioxolan-2-yl)propan-2-yl)-**

10,13-dimethyl-2,3,4,5,6,7,8,9,10,11,12,13,14,15-tetradecahydro-1*H*-cyclopenta[a]-phenanthren-3-yl)oxy)dimethylsilane (9)

To a solution of ketone (**8**) (3.24 g, 6.3 mmol) in DCM (50 mL), fresh distilled ethylene glycol (100 mL), trimethyl orthoformate (7.5 mL) and *p*-TsOH (0.25 g) were added. The mixture was stirred for 24 h at room temperature and Et_3N (2 mL) was added. The mixture was poured into water (300 mL) and extracted with mixture DCM (50 mL) and hexanes (300 mL). The organic layer was washed with water (3×200 mL), brine, dried and evaporated to give mixture of desired acetal (**9**) and the acetal without TBS group. The crude product was dissolved in DCM (40 mL), imidazole (1.41 g, 20.7 mmol) and TBSCl (1.413 g, 9.4 mmol) were consecutively added. Resulting suspension was stirred for 16 h at room temperature and poured into diluted aqueous $NaHCO_3$ (300 mL). Water layer was washed with DCM (15 mL). The combined organic extracts were dried and evaporated. The product was purified by column chromatography on silica gel (2% EtOAc in hexanes with few drops of Et_3N) to give desired acetal (**9**) (3.44 g, 98%) as a white solid.

m.p. 113–114 °C (EtOH)

$[\alpha]_D^{23}$ = +8.36 (c = 1.0, $CHCl_3$)

^{1}H NMR (400 MHz, $CDCl_3$) δ 5.30 – 5.28 (m, 1H), 3.97 – 3.84 (m, 4H), 3.59 – 3.51 (m, 1H), 2.34 – 2.24 (m, 1H), 2.04 – 1.98 (m, 1H), 1.90 (dd, *J* = 14.5, 3.6 Hz, 1H), 1.83 – 1.23 (m, 22H), 1.07 (d, *J* = 6.8 Hz, 3H), 0.95 – 0.88 (m, 15H), 0.83 (s, 3H), 0.78 (s, 3H), 0.73 – 0.64 (m, 1H), 0.05 (s, 6H).

^{13}C NMR (100 MHz, $CDCl_3$) δ 162.3, 120.6, 112.2, 72.2, 64.3, 64.1, 57.4, 55.2, 47.5, 45.4, 45.3, 43.7, 38.7, 37.1, 35.8, 35.3, 34.2, 32.0, 32.0, 31.0, 28.8, 27.9, 25.9, 24.3, 24.1, 23.8, 22.7, 21.2, 18.2, 16.6, 12.4, –4.6.

Anal. Calcd for $C_{35}H_{62}O_3Si$: C, 75.21; H, 11.18; found: C, 75.11; H, 11.26.

(*3S*,*5S*,*10S*,*13S*,*14S*,*16R*,*17R*)-3-((*tert*-butyldimethylsilyl)oxy)-17-((*R)*-1-(2-*iso*butyl-1,3-dioxolan-2-yl)propan-2-yl)-10,13-dimethylhexadecahydro-1*H*-cyclopenta[a]phenanthren-16-ol (10)

$BH_3{\cdot}Me_2S$ in THF (2M, 8.5 mL, 17 mmol) was added dropwise to a solution of (**9**) (1.695 g, 3.0 mmol) in THF (66 mL) and Et_3N (1.15 mL) at –30 °C. After 30 min the cooling bath was removed and the mixture was allowed to warm to room temperature and stirred overnight. The mixture was cooled again to –30 °C, EtOH (23 mL), 3M NaOH (23 mL) and H_2O_2 (30%, 26.5 mL) were consecutively added with 5 min intervals. The mixture was allowed to slowly

warm to room temperature and after 20 h it was poured into water (75 mL). The product was extracted twice with EtOAc, combined organic extracts were washed with brine (15 mL) and dried. The solvent was evaporated and the residue was chromatographed on silica gel (2% EtOAc in hexanes with few drops of Et_3N) to give (**10**) as a white solid (1.350 g, 77%).

m.p. 141–143 °C (hexanes)

$[\alpha]_D^{24}$ = –3.34 (c = 1.0, $CHCl_3$)

^{1}H NMR (500 MHz, $CDCl_3$) δ 4.01 – 3.97 (m, 1H), 3.96 – 3.88 (m, 4H), 3.57 – 3.51 (m, 1H), 2.17 (dd, *J* = 14.9, 1.9 Hz, 1H), 1.94 – 1.91 (m, 2H), 1.80 – 1.73 (m, 2H), 1.70 – 1.56 (m, 5H), 1.54 – 1.20 (m, 15H), 1.10 – 1.02 (m, 5H), 0.94 – 0.92 (m, 6H), 0.87 (s, 9H), 0.78 (s, 3H), 0.69 – 0.62 (m, 4H), 0.04 (s, 6H).

^{13}C NMR (125 MHz, $CDCl_3$) δ 112.9, 77.2, 72.1, 66.9, 64.4, 64.2, 54.3, 53.4, 45.9, 45.0, 44.4, 42.2, 40.2, 38.7, 37.1, 37.1, 35.5, 34.8, 32.1, 31.9, 30.3, 28.7, 26.0, 24.2, 24.2, 24.0, 21.5, 20.9, 18.3, 13.4, 12.4, –4.6.

HRMS(ESI) m/z: $[M + Na]^+$ Calculated for $C_{35}H_{64}O_4SiNa$: 599.4472, Found: 599.4475.

(*3S*,*5S*,*10S*,*13S*,*14S*,*17R*)-3-((*tert*-butyldimethylsilyl)oxy)-17-((*R*)-1-(2-*iso*butyl-1,3-dioxolan-2-yl)propan-2-yl)-10,13-dimethylhexadecahydro-16*H*-cyclopenta[a]phenanthren-16-one (11)

Alcohol (**10**) (1.350 g, 2.2 mmol) was dissolved in DCM (50 mL). Dess-Martin periodinane (1.374 g, 3.2 mmol) was added and the mixture was stirred at room temperature for 1 h. Then mixture was poured to aq. Na_2SO_3 and extracted with hexanes (70 mL). The organic layer was washed with aq. Na_2SO_3, brine, dried and evaporated to give (**11**) (1.342 g, 100%) as a white solid.

m.p. 152–153 °C (EtOH)

$[\alpha]_D^{24}$ = –76.20 (c = 1.0, $CHCl_3$)

^{1}H NMR (400 MHz, $CDCl_3$) δ 3.94 – 3.82 (m, 4H), 3.58 – 3.50 (m, 1H), 2.15 (dd, *J* = 18.2, 7.4 Hz, 1H), 2.06 – 1.97 (m, 3H), 1.83 – 1.24 (m, 19H), 1.10 – 1.03 (m, 4H), 0.95 – 0.73 (m, 3H), 0.92 (d, *J* = 6.7 Hz, 6H), 0.87 (s, 9H), 0.81 (s, 3H), 0.81 (s, 3H), 0.03 (s, 6H).

^{13}C NMR (100 MHz, $CDCl_3$) δ 218.4, 112.5, 72.0, 69.2, 64.4, 64.1, 54.3, 50.7, 45.7, 44.9, 43.4, 41.8, 39.1, 38.9, 38.5, 36.9, 35.6, 34.2, 32.1, 31.8, 28.5, 26.6, 25.9, 24.1, 24.0, 23.9, 20.9, 20.6, 18.2, 14.0, 12.3, –4.6.

HRMS(ESI) *m/z*: $[M+H]^+$ Calculated for $C_{35}H_{63}O_4Si$: 575.4496, Found: 575.4495

(*3S*,*5S*,*10S*,*13S*,*15R*,*17R*)-3-((*tert*-butyldimethylsilyl)oxy)-15-hydroxy-17-((*R*)-1-(2-*iso*butyl-1,3-dioxolan-2-yl)propan-2-yl)-10,13-dimethylhexadecahydro-16*H*-cyclopenta[a]phenanthren-16-one (12)

To a solution of ketone (**11**) (1.248 g, 2.17 mmol) in dry THF (25 mL) at –78 °C 1M NaHMDS in THF (3.25 mL, 1.5 eq.) was added dropwise and the mixture was stirred for 40 min under Ar. Then crystalline Davis reagent (0.848 g, 3.24 mmol) was added and the reaction mixture was stirred for 1 h. The reaction was quenched with aqueous $NaHCO_3$ (12 mL) and bath was removed. The mixture was allowed to warm to room temperature, diluted with water and extracted with EtOAc (100 mL). Organic layer was washed with brine, dried and evaporated. The product was purified by column chromatography on silica gel (10% EtOAc in hexanes with few drops of Et_3N) to give **12** (1.021 g) as a white semi crystalline mass. This product without any additional purification was submitted to the next step.

The configuration of the C-15 stereocenter was not determined. Our earlier works suggested that 15*R*-isomer should be formed predominantly, but it was not confirmed. It should be stressed that in next step the hydroxyl group was oxidise to ketone (or rather corresponding enol), so the configuration of the C-15 stereocenter in compound **12** does not matter from the view of synthesis of Pandaroside D.

^{1}H NMR (400 MHz, $CDCl_3$) δ 3.94 – 3.84 (m, 4H), 3.69 (d, *J* = 8.0 Hz, 1H), 3.58 – 3.50 (m, 1H), 2.09 – 2.05 (m, 2H), 2.02 – 1.95 (m, 2H), 1.81 – 1.09 (m, 24H), 0.92 – 0.83 (m, 22H), 0.03 (s, 6H).

(*3S*,*5S*,*10S*,*13R*,*17R*)-3-((*tert*-butyldimethylsilyl)oxy)-15-hydroxy-17-((*R*)-1-(2-*iso*butyl-1,3-dioxolan-2-yl)propan-2-yl)-10,13-dimethyl-1,2,3,4,5,6,7,8,9,10,11,12,13,17-tetradecahydro-16*H*-cyclopenta[a]phenanthren-16-one (13)

To a solution of hydroxyketone (**12**) (1.021 g) in DCM (20 mL) Dess-Martin periodinane (0.92 g, 2.17 mmol) was added and the mixture was stirred at room temperature for 0.5 h. Then mixture was poured into aqueous $Na_2S_2O_3$ (15 mL) and extracted with EtOAc (50 mL). The organic layer was washed with aqueous $Na_2S_2O_3$ (15 mL), water (15 mL), brine, dried and evaporated. Residue was purified by column chromatography on silica gel (8% EtOAc in hexanes with few drops of Et_3N) to give (**13**) (0.769 g, 60%) as a colorless oil.

$[\alpha]_D^{24}$ = +39.82 (c = 1.0, $CHCl_3$)

^{1}H NMR (400 MHz, $CDCl_3$) δ 5.34 (s, 1H), 3.96 – 3.84 (m, 4H), 3.59 – 3.50 (m, 1H), 2.52 – 2.45 (m, 1H), 2.34 – 2.22 (m, 2H), 2.04 – 1.91 (m, 3H), 1.89 – 1.11 (m, 27H), 0.98 – 0.87 (m, 15H), 0.04 (s, 6H).

^{13}C NMR (100 MHz, $CDCl_3$) δ 203.5, 151.5, 145.2, 112.4, 71.8, 64.5, 64.3, 61.8, 53.8, 46.0, 44.2, 43.3, 41.3, 40.3, 38.5, 37.1, 36.4, 36.2, 31.8, 30.0, 28.5, 27.2, 25.9, 24.0, 21.8, 20.8, 20.8, 18.2, 12.3, –4.6.

HRMS(ESI) *m/z*: $[M+H]^+$ Calculated for $C_{35}H_{61}O_5Si$: 589.4288, Found: 589.4292

(*3S*,*5S*,*10S*,*13S*,*14R*)-3-((*tert*-butyldimethylsilyl)oxy)-16-hydroxy-17-((*R*)-1-(2-isobutyl-1,3-dioxolan-2-yl)propan-2-yl)-10,13-dimethyl-1,2,3,4,5,6,7,8,9,10,11,12,13,14-tetradecahydro-15*H*-cyclopenta[a]phenanthren-15-one (14)

A solution of (**13**) (0.769 g, 1.30 mmol) and KOH (0.140 g) in THF (2.3 mL) and MeOH (6.7 mL) was stirred at room temperature for 48 h. The mixture was diluted with hexane/EtOAc (2:3, 100 mL) and poured into water. Organic layer was washed with water (30 mL), brine, dried and evaporated. Residue was purified by column chromatography on silica gel (8% EtOAc in hexanes) to give (**14**) (0.606 g, 79%) as a white foam.

$[\alpha]_D^{24}$ = +73.65 (c = 1.0, $CHCl_3$)

^{1}H NMR (400 MHz, $CDCl_3$) δ 5.57 (s, 1H), 3.98 – 3.86 (m, 4H), 3.54 – 3.47 (m, 1H), 2.50 – 2.41 (m, 1H), 2.19 – 2.12(m, 1H), 2.06 – 1.93 (m, 3H), 1.89 – 1.01 (m, 28H), 0.98 – 0.87 (m, 12H), 0.74 (s, 3H), 0.02 (s, 6H).

^{13}C NMR (100 MHz, $CDCl_3$) δ 204.4, 155.2, 148.0, 111.8, 72.0, 64.3, 64.2, 54.7, 45.3, 44.3, 44.3, 43.0, 40.9, 38.5, 36.9, 36.3, 33.8, 31.8, 31.5, 30.0, 28.9, 26.8, 25.9, 25.4, 24.1, 24.0, 18.8, 18.5, 18.2, 10.8, –4.6, –4.5.

HRMS(ESI)*m/z*: $[M+Na]^+$ Calculated for $C_{35}H_{60}O_5SiNa$: 611.4108, Found: 611.4104.

(*3S*,*5S*,*10S*,*13S*,*14R*)-3-((*tert*-butyldimethylsilyl)oxy)-17-((*R*)-1-(2-*iso*butyl-1,3-dioxolan-2-yl)propan-2-yl)-10,13-dimethyl-15-oxo-2,3,4,5,6,7,8,9,10,11,12,13,14,15-tetradeca-hydro-1*H*-cyclopenta[a]phenanthren-16-yl acetate (15)

To a solution of compound (**14**) (0.569 g, 0.96 mmol) in DCM (20 mL), Et_3N (0.51 mL, 3.84 mmol), DMAP (0.020 g) and Ac_2O (0.180 mL, 1.91 mmol) were added subsequently. Resulting solution was stirred at room temperature for 1 h and MeOH (1 mL) was added. Stirring was continued for 5 min, diluted with hexane/EtOAc (2:3, 60 mL) and poured into water. Organic layer was washed with water (30 mL), brine, dried and evaporated. Residue was purified by column chromatography on silica gel (7% EtOAc in hexanes with few drops of Et_3N) to give (**15**) (0.568 g, 93%) as colorless oil.

$[\alpha]_D^{24}$ = +43.19 (c = 1.0, $CHCl_3$)

^{1}H NMR (400 MHz, CDCl$_3$) δ 3.97 – 3.84 (m, 4H), 3.55 – 3.45 (m, 1H), 2.62 – 2.50 (m, 1H), 2.29 – 2.10 (m, 4H), 1.96 (d, *J* = 4.5 Hz, 1H), 1.90 – 1.81 (m, 3H), 1.84 – 0.95 (m, 24H), 0.93 – 0.90 (m, 6H),0.85 (s, 9H), 0.74 (s, 3H), 0.01 (s, 6H).

^{13}C NMR (100 MHz, CDCl$_3$) δ 201.5, 171.4, 167.5, 146.0, 111.5, 72.0, 64.5, 64.2, 55.1, 45.5, 44.3, 44.1, 44.0, 41.7, 38.5, 36.7, 36.4, 33.8, 32.2, 31.5, 29.6, 28.9, 26.8, 25.9, 24.5, 24.0 (2C), 23.9, 20.4, 19.2, 18.9, 18.2, 10.9, –4.7.

HRMS (APCI) *m/z*: [M–Ac]$^+$ Calculated for $C_{35}H_{59}O_5Si$: 587.4132, Found: 587.4135.

(*3S*,*5S*,*8R*,*9S*,*10S*,*13S*,*14R*)-3-hydroxy-10,13-dimethyl-17-((*R*)-6-methyl-4-oxoheptan-2-yl)-15-oxo-2,3,4,5,6,7,8,9,10,11,12,13,14,15-tetradecahydro-1*H*-cyclopenta[a]phenanthren-16-yl acetate (16)

A solution of (**15**) (0.568 g, 0.90 mmol) and *p*-TsOH·H_2O (0.040 g) in acetone (15 mL) and H_2O (0.4 mL) was stirred at room temperature for 22 h. The mixture was concentrated to *ca*. 2 mL, diluted with hexane/EtOAc (2:3, 60 mL) and poured into aqueous $NaHCO_3$. Organic layer was washed with water (2×30 mL), brine, dried and evaporated. The crude product was purified by column chromatography on silica gel (35% EtOAc in hexanes) to give (**16**) (0.419 g, 99%) as colorless oil.

$[\alpha]_D^{24}$ = +48.20 (c = 1.0, $CHCl_3$)

^{1}H NMR (600 MHz, CDCl$_3$) δ 3.56 – 3.53 (m, 1H), 3.02 – 2.94 (m, 1H), 2.61 (dd, *J* = 17.5, 8.9 Hz, 1H), 2.52 (dd, *J* = 17.5, 4.5 Hz, 1H), 2.26 (s, 3H), 2.24 (d, *J* = 6.9 Hz, 2H), 2.19 – 2.04 (m, 2H), 1.96 (d, *J* = 4.5 Hz, 1H), 1.89 – 1.85 (m, 1H), 1.76 – 1.72 (m, 1H), 1.65 – 1.45 (m, 5H), 1.40 – 1.27 (m, 4H), 1.25 – 1.16 (m, 6H), 1.14 – 1.10 (m, 1H), 1.08 (d, *J*= 6.9 Hz, 3H), 1.02 – 0.95 (m, 1H), 0.92 – 0.82 (m, 7H), 0.74 (s, 3H)

^{13}C NMR (150 MHz, CDCl$_3$) δ 208.3, 201.5, 169.3, 167.6, 146.6, 71.1, 55.0, 52.3, 47.8, 44.3, 44.1, 44.0, 38.0, 36.8, 36.2, 33.9, 32.0, 30.9, 29.6, 28.8, 26.3, 24.6, 24.5, 22.6, 22.5, 20.4, 18.9, 18.1, 10.8.

HRMS(ESI)*m/z*: [M+Na]$^+$ Calculated for $C_{29}H_{44}O_5Na$: 495.3086, Found: 495.3090.

(*2S*,*3S*,*4S*,*5R*,*6R*)-2-(methoxycarbonyl)-6-(2,2,2-trichloro-1-iminoethoxy)tetrahydro-2*H*-pyran-3,4,5-triyl triacetate (**18**) was prepared from a commercial methyl 1,2,3,4-tetra-*O*-acetyl-β-D-glucuronate (**17**) according to literature method.[3]

Glycosylation of steroid (16) with trichloroacetimidate (18)

A solution of steroid (**16**) (0.063 g, 0.13 mmol) and saccharide (**18**) (0.130 g, 0.27 mmol) in

dry DCM (2.0 mL) was cooled under argon atmosphere to 0 °C and freshly prepared 0.1 M $BF_3 \cdot Et_2O$ in dry DCM (0.210 mL; 0.021 mmol) was added. The cooling bath was removed and the solution was stirred at room temperature for 2 d. The mixture without work-up was subject to column chromatography on silica gel (0–15% AcOEt in DCM). 0.030 g (30 %) of glycosylation product (**19**) (colourless oil) and 0.020 g (30%) of steroid acetate (**20**) (colourless oil) were obtained.

(*2R*,*3R*,*4S*,*5S*,*6S*)-2-(((*3S*,*5S*,*8R*,*9S*,*10S*,*13S*,*14R*)-16-acetoxy-10,13-dimethyl-17-((*R*)-6-methyl-4-oxoheptan-2-yl)-15-oxo-2,3,4,5,6,7,8,9,10,11,12,13,14,15-tetradecahydro-1*H*-cyclopenta[a]phenanthren-3-yl)oxy)-6-(methoxycarbonyl)tetrahydro-2*H*-pyran-3,4,5-triyl triacetate (19)

$[\alpha]_D^{23}$ = + 22.82 (c = 0.80, CH_2Cl_2)

^{1}H NMR (600 MHz, $CDCl_3$) δ 5.23 – 5.17 (m, 2H),4.94 – 4.92 (m, 1H),4.60 (d, *J* = 7.9 Hz, 1H; *β-anomer*), 3.99 (d, *J* = 9.5 Hz, 1H), 3.73 (s, 3H), 3.54– 3.48(m, 1H),3.00 – 2.94 (m, 1H),2.61 (dd, *J* = 17.5, 9.1 Hz, 1H),2.51 (dd, *J* = 17.5, 4.4 Hz, 1H), 2.27 (s, 3H), 2.23 (d, *J* = 7.0 Hz, 2H), 2.20 – 2.06 (m, 2H), 2.01 (s, 3H), 1.99 (s, 3H), 1.98 (s, 3H), 1.96(d, *J* = 4.7 Hz, 1H), 1.88 – 1.78 (m, 2H), 1.66– 1.58 (m, 2H), 1.56 – 1.42 (m, 4H), 1.32 – 1.28 (m, 3H),1.26 – 1.22 (m, 1H), 1.19 (s, 3H),1.16 – 1.04 (m, 5H),1.00 – 0.95 (m, 1H),0.90 – 0.88 (m, 6H), 0.85 – 0.80 (m, 1H), 0.72 (s, 3H)

^{13}C NMR (125 MHz, $CDCl_3$) δ 208.3, 201.4, 170.2, 169.4, 169.3, 169.2, 167.5, 167.3, 146.6, 99.5, 79.6, 72.5, 72.2, 71.4, 69.5, 55.0, 52.8, 52.3, 47.8, 44.2, 44.0, 43.8, 36.8, 36.1, 34.3, 33.8, 32.0, 29.5, 28.9, 28.7, 26.3, 24.6, 24.4, 22.5, 22.5, 20.7, 20.6, 20.5, 20.4, 18.9, 18.2, 10.8.

HRMS(ESI)*m/z*: $[M+Na]^+$ Calculated for $C_{42}H_{60}O_{14}Na$: 811.3881, Found: 811.3876.

(*3S*,*5S*,*8R*,*9S*,*10S*,*13S*,*14R*)-10,13-dimethyl-17-((*R*)-6-methyl-4-oxoheptan-2-yl)-15-oxo-2,3,4,5,6,7,8,9,10,11,12,13,14,15-tetradecahydro-1*H*-cyclopenta[a]phenanthrene-3,16-diyl diacetate (20)

$[\alpha]_D^{23}$ = +43.13 (c = 1.05, CH_2Cl_2)

^{1}H NMR (500 MHz, $CDCl_3$) δ 4.68 – 4.61 (m, 1H), 3.04 – 2.96 (m, 1H),2.61 (dd, *J* = 17.5, 9.1 Hz, 1H),2.51 (dd, *J* = 17.5, 4.4 Hz, 1H), 2.28 (s, 3H, Ac), 2.24 (d, *J* = 7.0 Hz, 1H), 2.21 – 2.08 (m, 2H), 2.00 (s, 3H, Ac), 1.97 (d, *J* = 4.6 Hz, 1H), 1.90 – 1.83 (m, 1H), 1.81 – 1.76 (m, 1H), 1.66– 1.41 (m, 7H), 1.34 – 1.17 (m, 8H),1.10 (d, *J* = 7.0 Hz, 3H), 1.05 – 0.95 (m,

2H),0.92 – 0.83 (m, 7H), 0.78 (s, 3H)

^{13}C NMR (125 MHz, $CDCl_3$) δ 208.3, 201.3, 170.5, 169.1, 167.5, 146.6, 73.5, 55.0, 52.4, 47.8, 44.2, 44.0, 43.8, 36.8, 35.9, 33.8, 32.1, 29.5, 28.8, 27.0, 26.3, 24.6, 24.4, 22.5, 22.4, 21.4, 20.5, 18.9, 18.2, 10.7.

HRMS(ESI)*m/z*: $[M+Na]^+$ Calculated for $C_{31}H_{46}O_6Na$: 537.3192, Found: 537.3195.

Signals assigned to protons in steroid (**16**) and glycosylation product (**19**) based on ^{1}H, 2D-COSY, ^{1}H-^{13}C HSQC, ^{1}H-^{13}C HMBC NMR spectra	
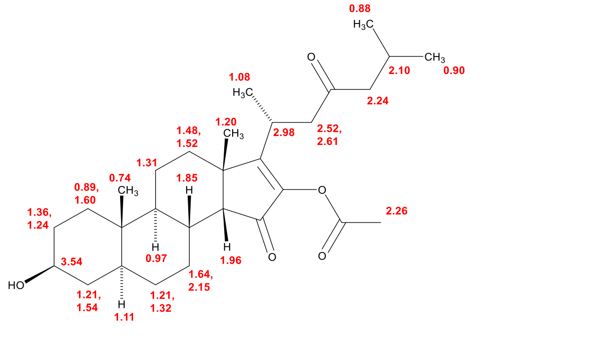	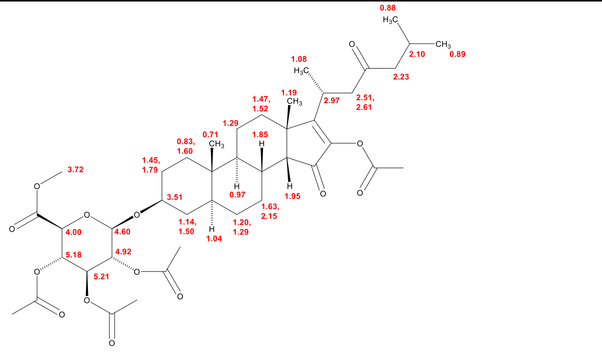
Signals assigned to carbons in steroid (**16**) and glycosylation product (**19**) based on^{13}C, ^{1}H-^{13}C HSQC, ^{1}H-^{13}C HMBC NMR spectra	
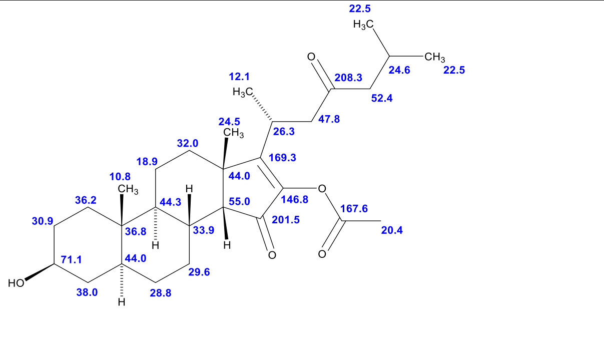	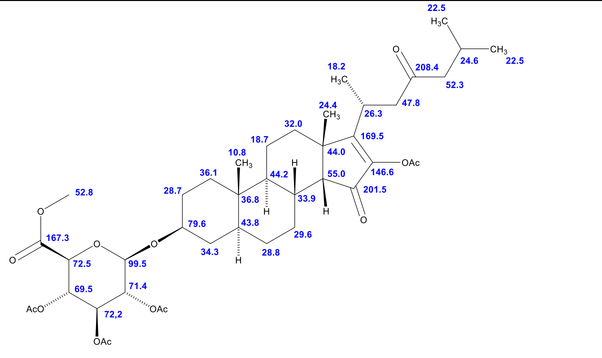

Glycosylation of steroid 16 with bromide 21

To a solution of the steroid **16** (0.046 g, 0.097 mmol) and bromide **21** (0.101 g, 0.25 mmol) in toluene (2 mL) at room temperature Ag_2CO_3 (0.142 g, 0.51 mmol) was added, the flask was protected from light and the mixture was stirred for 1 d. The mixture without work-up was subject to column chromatography on silica gel (0–15% AcOEt in DCM). 0.053 g (69 %) of glycosylation product **19** was obtained.

Methyl (*2S*,*3S*,*4S*,*5R*,*6R*)-3,4,5-trihydroxy-6-(((*3S*,*5S*,*8R*,*9S*,*10S*,*13S*,*14R*)-16-hydroxy-10,13-dimethyl-17-((*R*)-6-methyl-4-oxoheptan-2-yl)-15-oxo-

2,3,4,5,6,7,8,9,10,11,12,13,14,15-tetradecahydro-1*H*-cyclopenta[a]phenanthren-3-yl)oxy)tetrahydro-2*H*-pyran-2-carboxylate (methyl ester of pandaroside D) (2)

A freshly prepared MeONa solution in MeOH from sodium (0.0057 g) and MeOH (5 mL) was added to solid compound **19** (0.080 g, 0.10 mmol) and the mixture was stirred at room temperature for 20 min. The mixture without work-up was subject to column chromatography on silica gel (50 % AcOEt in DCM, then pure AcOEt). 0.044 g (71%) of pandaroside D methyl ester was obtained.

$[\alpha]_D^{20}$ = +35.0 (c = 0.1, MeOH), lit.$[\alpha]_D^{20}$ = +35.0 (c = 0.1, MeOH)[4]

^{1}H NMR (500 MHz, CD_3OD) δ 4.44 (d, *J* = 7.8 Hz, 1H; β-anomer), 3.83 (d, *J* = 9.8 Hz, 1H), 3.76 (s, 3H), 3.64 – 3.56 (m, 1H), 3.53 – 3.49 (m, 1H),3.38 – 3.36 (m, 1H), 3.19 – 3.15 (m, 1H), 2.89 – 2.77 (m, 3H), 2.31 (d, *J* = 6.9 Hz, 2H), 2.20 – 2.05 (m, 3H), 1.97 – 1.91 (m, 1H), 1.85– 1.78 (m, 2H), 1.72– 1.65 (m, 3H), 1.55 – 1.25 (m, 8H), 1.20 – 1.10 (m, 6H),1.08 – 1.02 (m, 1H), 0.91 – 0.88 (m, 6H), 0.85 – 0.80 (m, 1H), 0.72 (s, 3H).

^{13}C NMR (125 MHz, CD_3OD) δ 212.3, 206.0, 171.3, 154.6, 151.3, 102.9, 79.9, 77.3, 76.7, 74.7, 73.1,56.1, 53.3, 52.8, 48.1, 45.8, 45.6, 43.6, 38.2, 37.4, 35.3, 35.1, 32.6, 31.3, 30.3, 30.0, 28.0, 25.9, 25.7, 22.9, 22.8, 19.9, 18.0, 11.1.

HRMS(ESI)*m/z*: $[M+H]^+$ Calculated for $C_{34}H_{53}O_{10}$: 621.3639, Found: 621.3634.

Comparison of ^{1}H and ^{13}C NMR data of natural and synthetic sample of methyl ester of pandaroside D (2). We compared only the signals listed in the original publications; the complete set of ^{1}H and ^{13}C NMR signals is provided in the analytical description of compound 2, above.

**^{1}H NMR (synthesised sample; this work)**	**^{1}H NMR (natural sample; ref. 9)**
4.44 (d, *J* = 7.8 Hz, 1H)	4.44 (d, *J* = 8.0 Hz, 1H)
3.83 (d, J = 9.8 Hz, 1H)	3.83 (d, J = 9.0 Hz, 1H)
3.76 (s, 3H)	3.76 (s, 3H)
3.53 – 3.49 (m, 1H)	3.50 (t, *J* = 9.0 Hz, 1H)
3.38 – 3.36 (m, 1H)	3.36 (t, *J* = 9.0 Hz, 1H)
3.19 – 3.15 (m, 1H)	3.16 (dd, t, *J* = 9.0, 8.0 Hz, 1H)
**^{13}C NMR (synthesised sample; this work)**	**^{13}C NMR (natural sample; ref. 9)**
171.3	171.5
102.9	103.0
77.3	77.4
76.7	76.8
74.7	74.9
73.1	73.3
52.8	52.9

(*2S*,*3S*,*4S*,*5R*,*6R*)-3,4,5-trihydroxy-6-(((*3S*,*5S*,*8R*,*9S*,*10S*,*13S*,*14R*)-16-hydroxy-10,13-dimethyl-17-((*R*)-6-methyl-4-oxoheptan-2-yl)-15-oxo-2,3,4,5,6,7,8,9,10,11,12,13,14,15-tetradecahydro-1*H*-cyclopenta[a]phenanthren-3-yl)oxy)tetrahydro-2*H*-pyran-2-carboxylic acid (pandaroside D) (1)

To a solution of pandaroside D methyl ester (2) (0.044 g, 0.071 mmol) in MeOH (3 mL) and H_2O (0.1 mL) KOH (0.008 g, 0.12 mmol) was added and the solution was stirred at room temperature for 1 d. The mixture was acidified with 1M HCl and without work-up it was subject to column chromatography on silica gel 30% MeOH in DCM). 0.042 g (98%) of pure pandaroside D was obtained.

$[\alpha]_D^{22}$ = +25.2 (c = 0.1, MeOH), lit.$[\alpha]_D^{20}$ = +45.1 (c = 0.1, MeOH)[4]

^{1}H NMR (500 MHz, CD_3OD) δ 4.43 (d, *J* = 7.8 Hz, 1H; β-anomer), 3.70 – 3.64 (m, 2H), 3.48 – 3.45 (m, 1H), 3.42 – 3.39 (m, 1H), 3.19 – 3.16 (m, 1H), 2.87 – 2.78 (m, 3H), 2.31 (d, *J* = 6.9 Hz, 2H), 2.16 – 2.12 (m, 1H), 2.10 – 2.06 (m, 1H), 1.97 – 1.83 (m, 3H), 1.71– 1.62 (m, 3H), 1.56 – 1.20 (m, 9H),1.17 – 1.12 (m, 5H),1.05 – 1.00 (m, 1H), 0.93 – 0.81 (m, 11H).

^{13}C NMR (125 MHz, CD_3OD) δ 212.2, 206.0, 177.4, 154.7, 151.3, 102.1, 79.4, 77.7, 76.2, 74.8, 73.6, 56.1, 53.3, 48.1, 45.9, 45.6, 43.7, 38.3, 37.6, 35.2, 35.1, 32.7, 31.3, 30.3, 28.9, 28.1, 25.8, 25.7, 22.9, 22.8, 20.0, 18.1, 11.3.

HRMS(ESI) *m/z*: $[M+Na]^+$ Calculated for $C_{33}H_{50}O_{10}Na$: 629.3302, Found: 629.3307.

7. References

[1] Kratena, N.; Biedermann, N.; Stojanovic, B.; Göschl, L.; Weil, M.; Enev, V. S.; Gmeiner, G.; Gärtner, P. Synthesis of a human long-term oxymetholone metabolite. *Steroids* **2019**,*150*, 108430.

[2] Ibuka, T.; Taga, T.; Shingu, T.; Saito, M.; Nishii, S.; Yamamoto, Y. New stereoselective synthesis of 20*S* and 20*R* steroidal side chains. Remarkable stereoselectivity differences between saturated and α,β-unsaturated steroidal esters. *J. Org. Chem.* **1988**,*53*, 3947–3952.

[3]Chen, Y.; Li, Y.; Yu, H.; Sugiarto, G.; Thon, V.; Hwang, J.; Ding, L.; Hie, L.; Chen, X. Tailor design and synthesis of heparan sulfate (HS) oligosaccharide analogs using sequential one-pot multienzyme (OPME) systems. *Angew. Chem. Int. Ed.***2013**,*52*, 11852–11856.

[4] Cachet, N.; Regalado, E.L., Genta-Jouve, G.; Mehiri, M.; Amade, P.; Thomas, O. P. Steroidal glycosides from the marine sponge *Pandaros acanthifolium*. *Steroids* **2009**, *74*, 746–750.

C. NMR Spectra

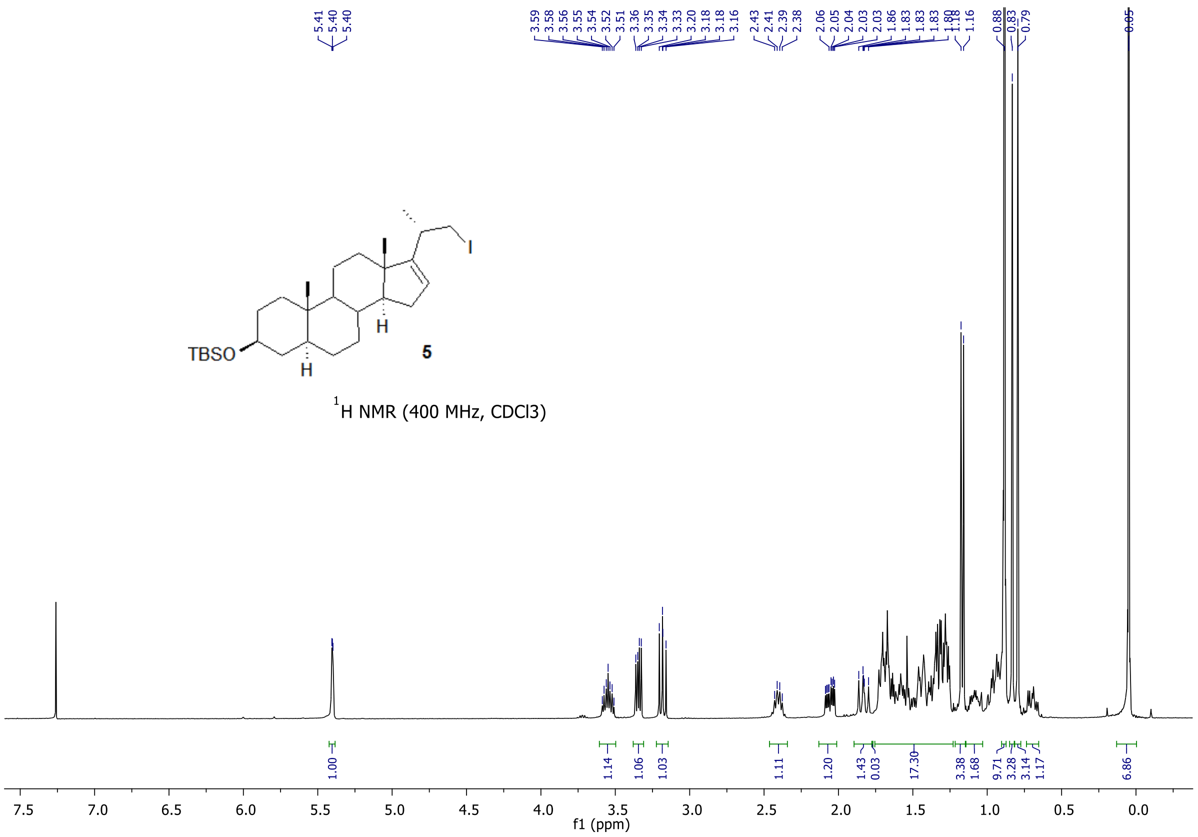

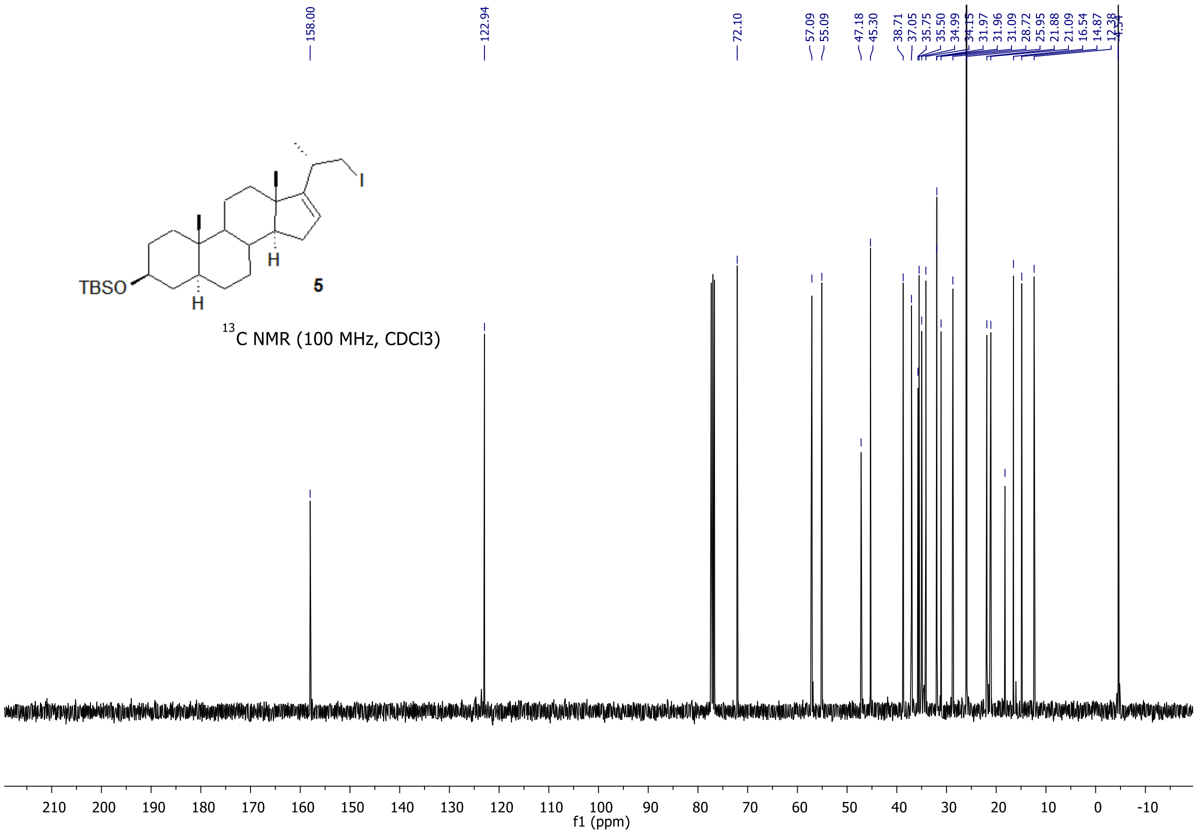

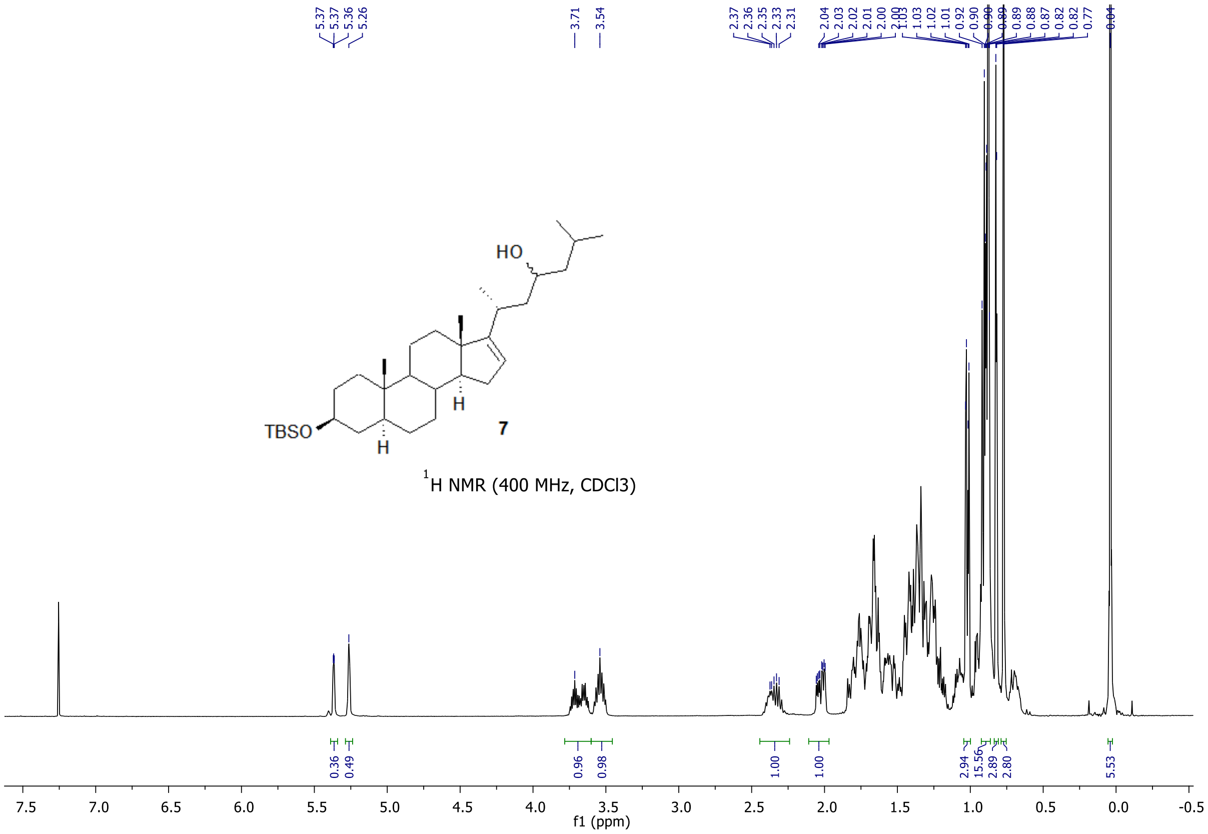
HO
TBSO
H
H
7
1H NMR (400 MHz, CDCl3)
5.37
5.37
5.36
5.26
3.71
3.54
2.37
2.36
2.35
2.33
2.31
2.04
2.03
2.02
2.01
2.00
1.03
1.02
1.01
0.92
0.90
0.89
0.88
0.87
0.82
0.82
0.77
0.36
0.49
0.96
0.98
1.00
1.00
2.94
15.56
2.89
2.80
5.53
7.5
7.0
6.5
6.0
5.5
5.0
4.5
4.0
3.5
3.0
2.5
2.0
1.5
1.0
0.5
0.0
-0.5
f1 (ppm)

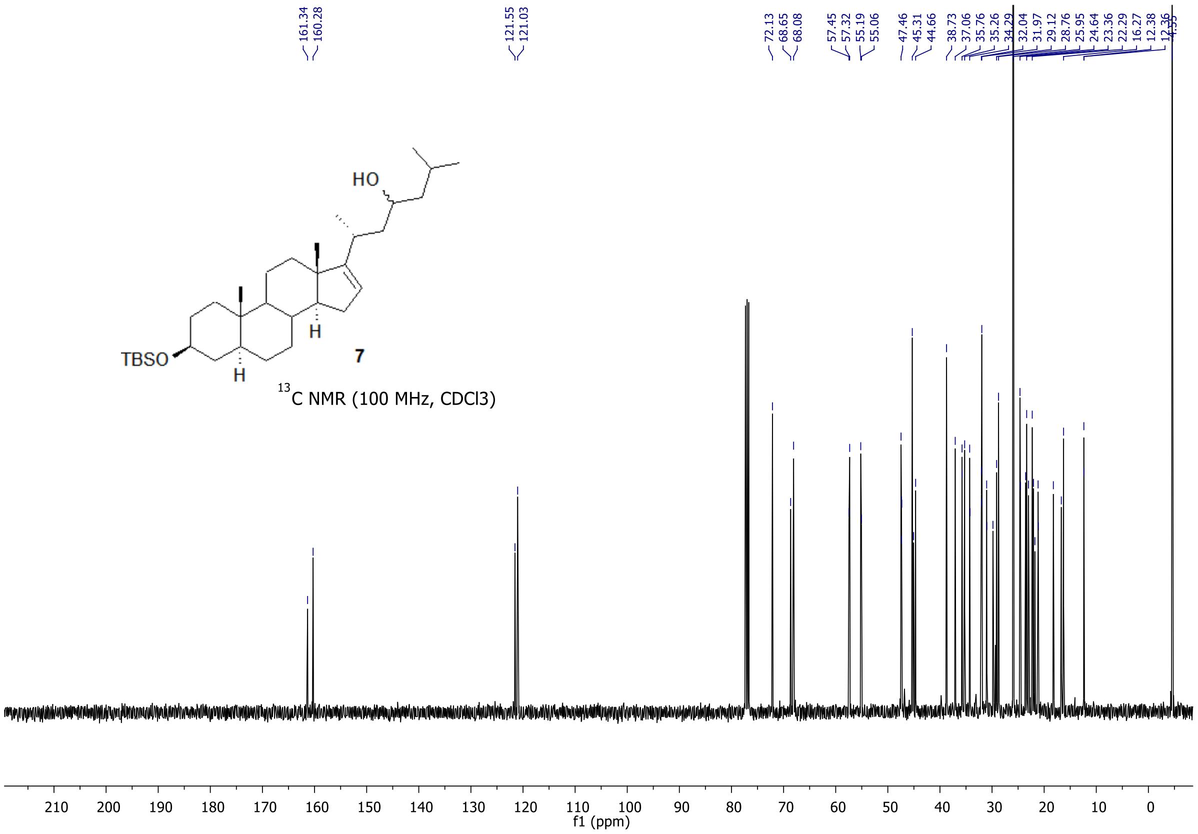
HO
TBSO
H
H
7
13C NMR (100 MHz, CDCl3)
161.34
160.28
121.55
121.03
72.13
68.65
68.08
57.45
57.32
55.19
55.06
47.46
45.31
44.66
38.73
37.06
35.76
35.26
34.29
32.04
31.97
29.12
28.76
25.95
24.64
23.36
22.29
16.27
12.38
12.36
210
200
190
180
170
160
150
140
130
120
110
100
90
80
70
60
50
40
30
20
10
0
f1 (ppm)

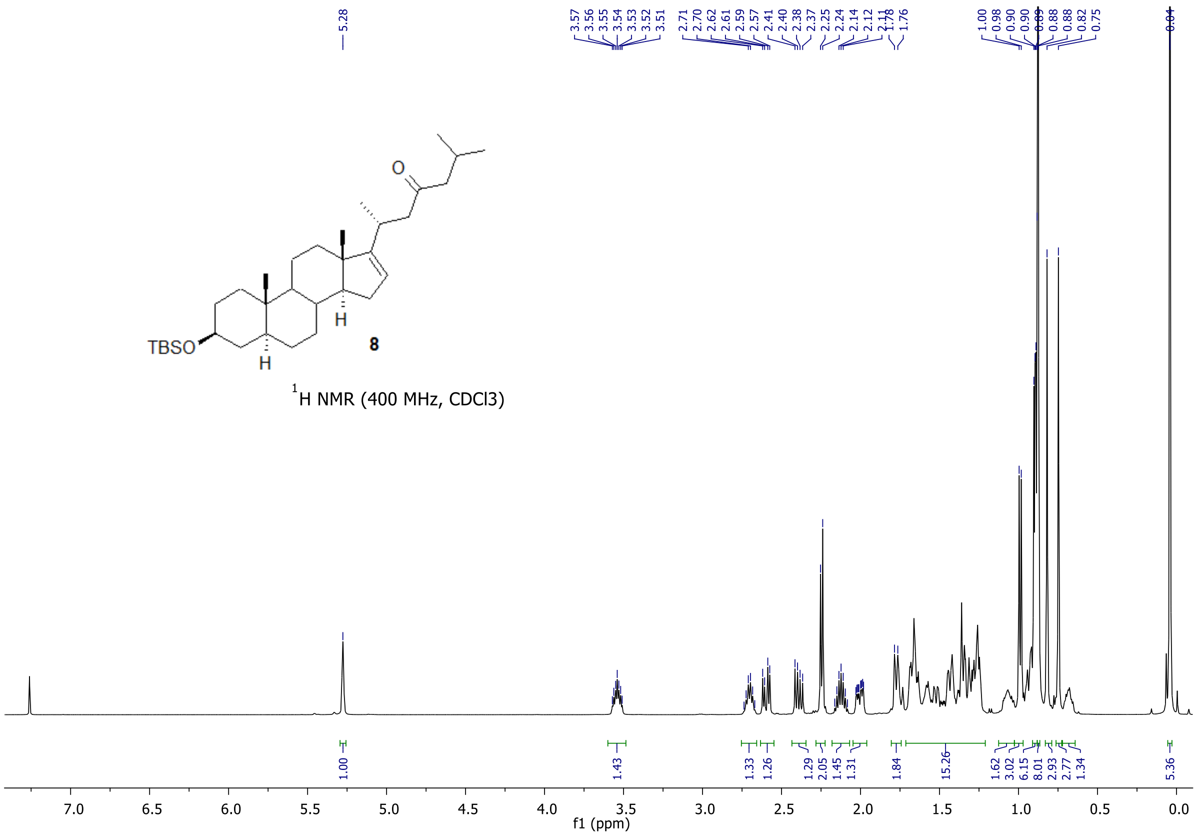
TBSO
H
H
O
8
1H NMR (400 MHz, CDCl3)
5.28
3.57 3.56 3.55 3.54 3.53 3.52 3.51
2.71 2.70 2.62 2.61 2.59 2.57 2.41 2.40 2.38 2.37 2.25 2.24 2.14 2.12 2.11 1.78 1.76
1.00 0.98 0.90 0.90 0.89 0.88 0.88 0.82 0.75
0.04
1.00 1.43 1.33 1.26 1.29 2.05 1.45 1.31 1.84 15.26 1.62 3.02 6.15 8.01 2.93 2.77 1.34 5.36
7.0 6.5 6.0 5.5 5.0 4.5 4.0 3.5 3.0 2.5 2.0 1.5 1.0 0.5 0.0
f1 (ppm)

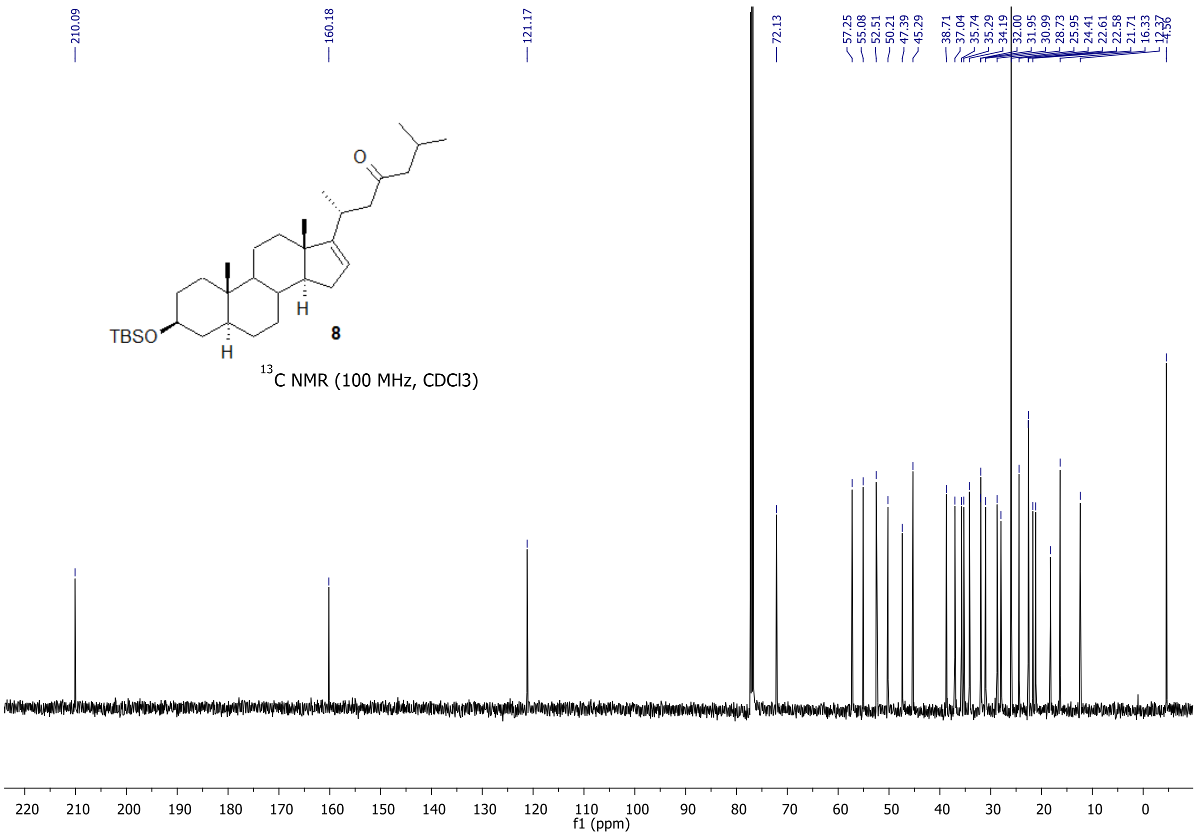
TBSO
H
H
O
8
13C NMR (100 MHz, CDCl3)
210.09
160.18
121.17
72.13
57.25 55.08 52.51 50.21 47.39 45.29
38.71 37.04 35.74 35.29 34.19 32.00 31.95 30.99 28.73 25.95 24.41 22.61 22.58 21.71 16.33 12.37 -4.56
220 210 200 190 180 170 160 150 140 130 120 110 100 90 80 70 60 50 40 30 20 10 0
f1 (ppm)

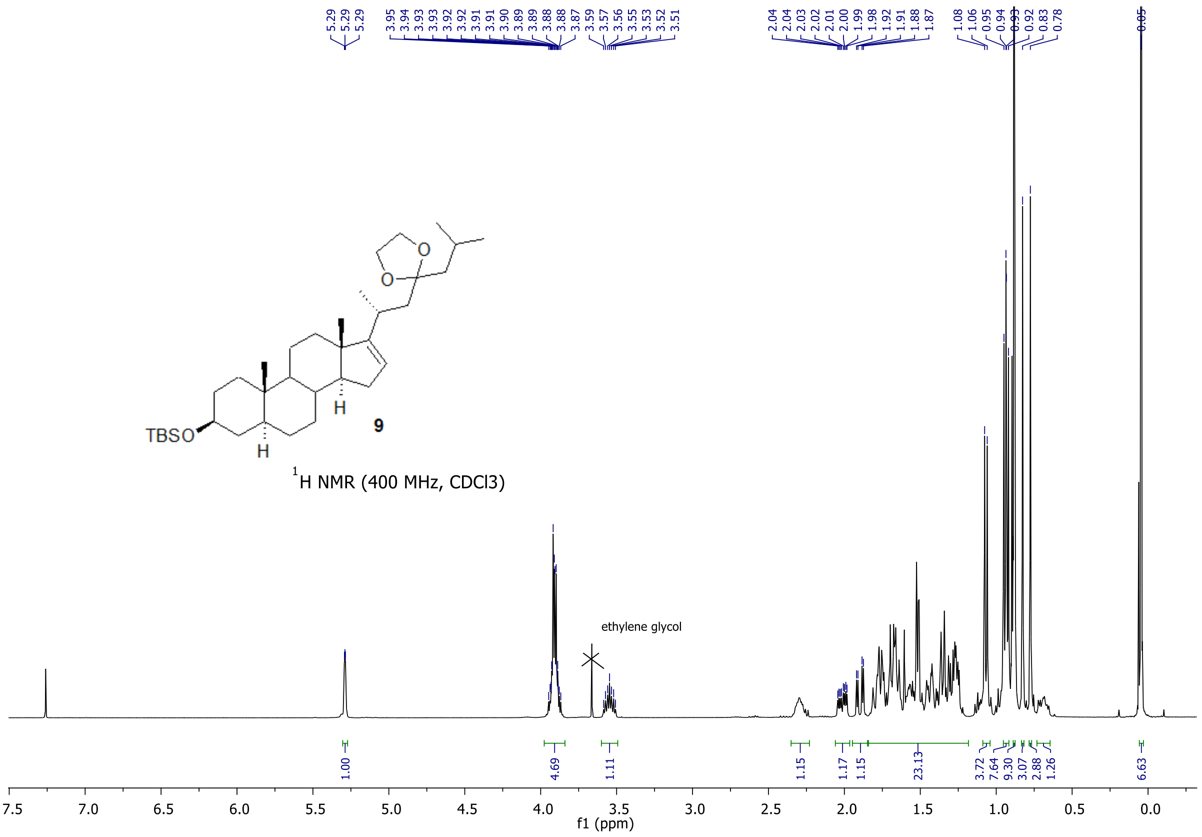

^{1}H NMR (400 MHz, CDCl3)

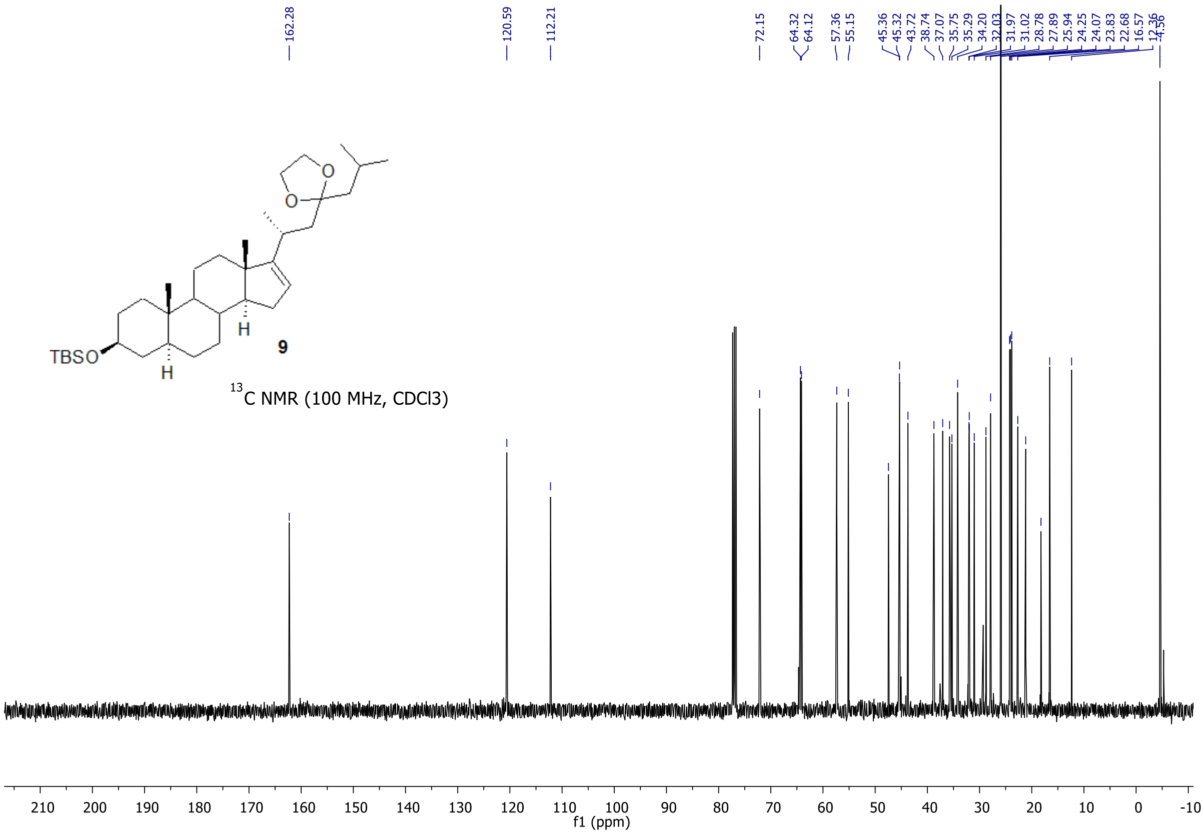

^{13}C NMR (100 MHz, CDCl3)

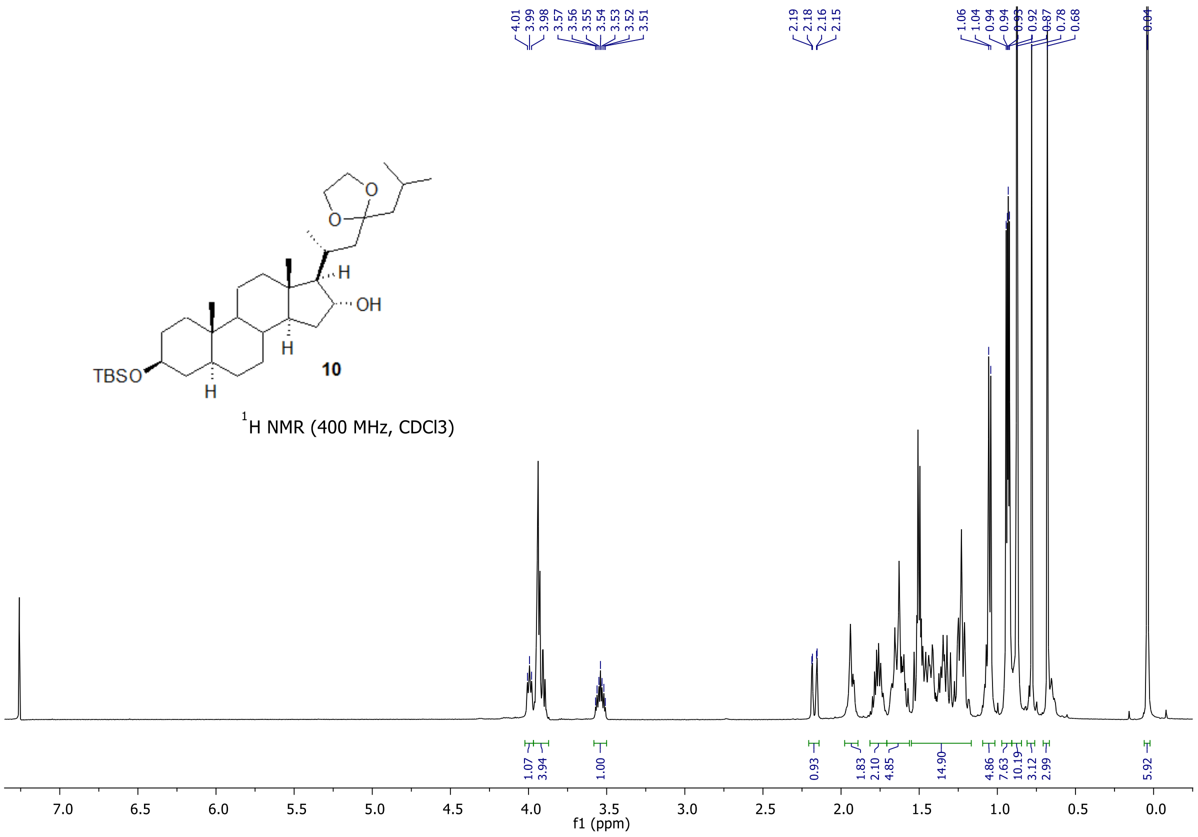
TBSO
OH
H
10
1H NMR (400 MHz, CDCl3)
f1 (ppm)

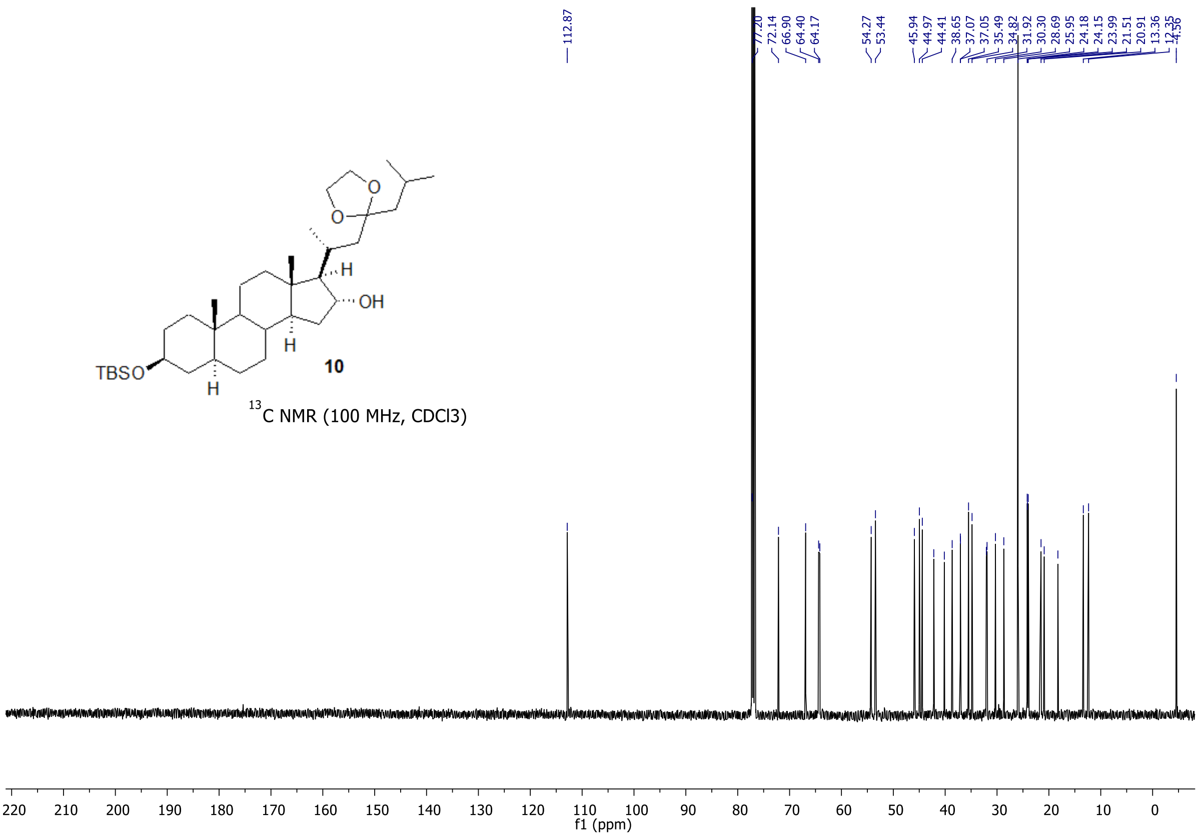
TBSO
OH
H
10
13C NMR (100 MHz, CDCl3)
f1 (ppm)

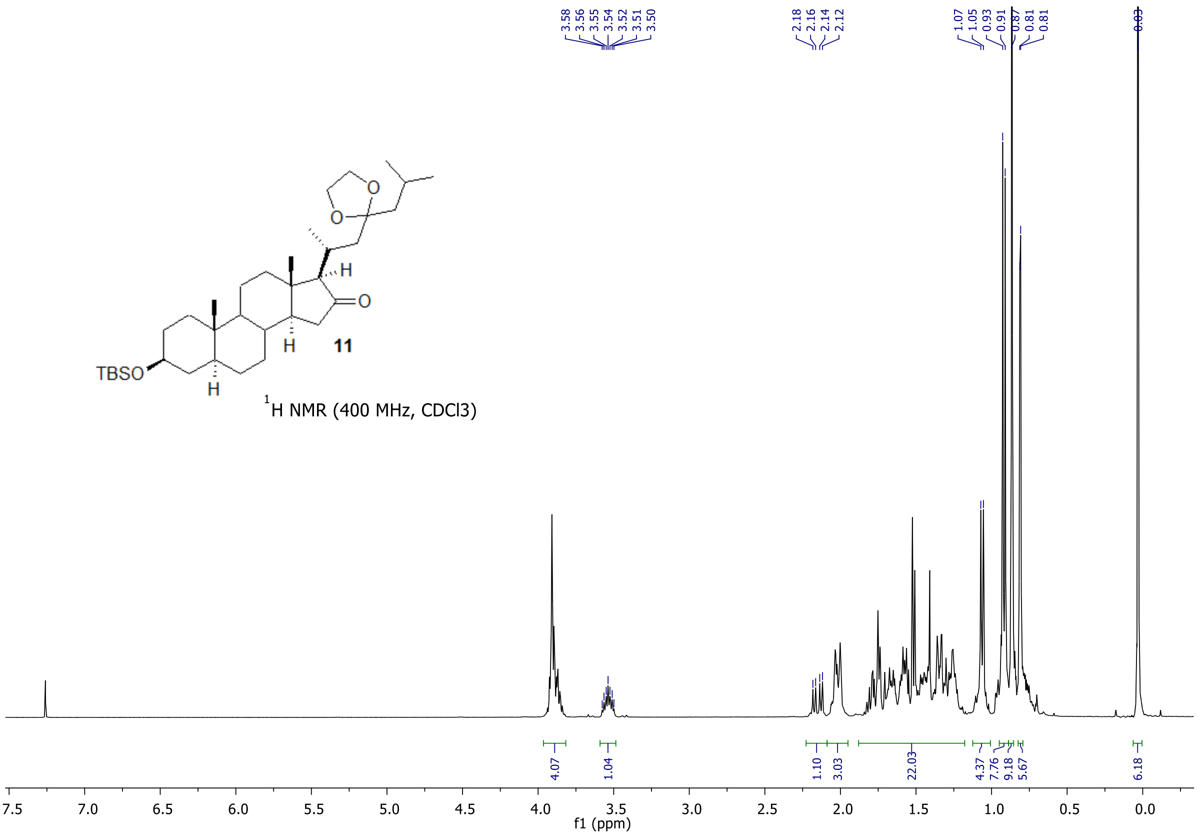

TBSO
H
H
H
O
O
O
11
1H NMR (400 MHz, CDCl3)
f1 (ppm)

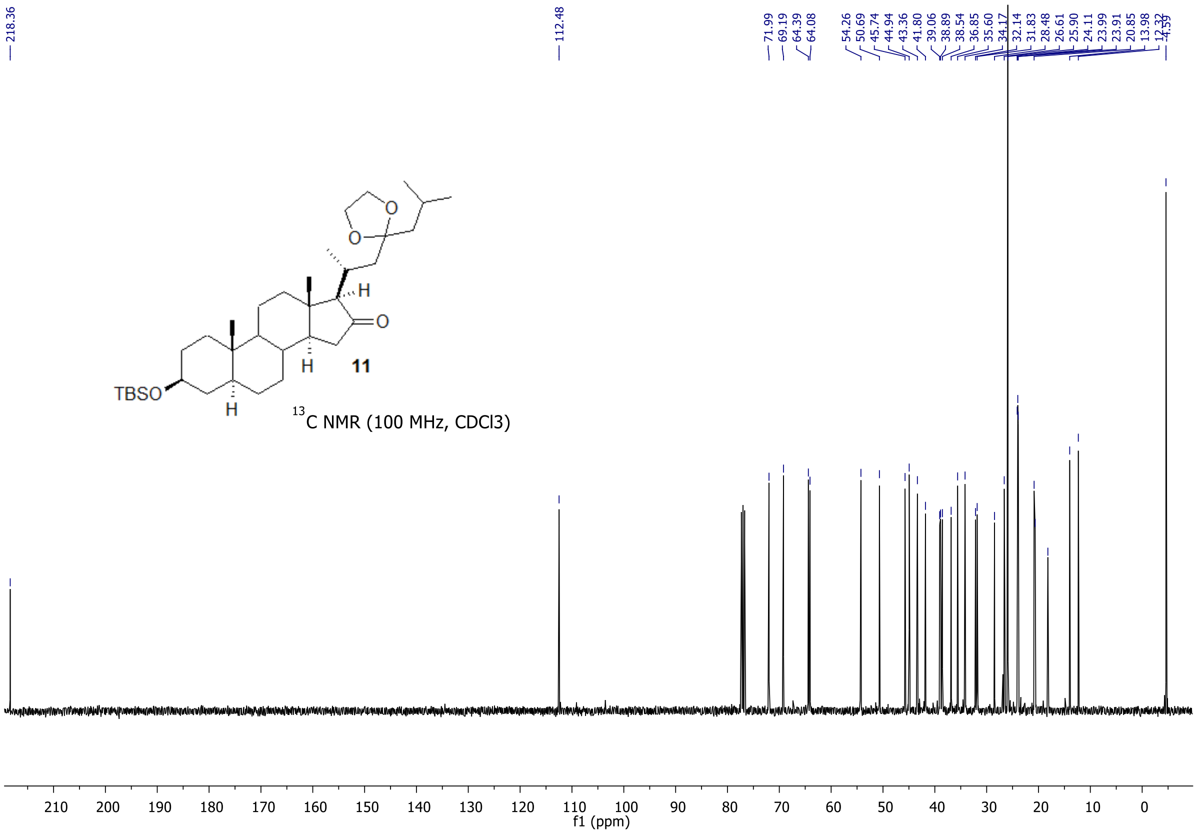

TBSO
H
H
H
O
O
O
11
13C NMR (100 MHz, CDCl3)
f1 (ppm)

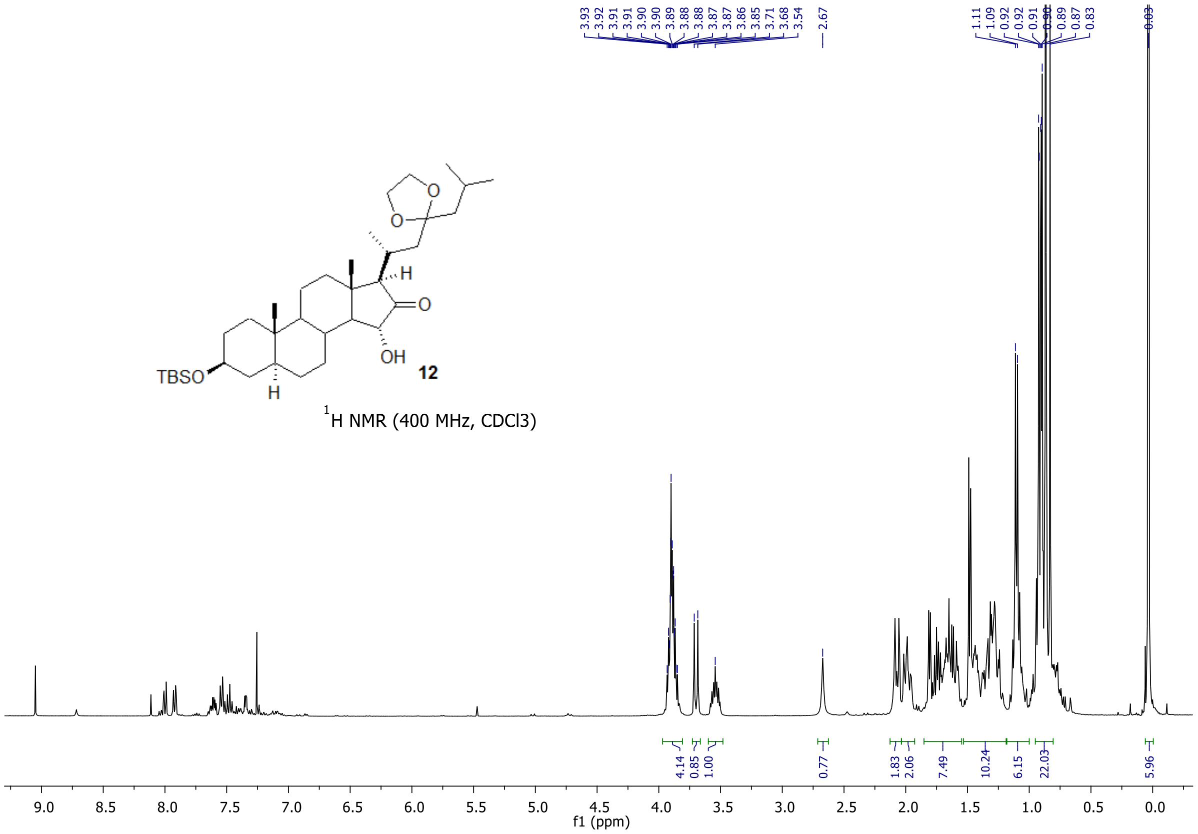

12

^{1}H NMR (400 MHz, CDCl3)

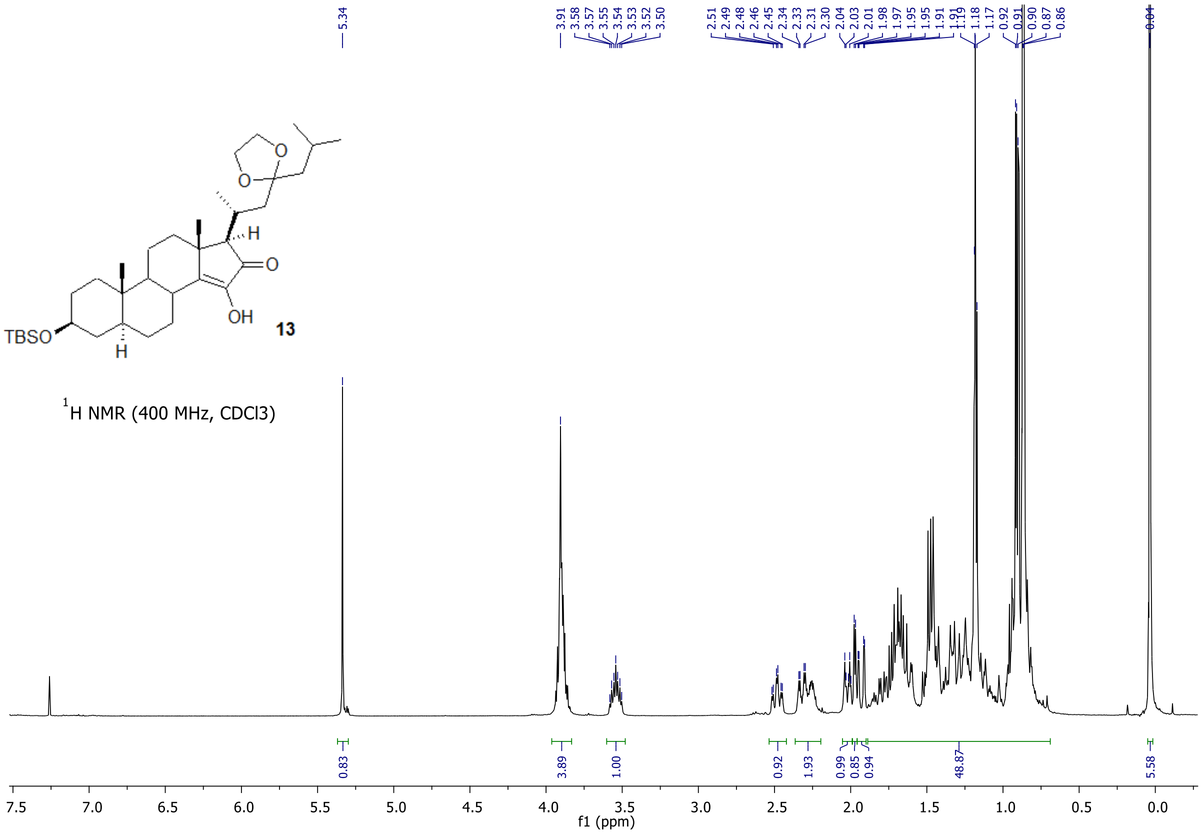
TBSO
OH
H
O
O
13
^{1}H NMR (400 MHz, CDCl3)
5.34
3.91 3.58 3.57 3.55 3.54 3.53 3.52 3.50
2.51 2.49 2.48 2.46 2.45 2.34 2.33 2.31 2.30 2.04 2.03 2.01 1.98 1.97 1.95 1.95 1.91 1.91 1.19 1.18 1.17 0.92 0.91 0.90 0.87 0.86
0.04
0.83 3.89 1.00 0.92 1.93 0.99 0.85 0.94 48.87 5.58
7.5 7.0 6.5 6.0 5.5 5.0 4.5 4.0 3.5 3.0 2.5 2.0 1.5 1.0 0.5 0.0
f1 (ppm)

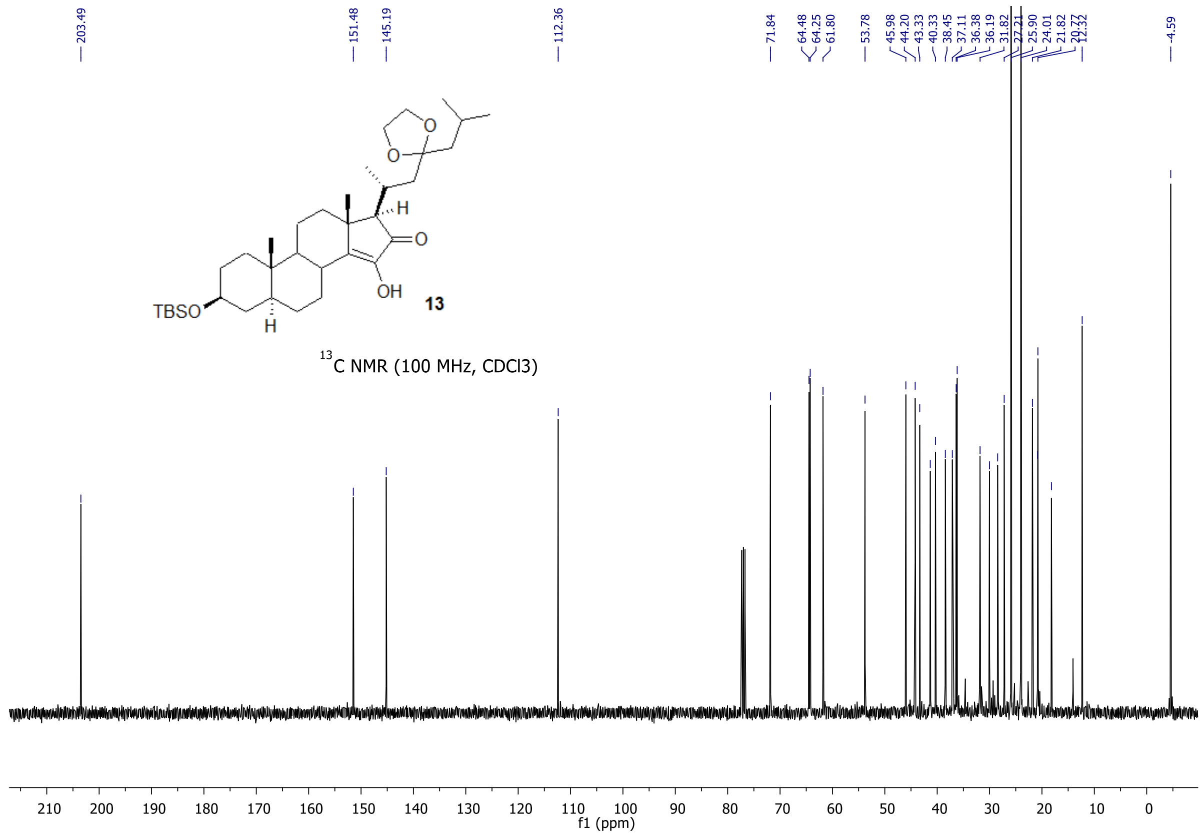
TBSO
OH
H
O
O
13
^{13}C NMR (100 MHz, CDCl3)
203.49
151.48
145.19
112.36
71.84 64.48 64.25 61.80
53.78 45.98 44.20 43.33 40.33 38.45 37.11 36.38 36.19 31.82 27.21 25.90 24.01 21.82 20.77 12.32
-4.59
210 200 190 180 170 160 150 140 130 120 110 100 90 80 70 60 50 40 30 20 10 0
f1 (ppm)

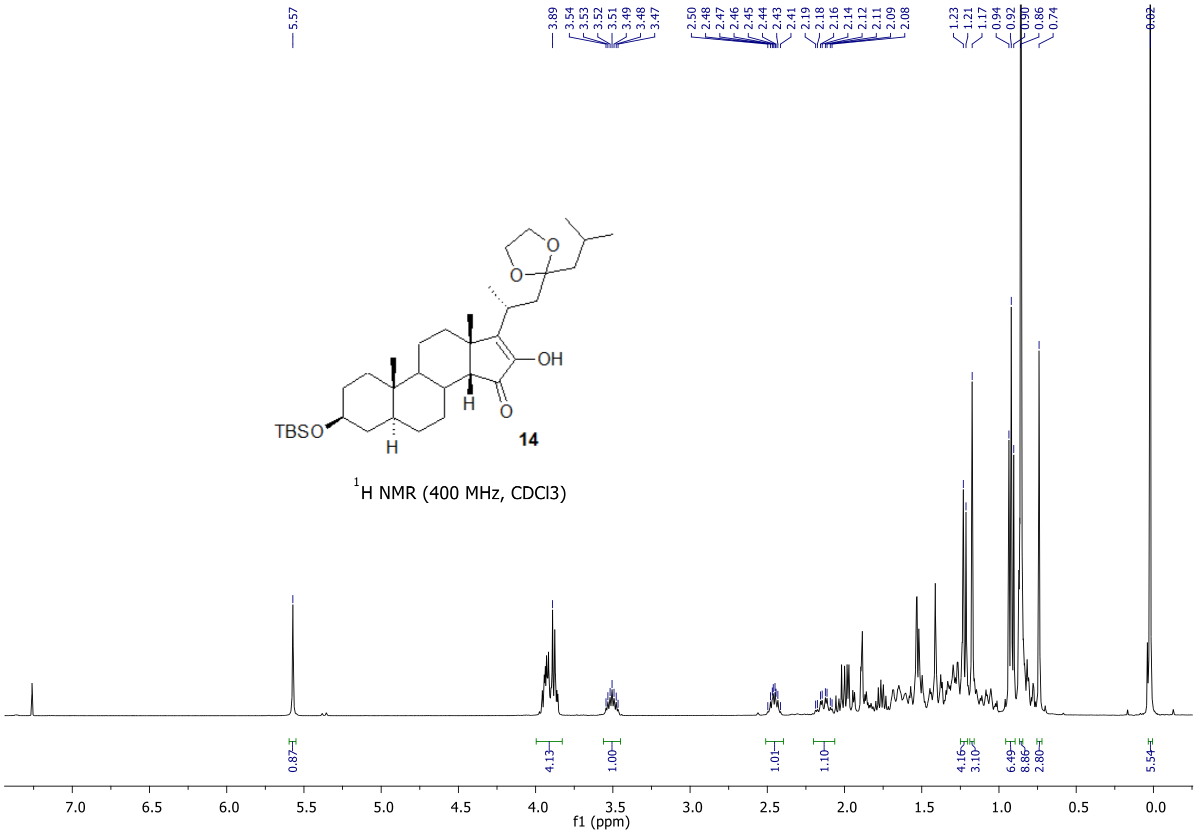
5.57
3.89
3.54
3.53
3.52
3.51
3.49
3.48
3.47
2.50
2.48
2.47
2.46
2.45
2.44
2.43
2.41
2.19
2.18
2.16
2.14
2.12
2.11
2.09
2.08
1.23
1.21
1.17
0.94
0.92
0.90
0.86
0.74
0.02
O
O
OH
TBSO
H
H
O
14
1H NMR (400 MHz, CDCl3)
0.87
4.13
1.00
1.01
1.10
4.16
3.10
6.49
8.86
2.80
5.54
7.0
6.5
6.0
5.5
5.0
4.5
4.0
3.5
3.0
2.5
2.0
1.5
1.0
0.5
0.0
f1 (ppm)

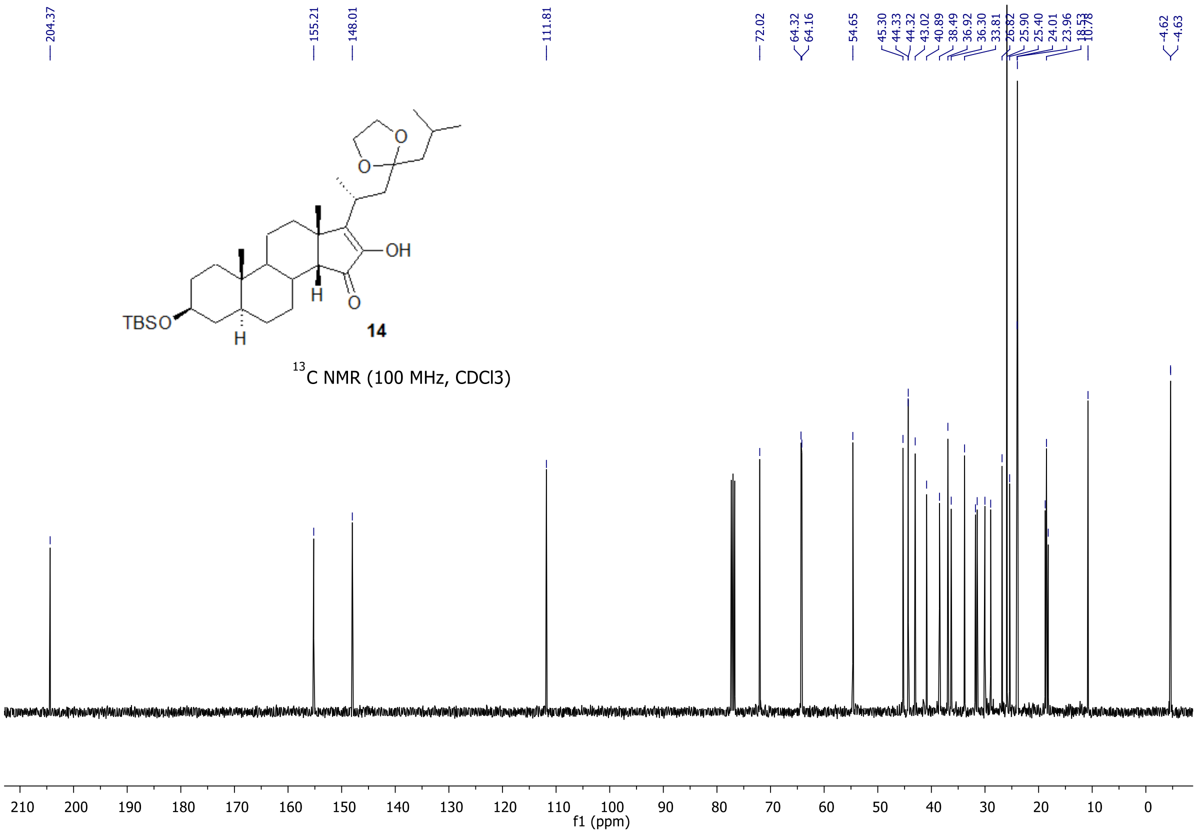
204.37
155.21
148.01
111.81
72.02
64.32
64.16
54.65
45.30
44.33
44.32
43.02
40.89
38.49
36.92
36.30
33.81
26.82
25.90
25.40
24.01
23.96
-4.62
-4.63
O
O
OH
TBSO
H
H
O
14
13C NMR (100 MHz, CDCl3)
210
200
190
180
170
160
150
140
130
120
110
100
90
80
70
60
50
40
30
20
10
0
f1 (ppm)

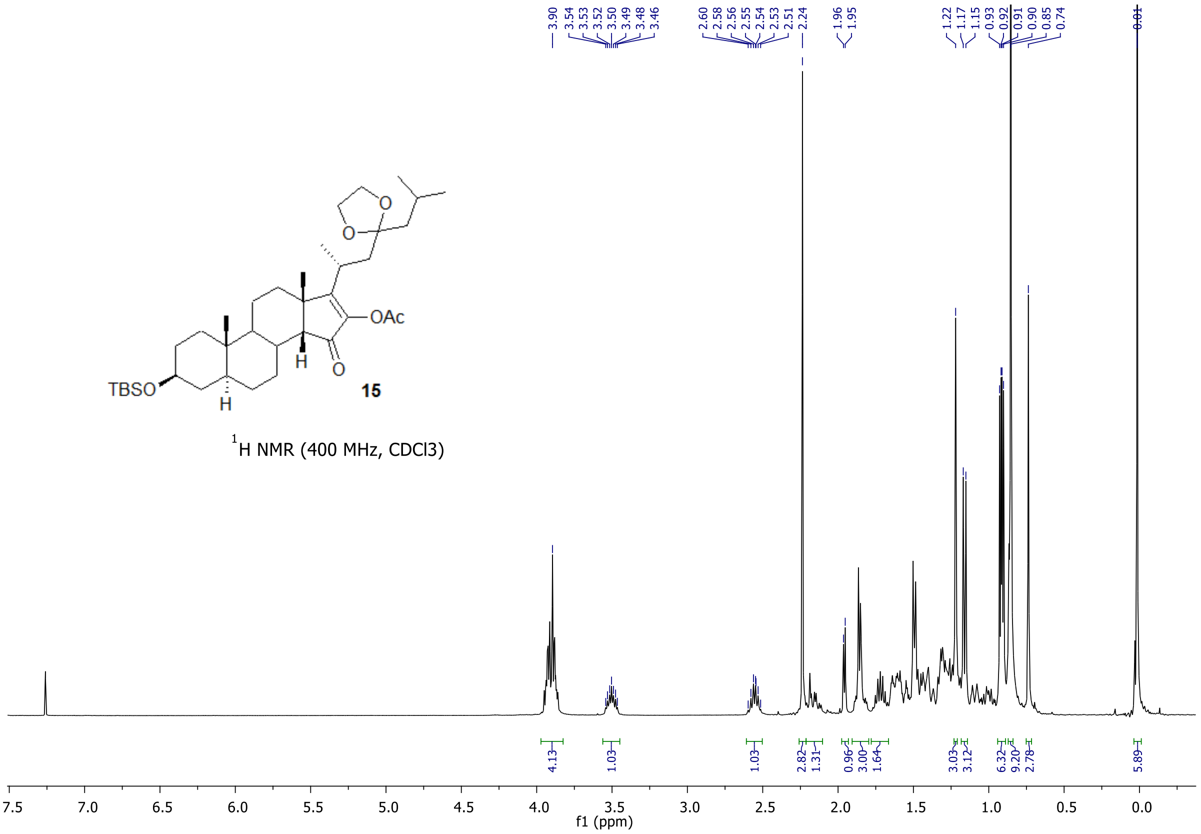
3.90 3.54 3.53 3.52 3.50 3.49 3.48 3.46 2.60 2.58 2.56 2.55 2.54 2.53 2.51 2.24 1.96 1.95 1.22 1.17 1.15 0.93 0.92 0.91 0.90 0.85 0.74 0.01
O
O
OAc
H
O
TBSO
H
15
1H NMR (400 MHz, CDCl3)
4.13 1.03 1.03 2.82 1.31 0.96 3.00 1.64 3.03 3.12 6.32 9.20 2.78 5.89
7.5 7.0 6.5 6.0 5.5 5.0 4.5 4.0 3.5 3.0 2.5 2.0 1.5 1.0 0.5 0.0
f1 (ppm)

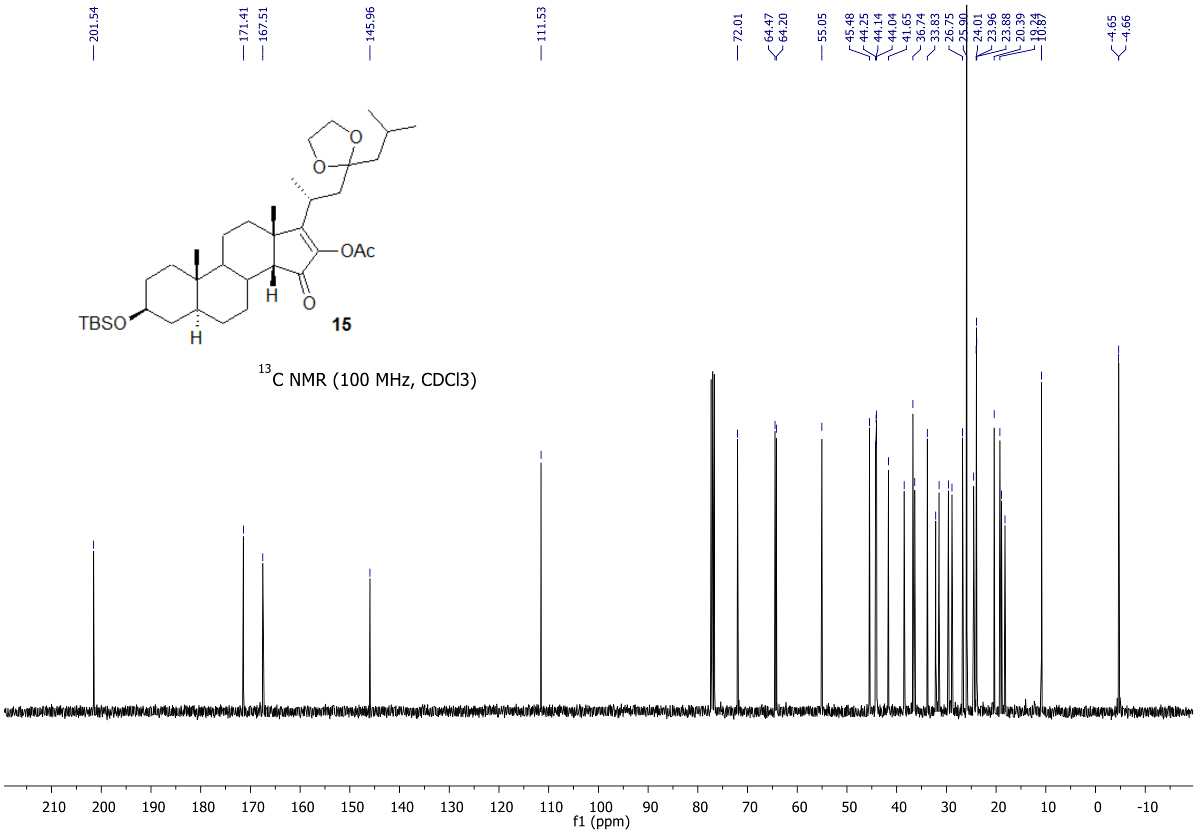
201.54 171.41 167.51 145.96 111.53 72.01 64.47 64.20 55.05 45.48 44.25 44.14 44.04 41.65 36.74 33.83 26.75 25.90 24.01 23.96 23.88 20.39 19.24 10.87 -4.65 -4.66
O
O
OAc
H
O
TBSO
H
15
13C NMR (100 MHz, CDCl3)
210 200 190 180 170 160 150 140 130 120 110 100 90 80 70 60 50 40 30 20 10 0 -10
f1 (ppm)

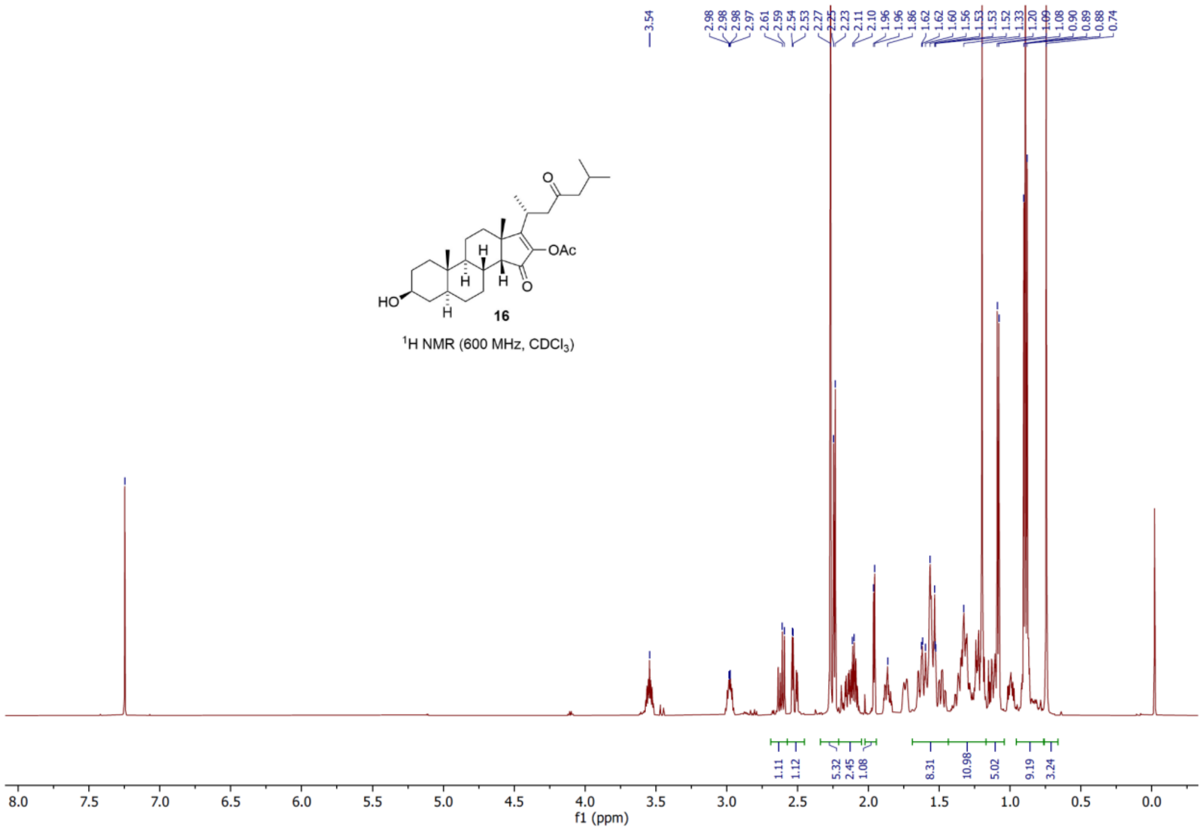
3.54
2.98 2.98 2.98 2.97 2.61 2.59 2.54 2.53 2.27 2.25 2.23 2.11 2.10 1.96 1.96 1.86 1.62 1.62 1.60 1.56 1.53 1.53 1.52 1.33 1.20 1.09 1.08 0.90 0.89 0.88 0.74
OAc
O
HO
H
16
1H NMR (600 MHz, CDCl3)
1.11 1.12 5.32 2.45 1.08 8.31 10.98 5.02 9.19 3.24
8.0 7.5 7.0 6.5 6.0 5.5 5.0 4.5 4.0 3.5 3.0 2.5 2.0 1.5 1.0 0.5 0.0
f1 (ppm)

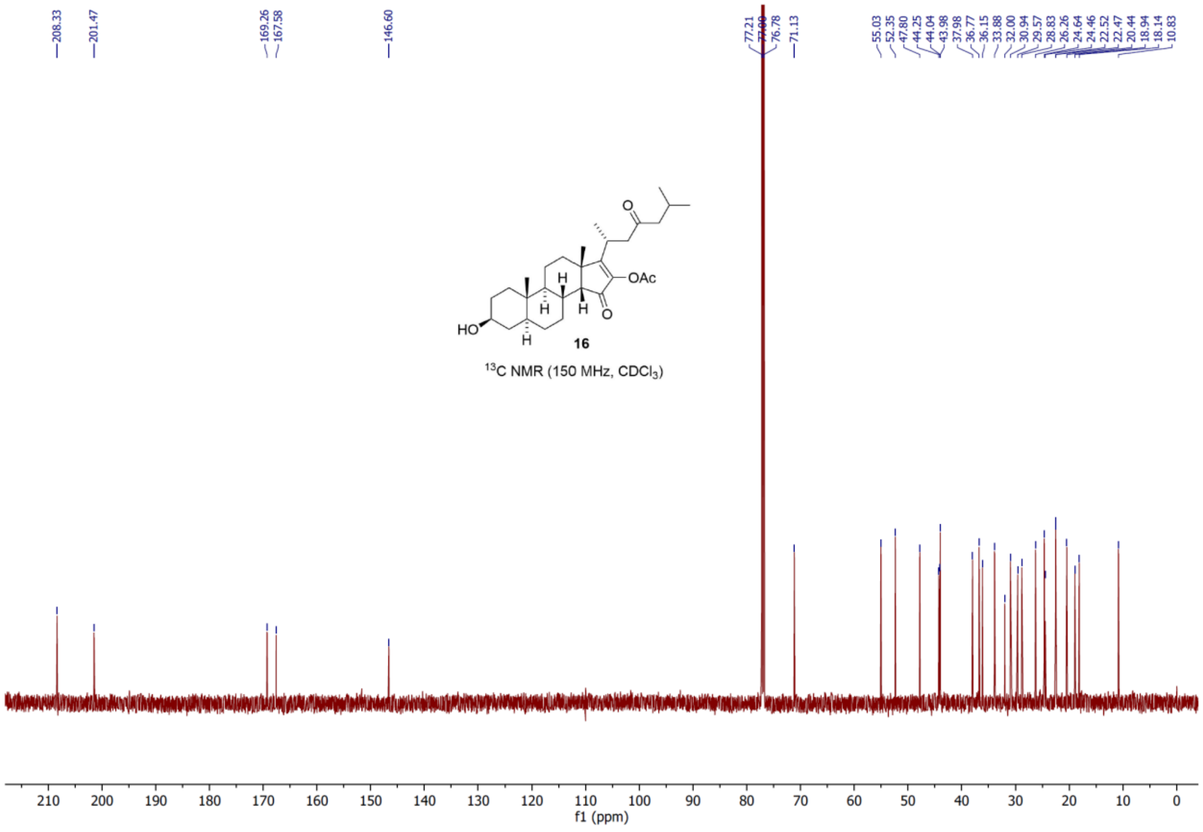
208.33
201.47
169.26 167.58
146.60
77.21 77.00 76.78
71.13
55.03 52.35 47.80 44.25 44.04 43.98 37.98 36.77 36.15 33.88 32.00 30.94 29.57 28.83 26.26 24.64 24.46 22.52 22.47 20.44 18.94 18.14 10.83
OAc
O
HO
H
16
13C NMR (150 MHz, CDCl3)
210 200 190 180 170 160 150 140 130 120 110 100 90 80 70 60 50 40 30 20 10 0
f1 (ppm)

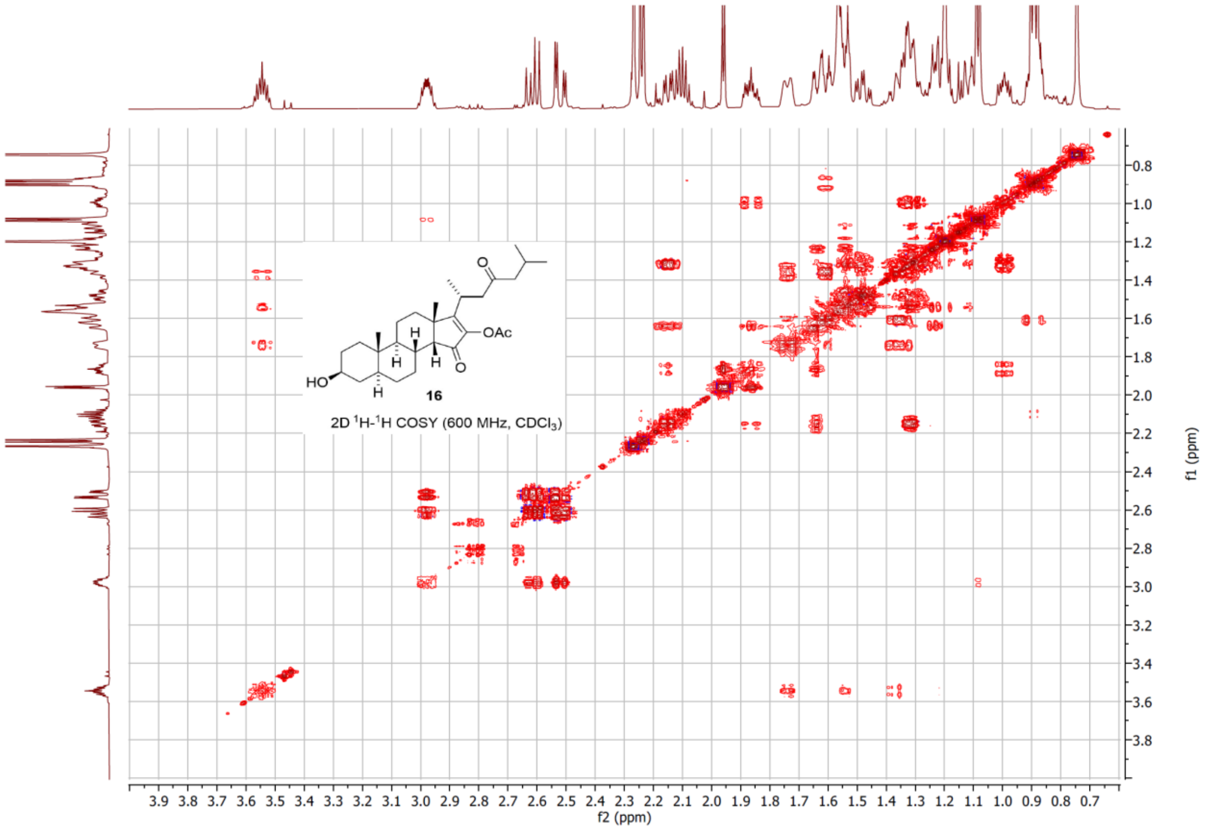
OAc
HO
H
16
2D ¹H-¹H COSY (600 MHz, CDCl₃)
f1 (ppm)
f2 (ppm)

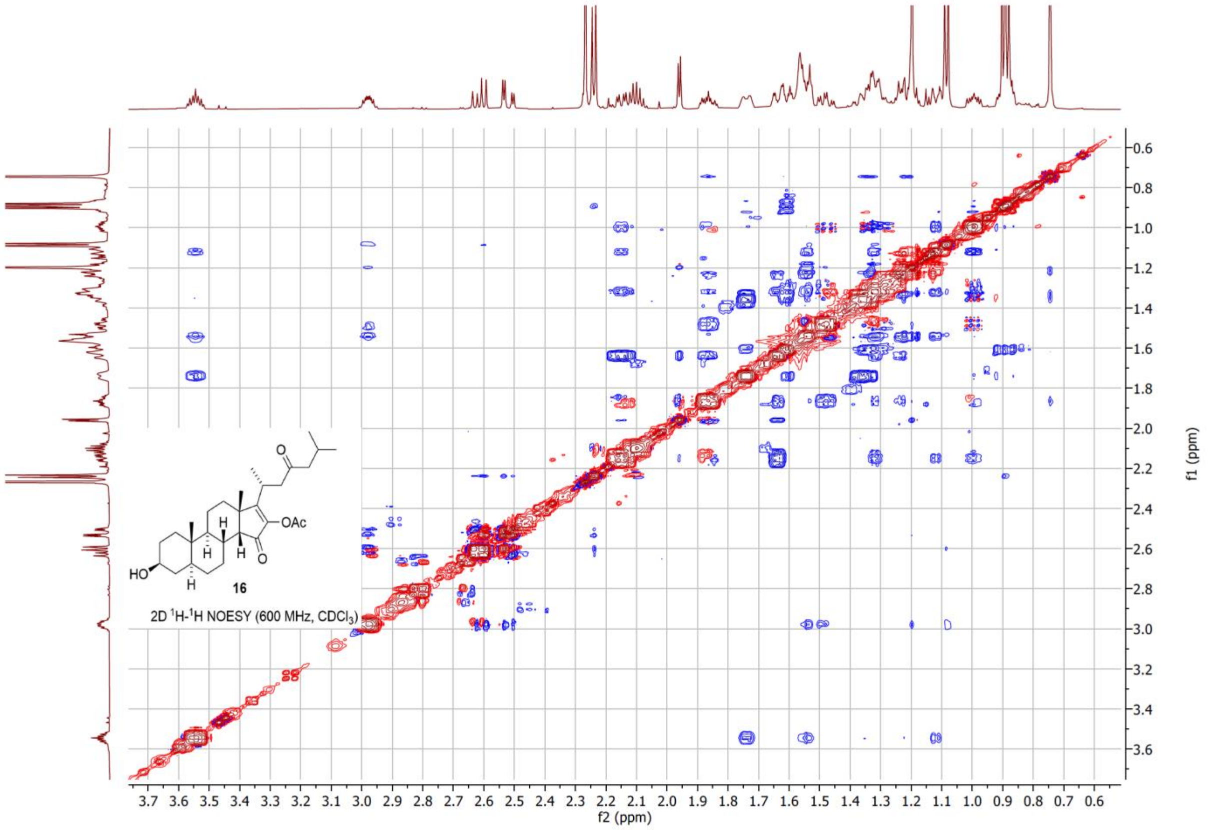
OAc
HO
H
16
2D ¹H-¹H NOESY (600 MHz, CDCl₃)
f1 (ppm)
f2 (ppm)

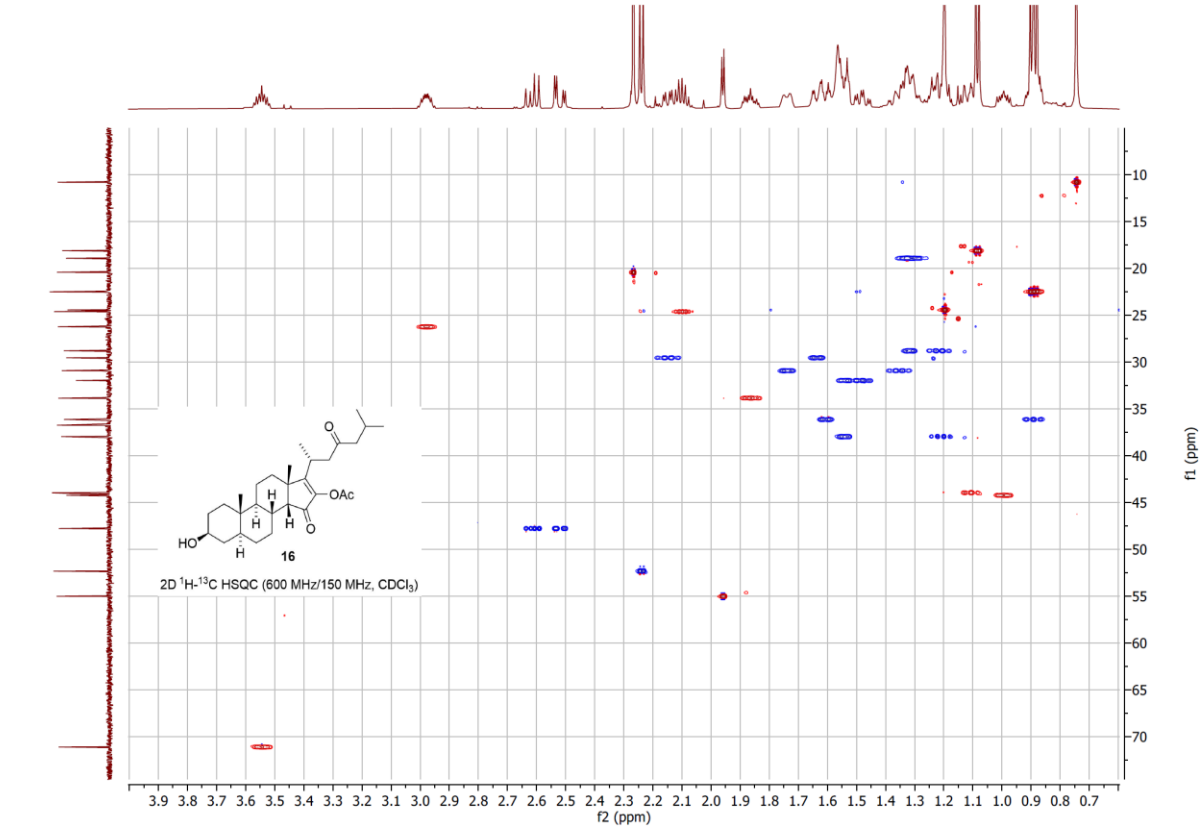

2D ^{1}H-^{13}C HSQC (600 MHz/150 MHz, $CDCl_3$)

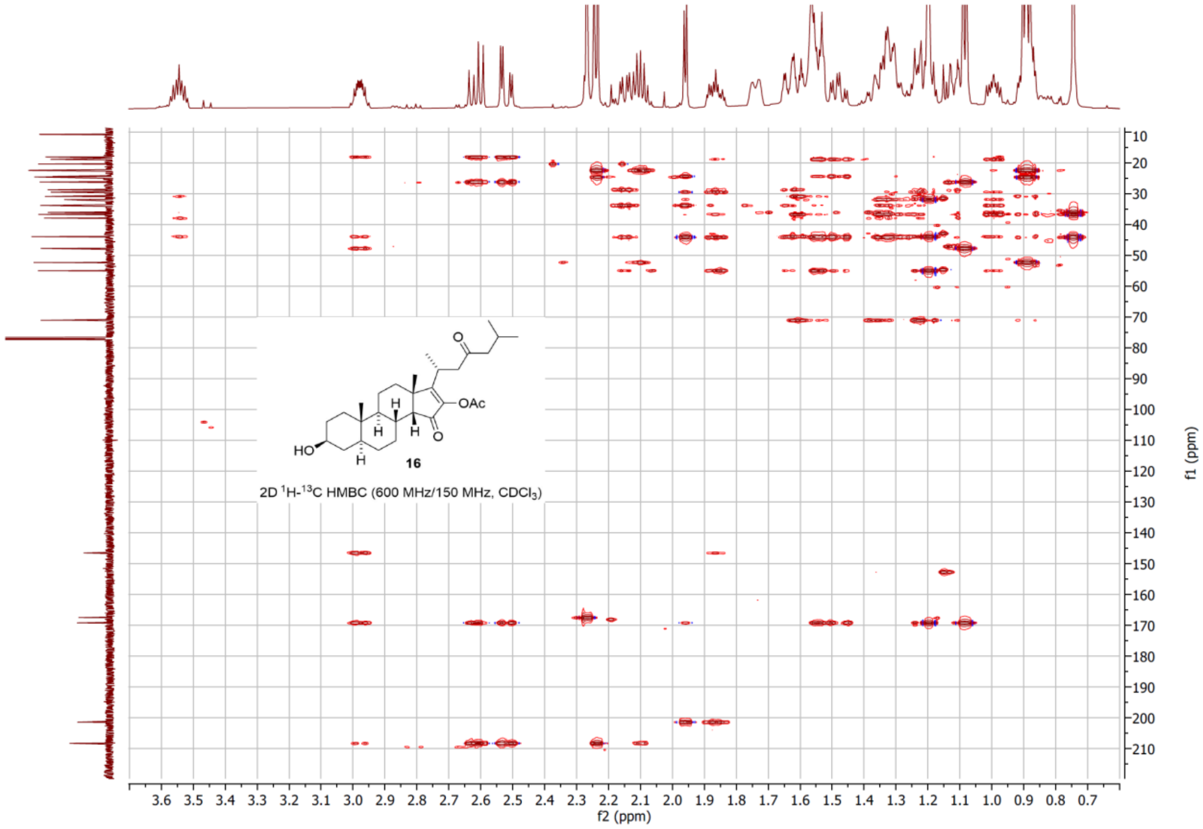

2D ^{1}H-^{13}C HMBC (600 MHz/150 MHz, $CDCl_3$)

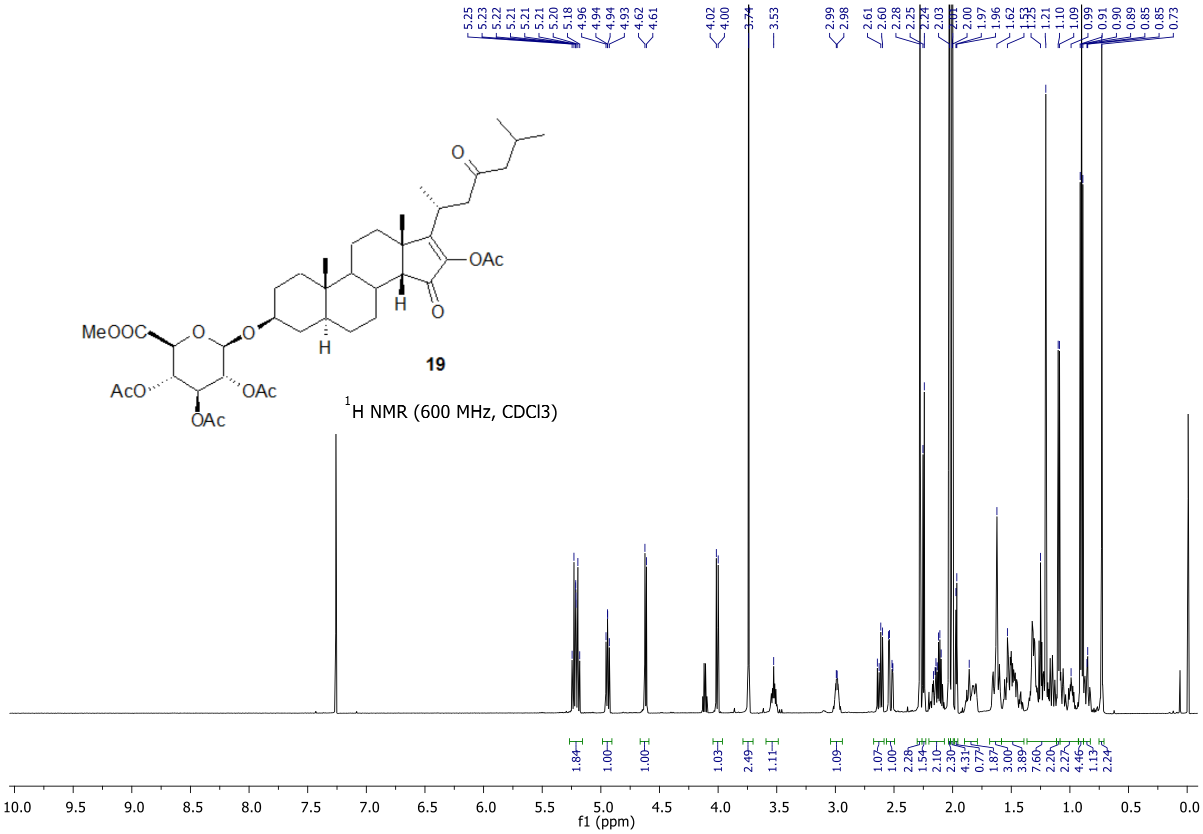
MeOOC
AcO
OAc
OAc
OAc
O
H
19
1H NMR (600 MHz, CDCl3)
f1 (ppm)

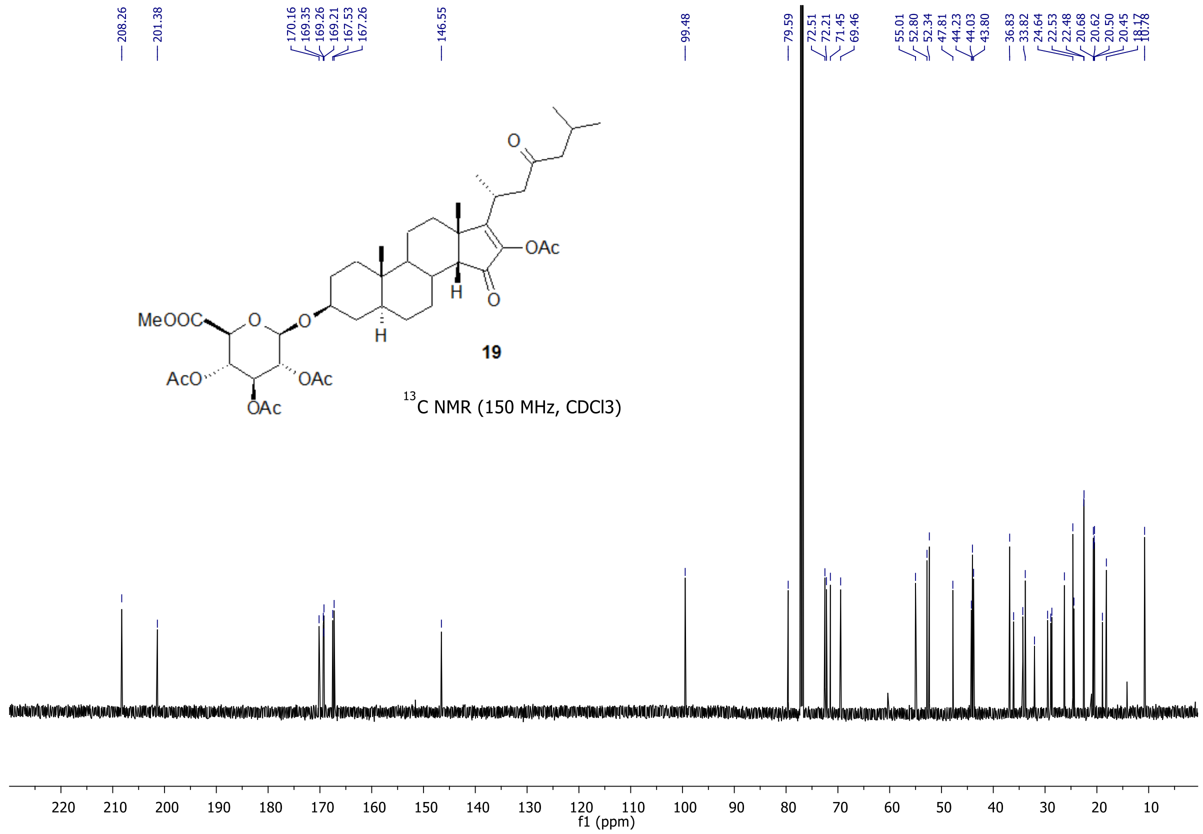
MeOOC
AcO
OAc
OAc
OAc
O
H
19
13C NMR (150 MHz, CDCl3)
f1 (ppm)

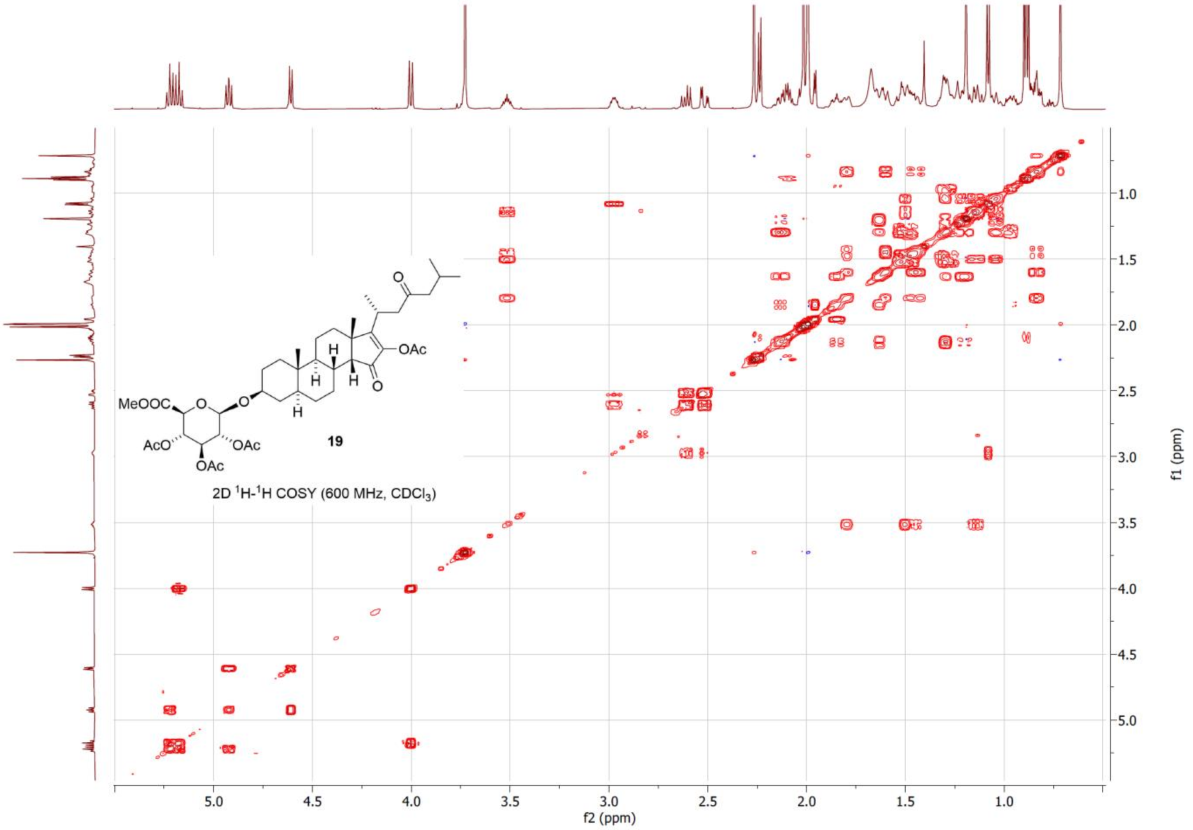
MeOOC
AcO
OAc
OAc
OAc
19
2D 1H-1H COSY (600 MHz, CDCl3)
f2 (ppm)
f1 (ppm)

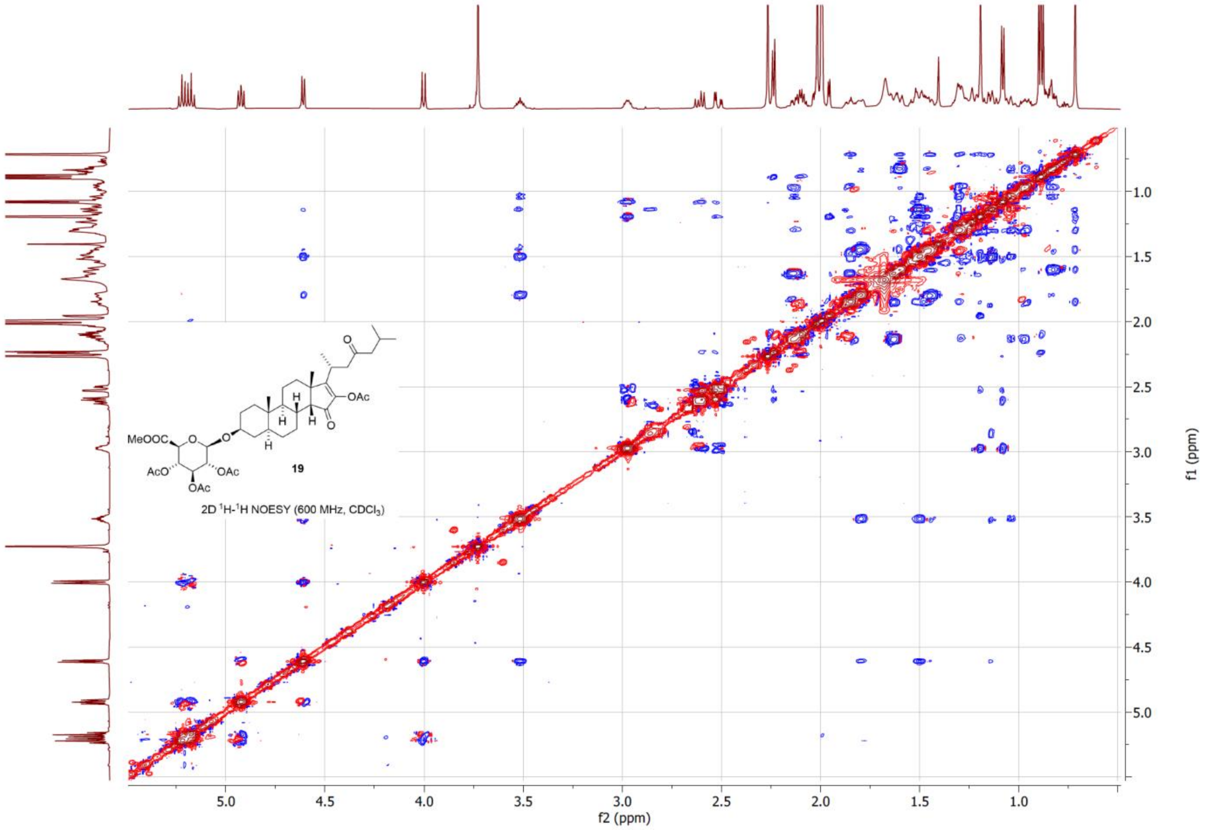
MeOOC
AcO
OAc
OAc
OAc
19
2D 1H-1H NOESY (600 MHz, CDCl3)
f2 (ppm)
f1 (ppm)

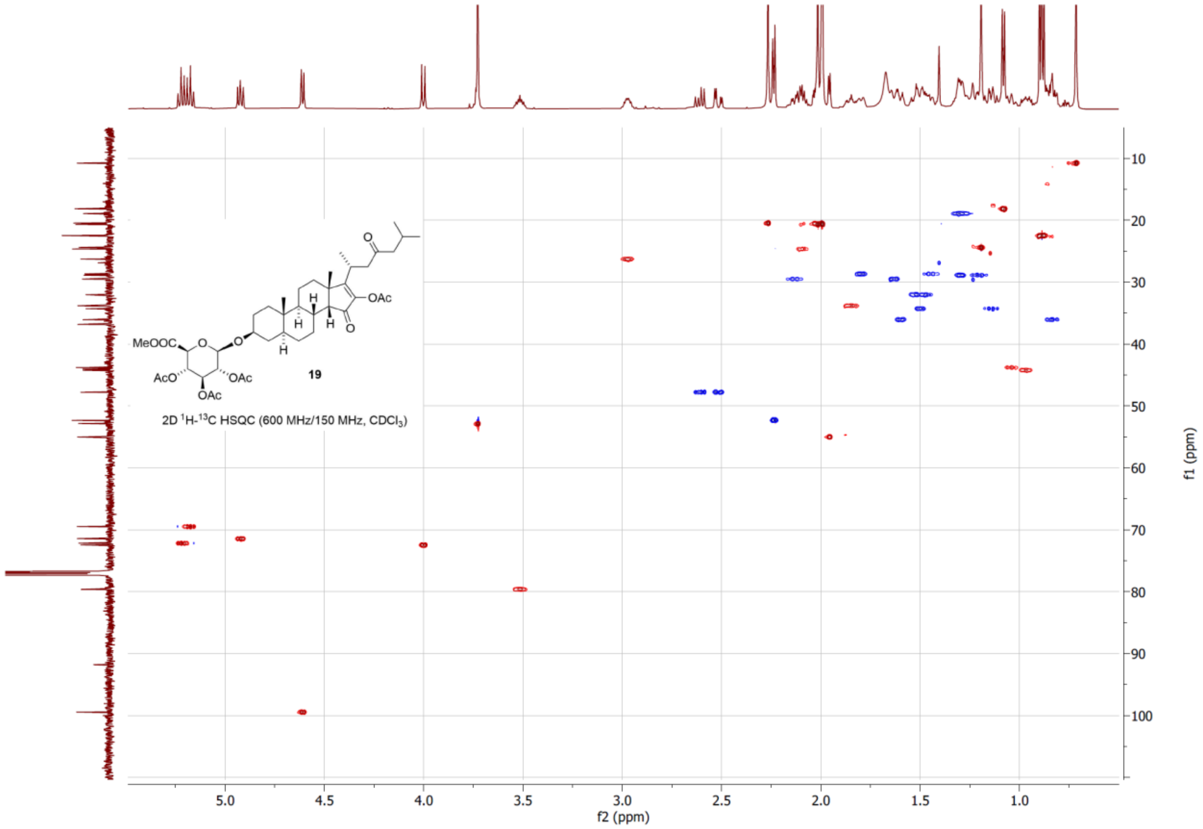
MeOOC
AcO
OAc
OAc
O
OAc
19
2D ¹H-¹³C HSQC (600 MHz/150 MHz, CDCl₃)
f1 (ppm)
f2 (ppm)

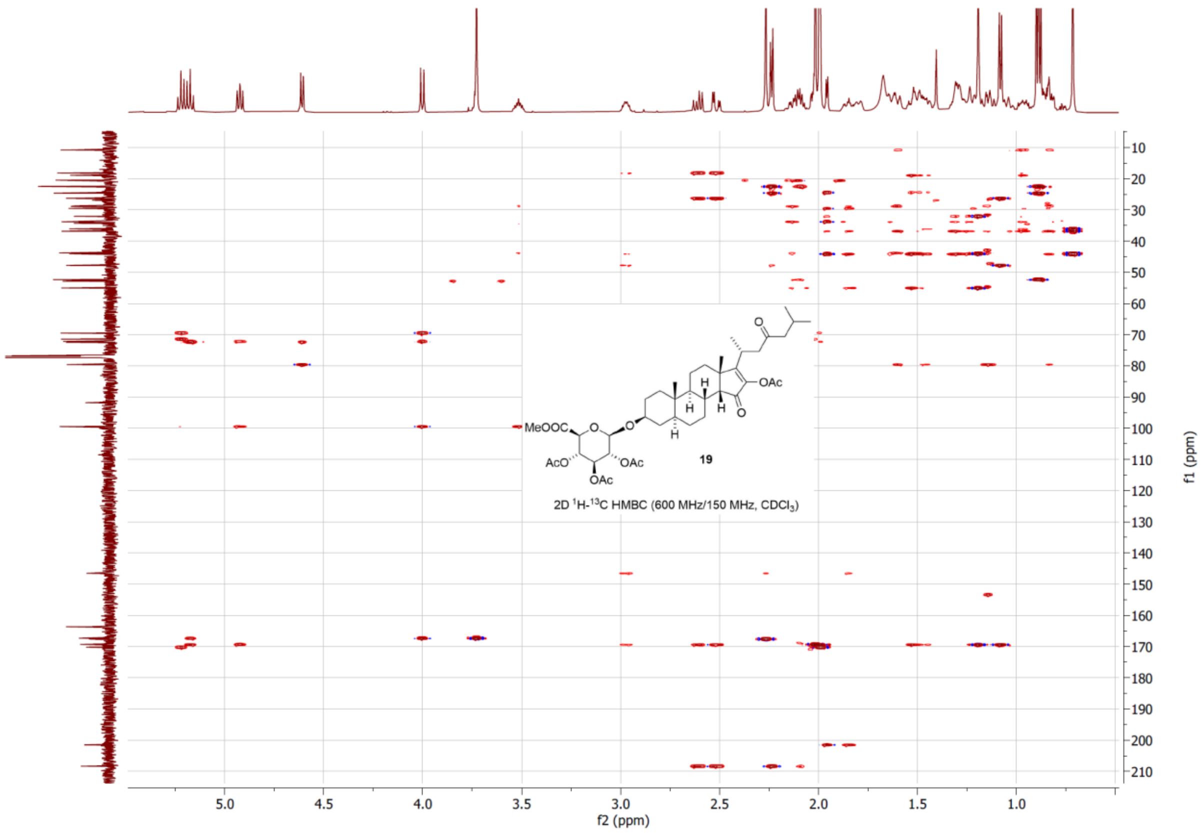
MeOOC
AcO
OAc
OAc
O
OAc
19
2D ¹H-¹³C HMBC (600 MHz/150 MHz, CDCl₃)
f1 (ppm)
f2 (ppm)

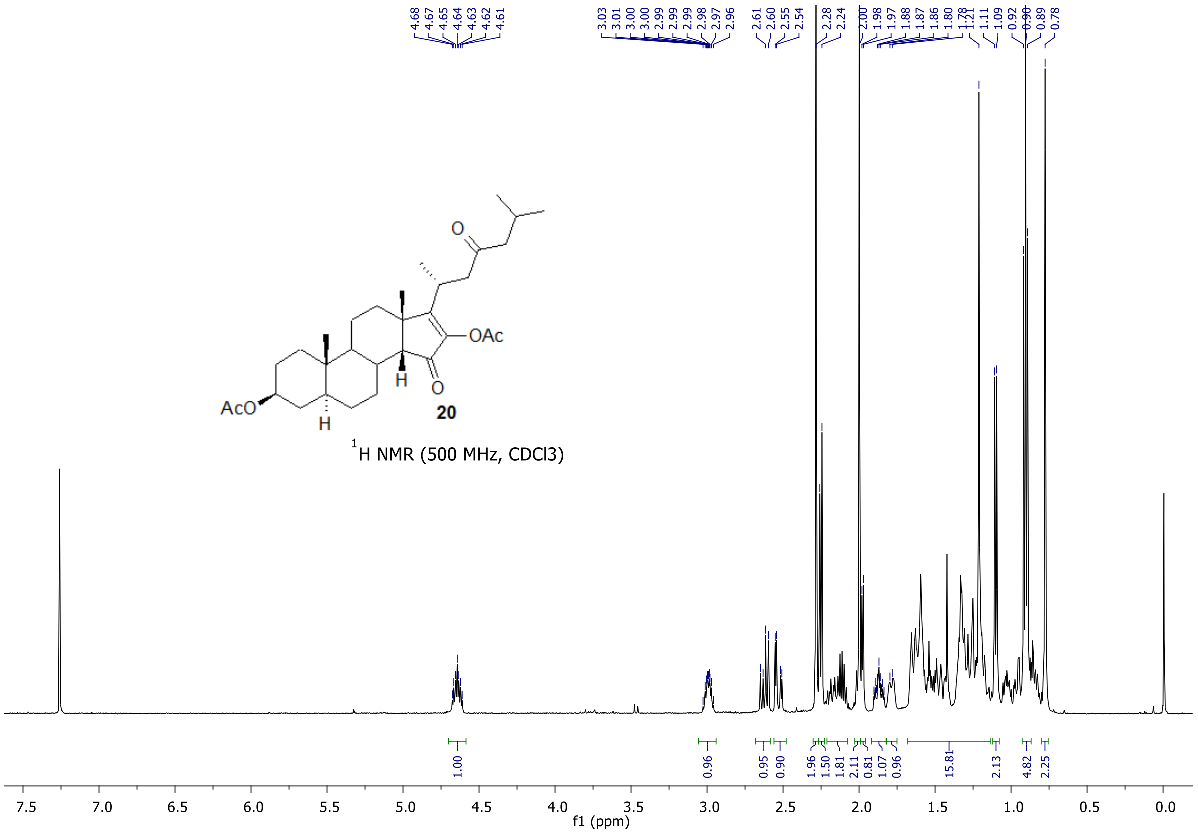

O
OAc
AcO
H
H
O
20
^{1}H NMR (500 MHz, CDCl3)
f1 (ppm)

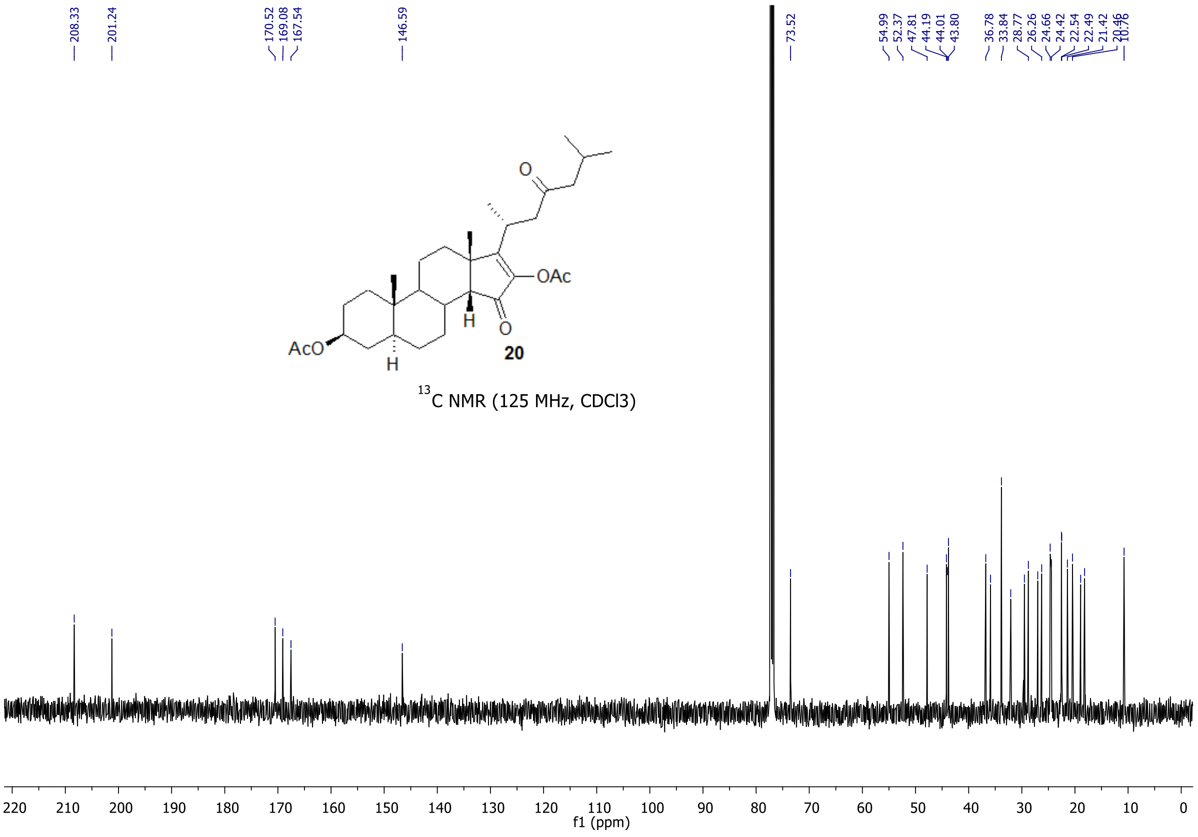

O
OAc
AcO
H
H
O
20
^{13}C NMR (125 MHz, CDCl3)
f1 (ppm)

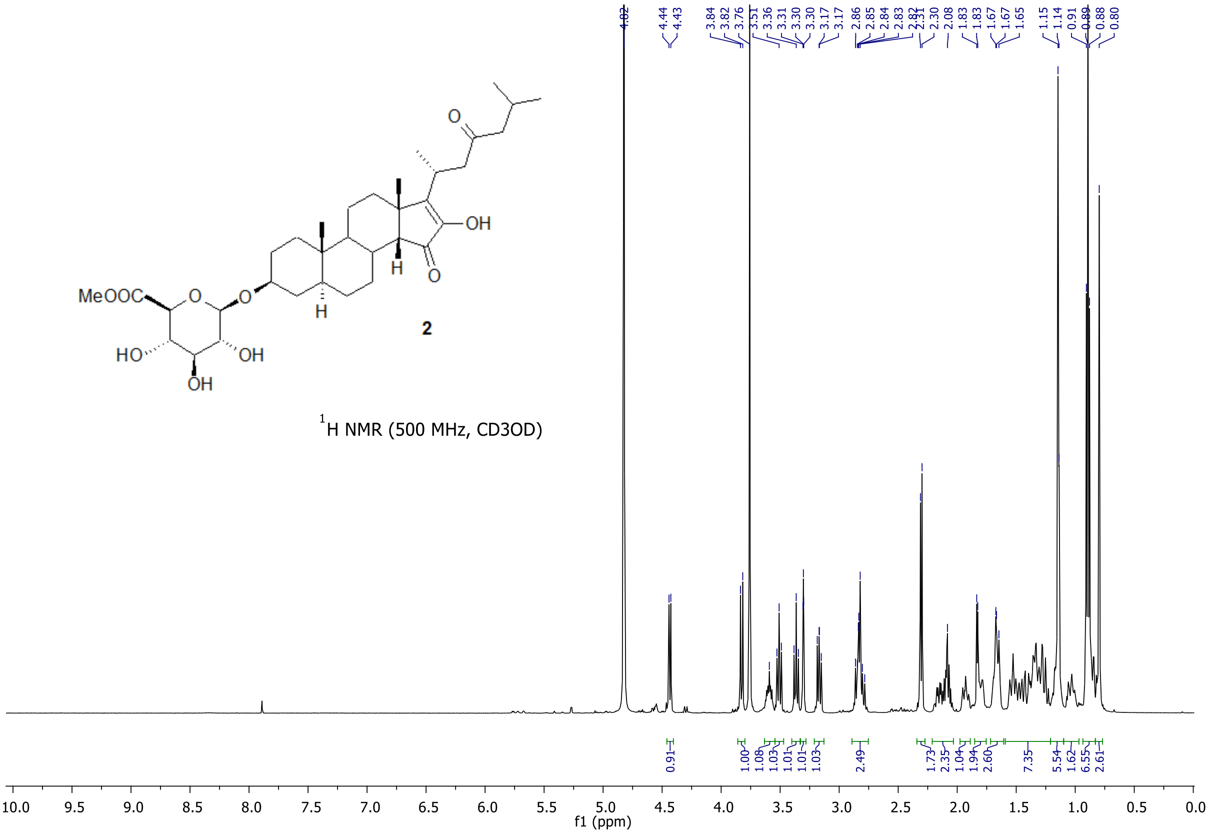
MeOOC
O
HO
OH
OH
H
H
OH
2
^{1}H NMR (500 MHz, CD3OD)
f1 (ppm)

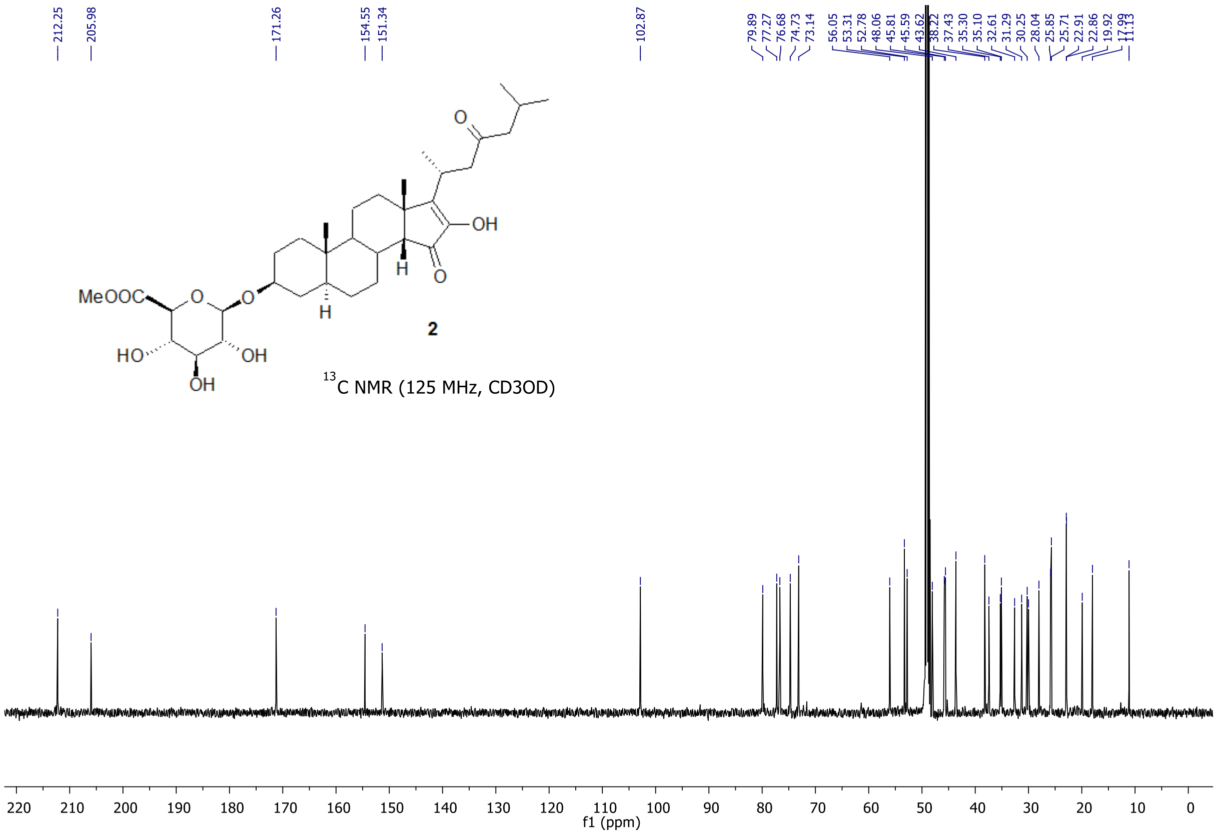
MeOOC
O
HO
OH
OH
H
H
OH
2
^{13}C NMR (125 MHz, CD3OD)
f1 (ppm)

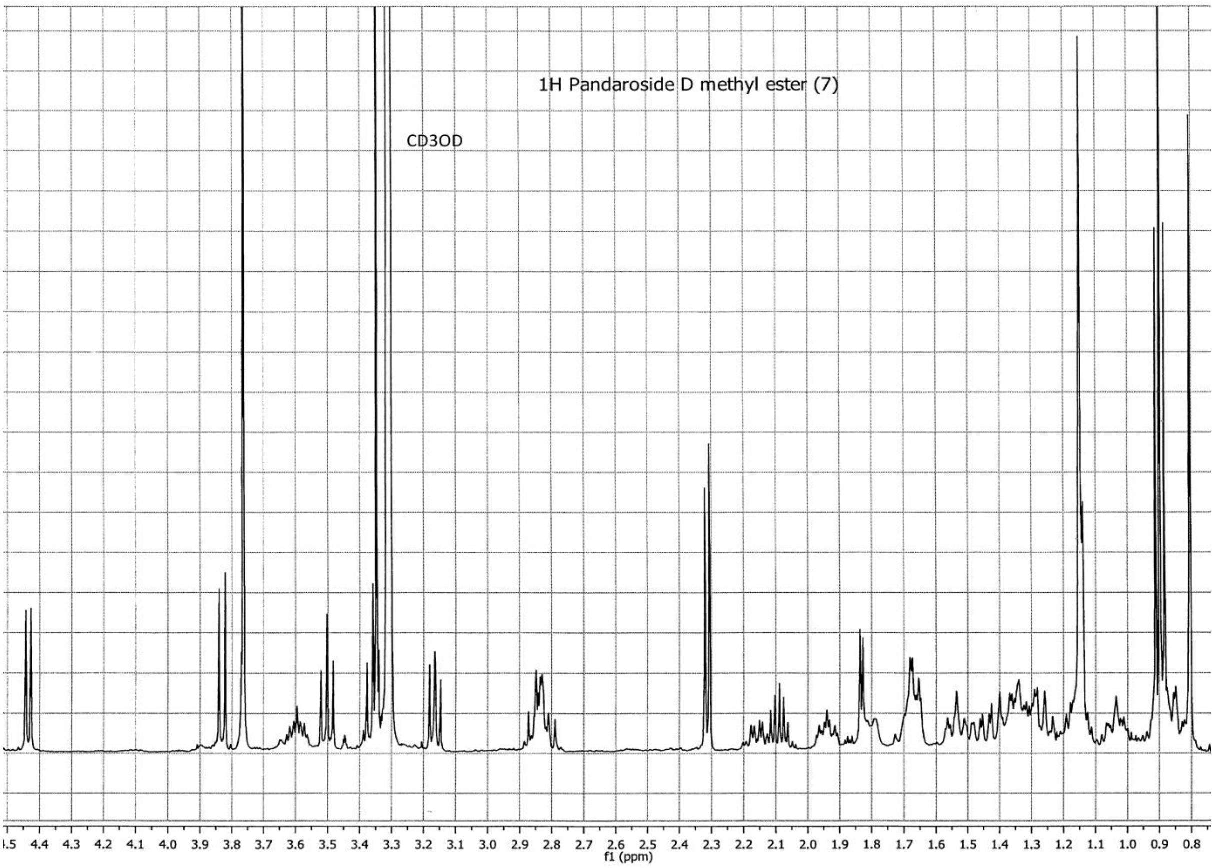

^{1}H NMR spectrum of methyl ester of Pandaroside Disolated from *Pandaros acanthifolium* (*Steroids* **2009**, *74*, 746–750).

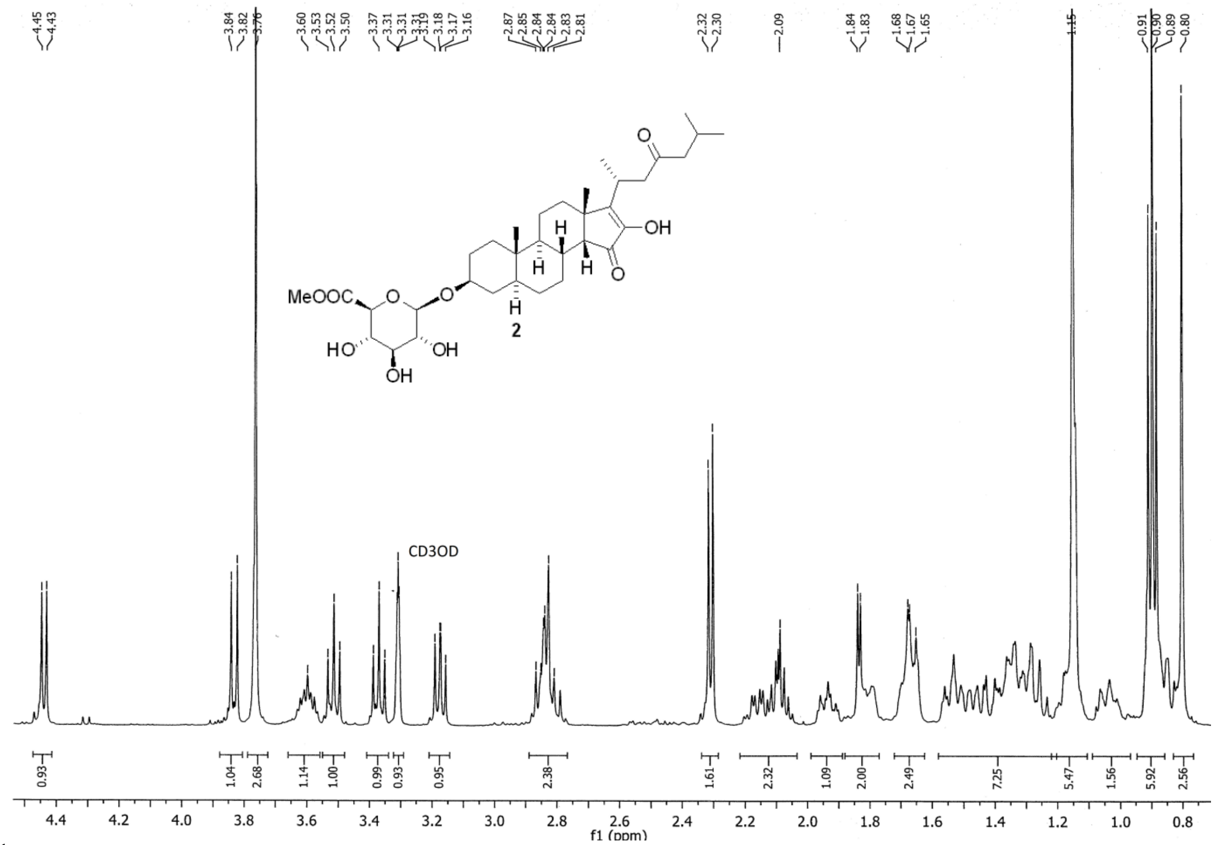

^{1}H NMR spectrum of the synthesised methyl ester of Pandaroside D; this work

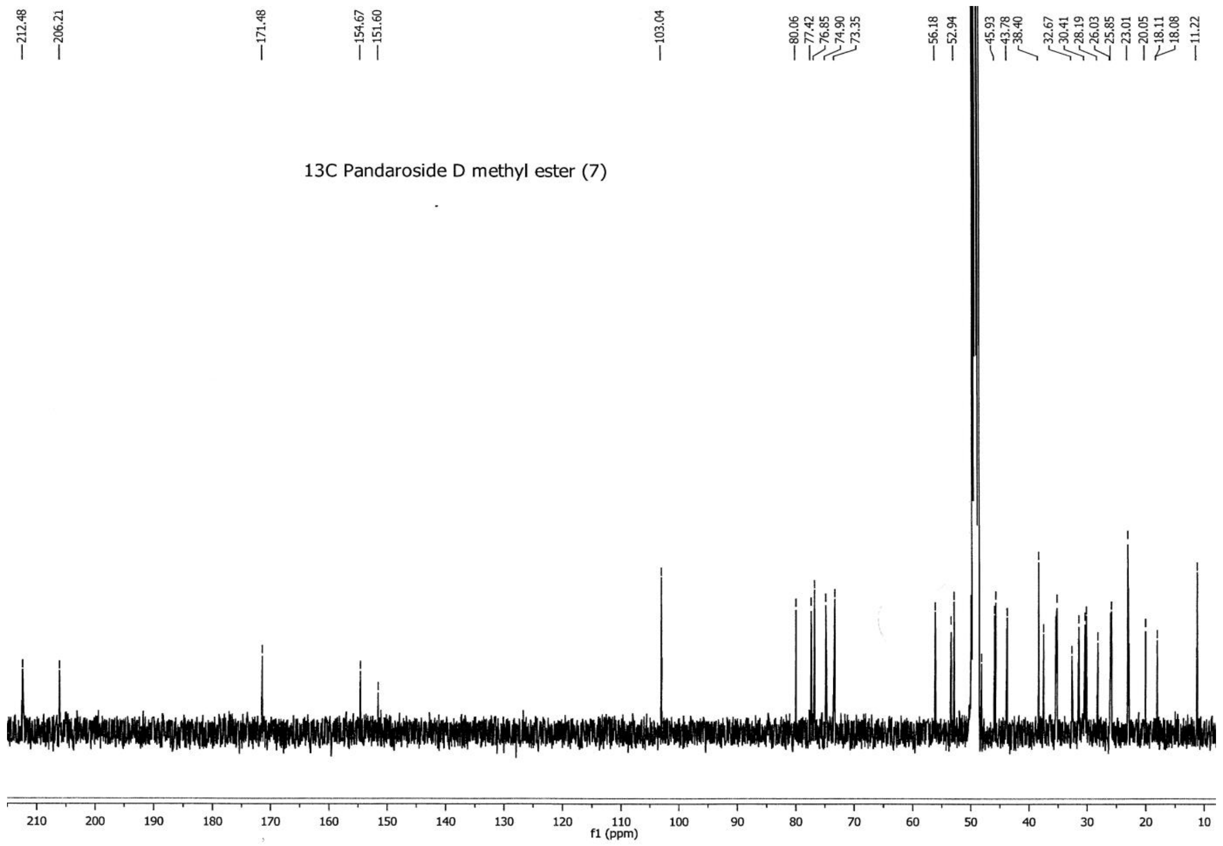

^{13}C NMR spectrum of methyl ester of Pandaroside Disolated from *Pandaros acanthifolium* (*Steroids* **2009**, *74*, 746–750).

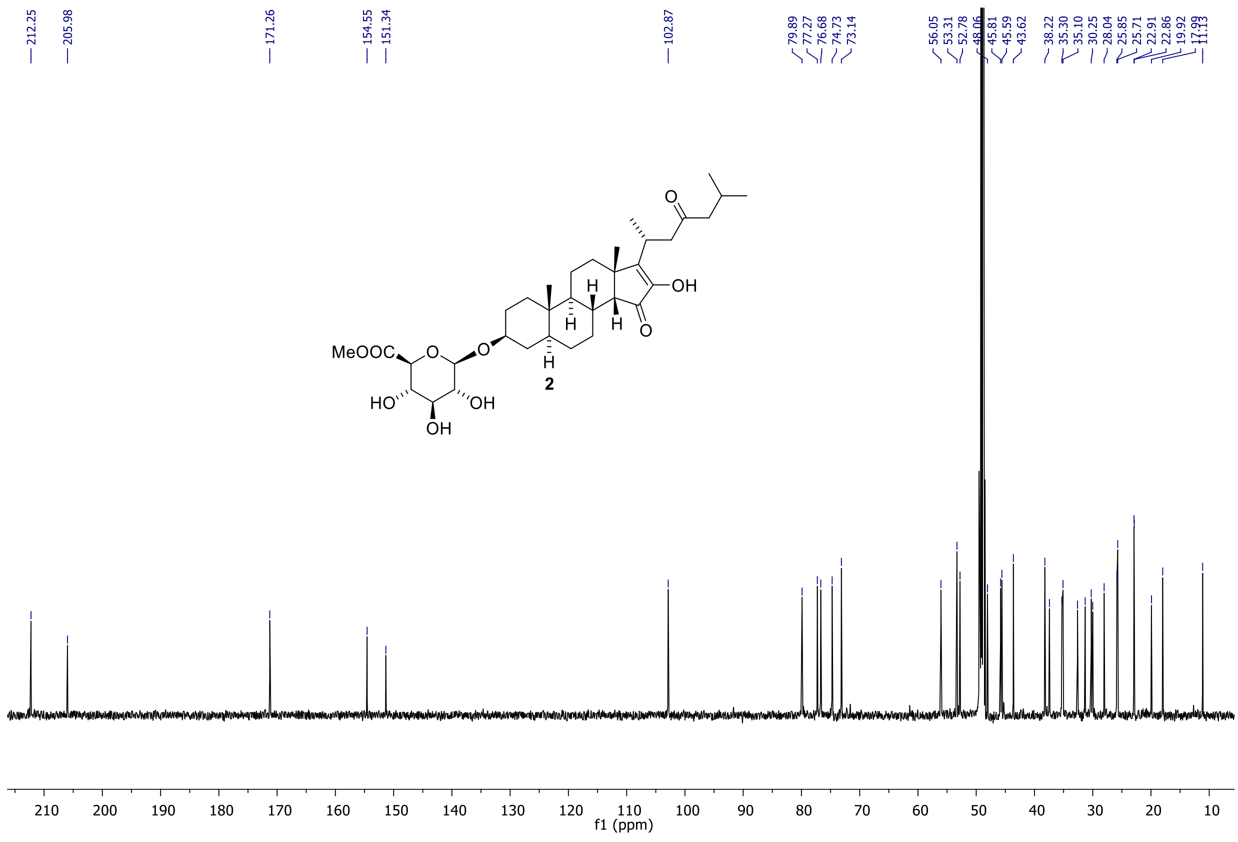

^{13}C NMR spectrum of the synthesised methyl ester of Pandaroside D; this work

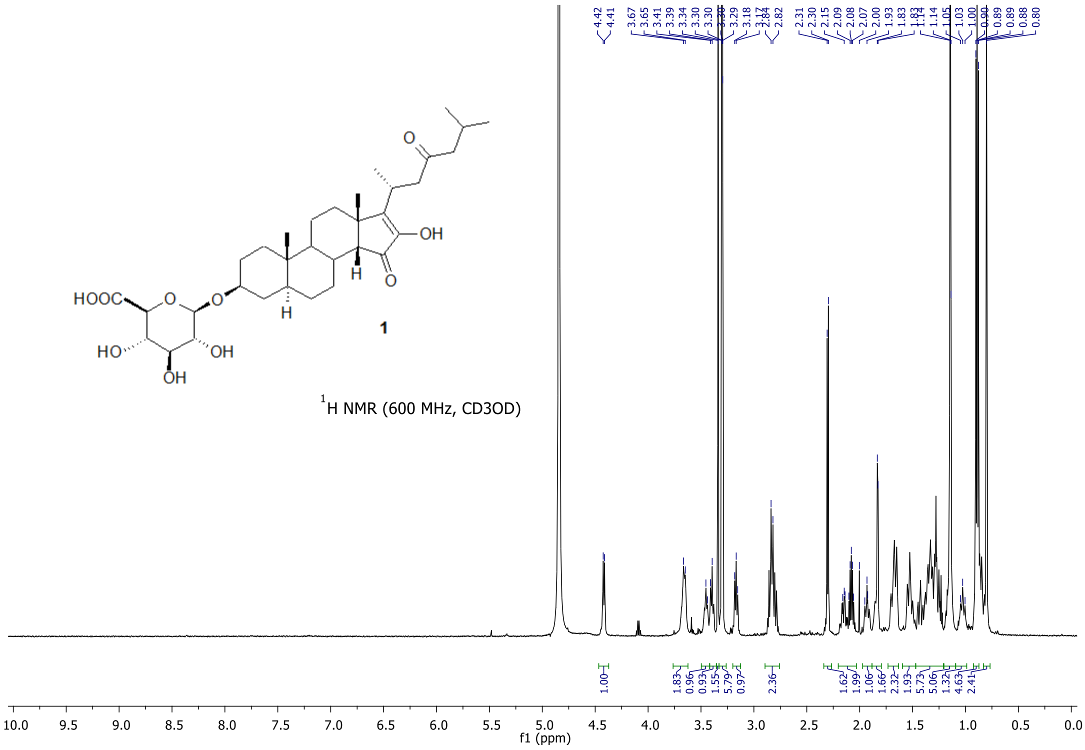
HOOC
OH
HO
H
1
1H NMR (600 MHz, CD3OD)
f1 (ppm)

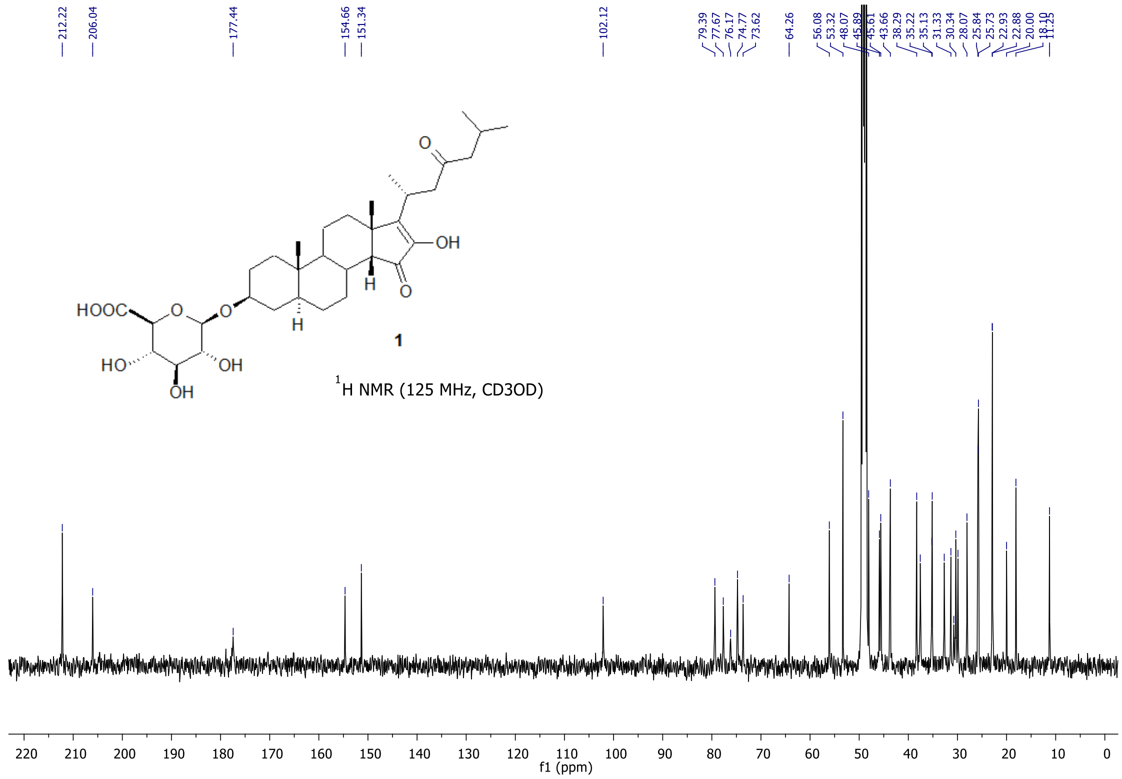
HOOC
OH
HO
H
1
1H NMR (125 MHz, CD3OD)
f1 (ppm)

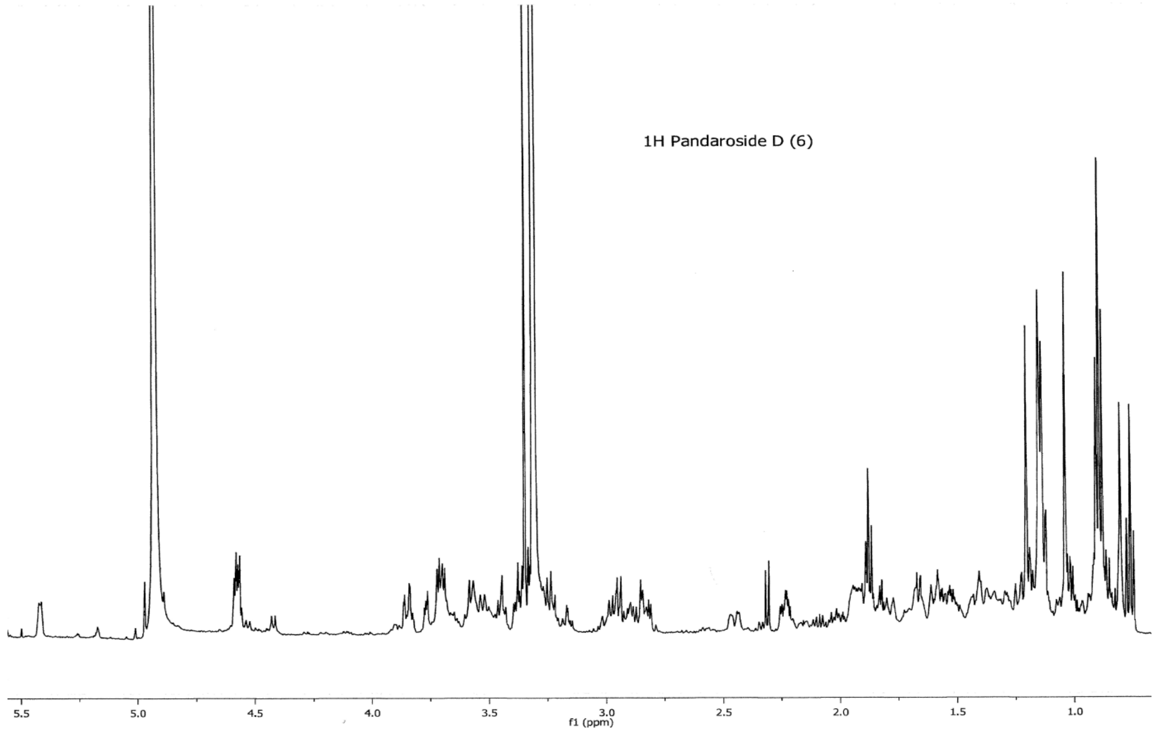

^{1}H NMR spectrum of Pandaroside Disolated from *Pandaros acanthifolium* (*Steroids* **2009**, *74*, 746–750).

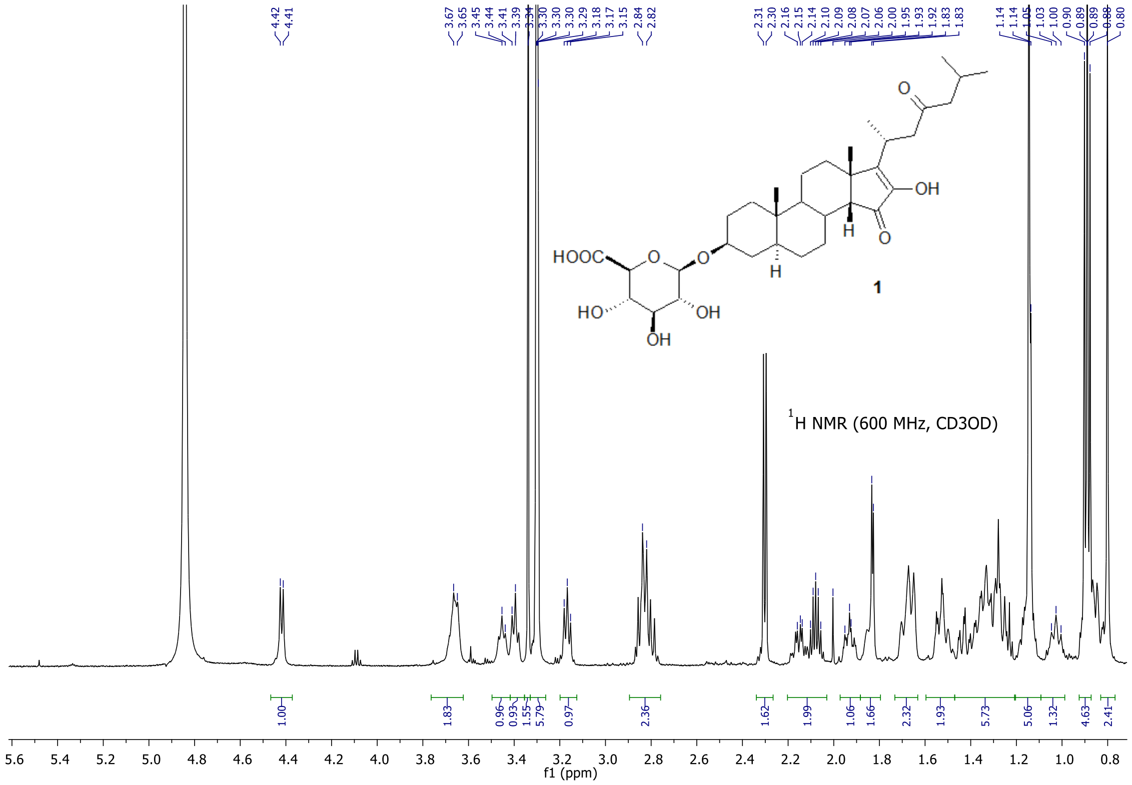

^{1}H NMR spectrum of the synthesised Pandaroside D; this work

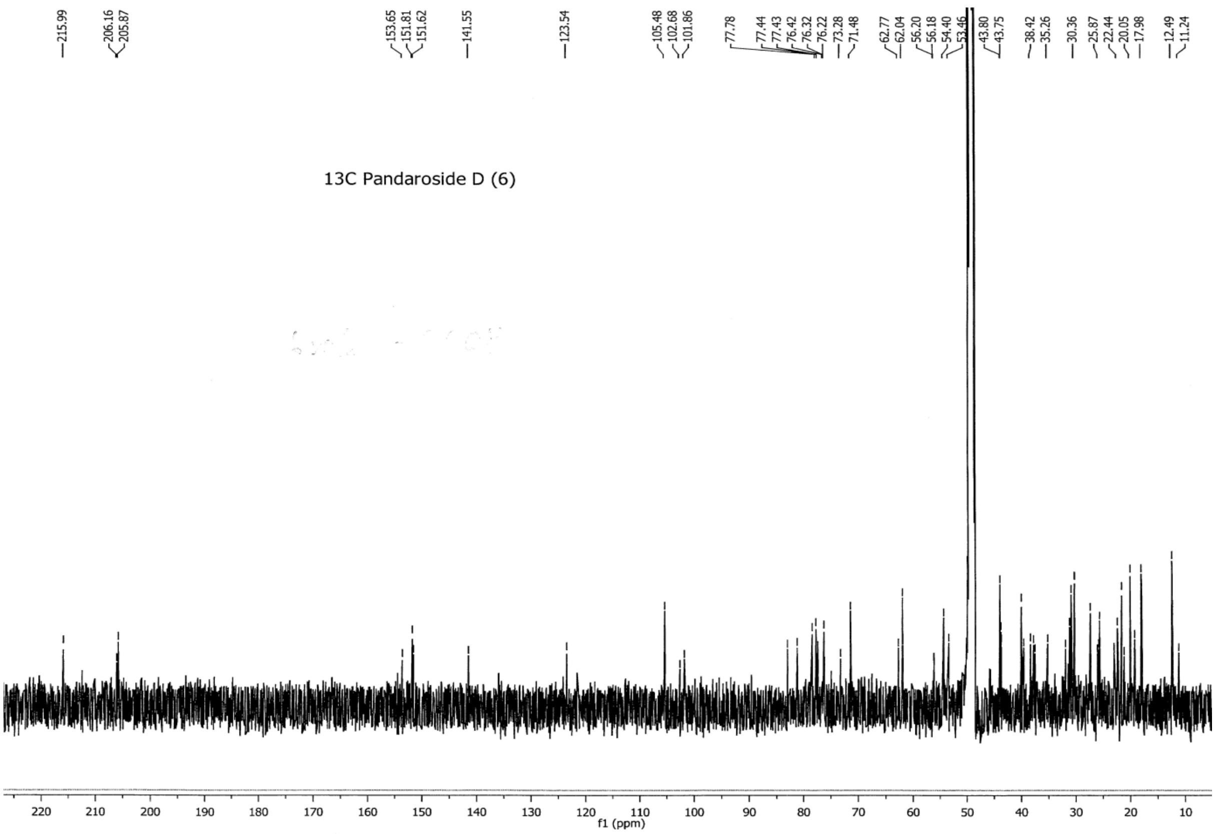

^{13}C NMR spectrum of Pandaroside Disolated from *Pandaros acanthifolium* (*Steroids* **2009**, *74*, 746–750).

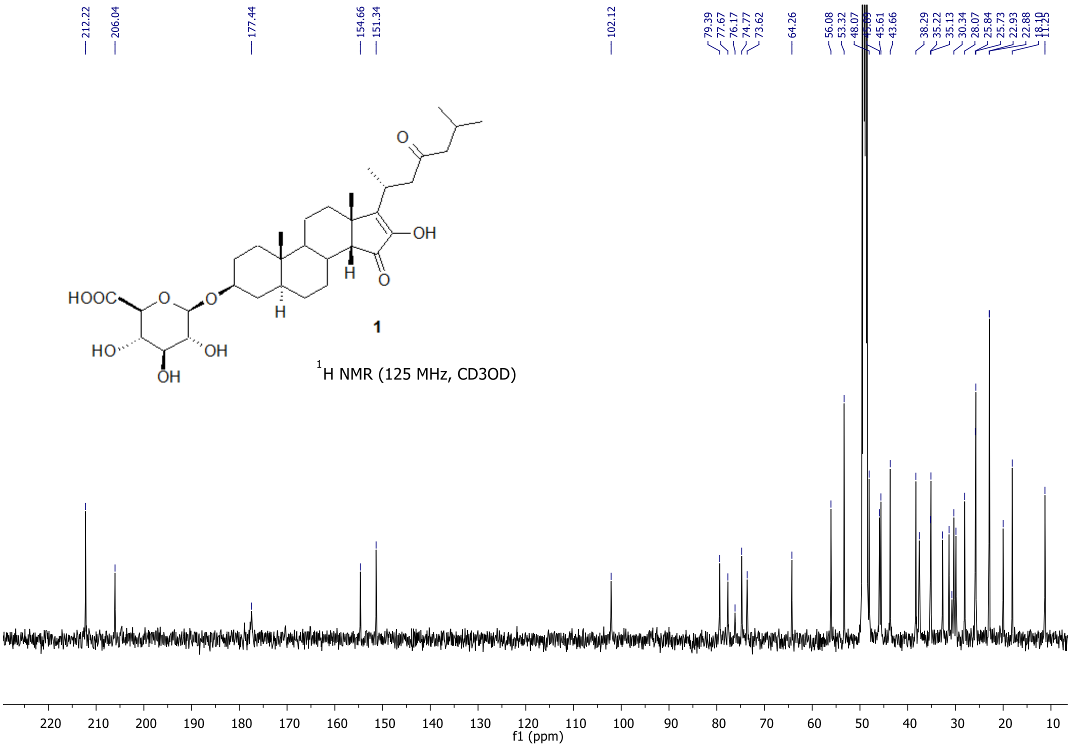

^{13}C NMR spectrum of the synthesised Pandaroside D; this work
